# Secondary structural entropy in RNA switch (Riboswitch) identification

**DOI:** 10.1186/s12859-015-0523-2

**Published:** 2015-04-28

**Authors:** Amirhossein Manzourolajdad, Jonathan Arnold

**Affiliations:** 1 Institute of Bioinformatics, University of Georgia, Davison Life Sciences Bldg, Room B118B, 120 Green St, Athens, 30602 USA; 2 National Center for Biotechnology Information (NCBI), NIH, Building 38A, RM 6S614K, 8600 Rockville Pike, Bethesda, 20894 USA; 3 Department of Genetics, University of Georgia, Davison Life Sciences Bldg, 120 Green St, Athens, 30602 USA

**Keywords:** Riboswitch, Entropy, RNA secondary structure, *cotH*, *sucC*

## Abstract

**Background:**

RNA regulatory elements play a significant role in gene regulation. Riboswitches, a widespread group of regulatory RNAs, are vital components of many bacterial genomes. These regulatory elements generally function by forming a ligand-induced alternative fold that controls access to ribosome binding sites or other regulatory sites in RNA. Riboswitch-mediated mechanisms are ubiquitous across bacterial genomes. A typical class of riboswitch has its own unique structural and biological complexity, making *de novo* riboswitch identification a formidable task. Traditionally, riboswitches have been identified through comparative genomics based on sequence and structural homology. The limitations of structural-homology-based approaches, coupled with the assumption that there is a great diversity of undiscovered riboswitches, suggests the need for alternative methods for riboswitch identification, possibly based on features intrinsic to their structure. As of yet, no such reliable method has been proposed.

**Results:**

We used structural entropy of riboswitch sequences as a measure of their secondary structural dynamics. Entropy values of a diverse set of riboswitches were compared to that of their mutants, their dinucleotide shuffles, and their reverse complement sequences under different stochastic context-free grammar folding models. Significance of our results was evaluated by comparison to other approaches, such as the base-pairing entropy and energy landscapes dynamics. Classifiers based on structural entropy optimized via sequence and structural features were devised as riboswitch identifiers and tested on *Bacillus subtilis*, *Escherichia coli*, and *Synechococcus elongatus* as an exploration of structural entropy based approaches. The unusually long untranslated region of the *cotH* in *Bacillus subtilis*, as well as upstream regions of certain genes, such as the *sucC* genes were associated with significant structural entropy values in genome-wide examinations.

**Conclusions:**

Various tests show that there is in fact a relationship between higher structural entropy and the potential for the RNA sequence to have alternative structures, within the limitations of our methodology. This relationship, though modest, is consistent across various tests. Understanding the behavior of structural entropy as a fairly new feature for RNA conformational dynamics, however, may require extensive exploratory investigation both across RNA sequences and folding models.

## Background

Non-protein-coding RNA (ncRNA) elements play an important role in biological pathways, such as gene regulation [1-4]. It has been shown that conformational features of many such RNA elements play a major part in their biological function [5,6]. In bacteria, RNA structural rearrangements can have a major effect on the expression of their downstream coding sequences (reviewed by [7]), a process known as *cis*-regulation. A classic example, and one of the earliest such elements discovered, is the complex regulatory mechanism that takes place upstream of the tryptophan operon in *Escherichia coli* during its expression [8]. Regulation of the tryptophan biosynthetic operon, however, is achieved through very different mechanisms in other organisms, such as *B. subtilis* and *Lactobacillus lactis* (reviewed by [9]). With much attention given to protein-coding genes in the past, the introduction of ncRNAs gene finders have become a relatively new area of genomic research [10]. Currently, many general-purpose [11-13] as well as ncRNA-specific gene finders, such as [14-16] are available.

### Riboswitches

An interesting group of RNA regulatory elements are riboswitches. Riboswitches are defined as regulatory elements that take part in biological pathways by selectively binding to a specific ligand or metabolite, or uncharged tRNAs, without the need for protein factors. Environmental factors such as pH [17], ion concentration [18-20], and temperature [21,22] can also trigger RNA conformational changes affecting gene regulation. Nearly all riboswitches are located in the non-coding regions of messenger RNAs [23] and are capable of regulating genes through both activation and attenuation of either transcription or translation (reviewed by [24]). Finally, other factors such as the transcription speed of RNA polymerase, the folding and unfolding rates of the aptamer of the riboswitch, and the binding rates of the metabolites add other dimensions to categorizing riboswitches. These and other factors influence the RNA switching mechanism to be kinetically or thermodynamically driven. In addition to thermodynamics-based approaches, RNA-kinetics have been gaining momentum in riboswitch-mediated regulation studies at the system level. Lin and Thirumalai [25] introduces a kinetic feedback-loop network model that describes the functions of riboswitches using experimental data from flavin mononucleotide (FMN) riboswitch.

Originally found through sequence homology upstream of bacterial coding regions [26-28], riboswitches have been shown to be more abundant than previously expected. They have also been found in cooperative or tandem arrangements [23]. It is speculated that there are at least 100 more undiscovered riboswitches in already sequenced bacterial genomes [23]. Conformational factors are essential to ligand-binding specificity of riboswitches. Many riboswitches can discriminate between similar small molecules with the aid of their structural geometry. For instance, the thiamine pyrophosphate (TPP) and S-adenosylmethionine (SAM) riboswitches measure the length of the ligand that binds to them [29-31].

### RNA secondary structure

The secondary structural topology of the RNA is very effective in scaffolding the tertiary conformation. Secondary structure mainly consists of a two-dimensional schema that depicts the base-pairing interactions within the RNA structure and is dominated by Watson-Crick base-pairing. One major computational method to predict RNA secondary structure is minimization of its free energy (MFE) within a thermodynamic ensemble, such as the Boltzmann ensemble [32,33]. State-of-the-art thermodynamic models have proven to be effective in RNA secondary structural predictions in most cases. An example of where such predictions fail would be Hammerhead type I ribozyme where loop tertiary interactions have a dominating effect on the structural conformation [34]. Centroids of the Boltzmann ensemble are also used for RNA secondary structural predictions [35]. In many cases, such a prediction is more similar to the structure inferred from comparative sequence analysis than the MFE structure is [35]. In addition, Stochastic context-free grammars (SCFG) have shown to be effective in secondary structural prediction of various RNA regulatory elements. Nawrocki and Eddy, 2013 [13] have shown that more sophisticated grammars, designed to mirror the thermodynamic models can improve the prediction accuracy of structures, once trained on known RNA structures based on maximum-likelihood criteria^a^.

Most of the discovered prokaryotic RNA regulatory elements (including riboswitches) are located upstream of the genes they regulate. They act as *cis*-regulatory elements and exhibit strong secondary structural conservation. Some exceptions to *cis*-regulation are two *trans*-acting SAM riboswitches [36] and an antisense regulation of a vitamin *B*_12_-binding riboswitch [37] in *Listeria monocytogenes*. Insights into structural and functional complexity of riboswitches already discovered are offered in [38]. Purine riboswitches are good examples of secondary structural conservation. The *add* adenine riboswitch from *V. vulnificus* and the *xpt* guanine riboswitch from *B. subtilis* have very similar secondary and tertiary conformations, despite different crystal packing interactions, pH, and Mg crystallization conditions [39]. In fact, investigation of secondary-structural homology upstream of genomic regions containing the same genes has led to the discovery of more *cis*-regulatory elements in bacteria [40,41], making them the major current approach for riboswitch identification.

The fact that riboswitch discovery is mainly based on homology makes it difficult to assess how much secondary structural conservation is expected to be prevalent in undiscovered riboswiches. Furthermore, structural homology is not always successful in finding riboswitches. Despite [42]’s rigorous sequence and structural homology searches based on the SAM-I riboswitch, the SAM-IV riboswitch could not be detected. The authors further hypothesized that the structural diversity of riboswitches could be far greater than what has been already observed. Serganov and Nudler, 2013 [38] suggest that there may not even be an interconnection between the structures of riboswitches and the nature of their cognate metabolites and consequently, the biochemical and structural information gathered so far may not be as useful in riboswitch validation as expected. The above limitations of homology-based riboswitch identification methods indicate the need for an alternative approach.

### Conformational dynamics

**Figure 1:**
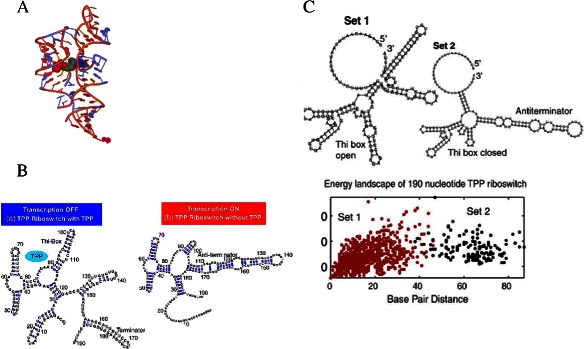
Energy Landscape of The TPP Riboswitch.**A**: Tertiary structure of an *E. coli* TPP (or *thi*-box) riboswitch bound to thiamine pyrophosphate [43]. The image was generated by the Jmol from the PDB:2hoj structure taken from the Rfam website [89]. **B**: Ligand-bound and unbound secondary structures of a TPP riboswitch in *B. subtilis*, taken from [45]. **C**: Energy landscape of the *B. subtilis* riboswitch taken from [45]. Set-1 and Set-2 clusters correspond to the two mutually exclusive secondary structures of the TPP riboswitch. Pairwise Base-pairing distance used as a measure of distance between two structures. Please refer to [45] for detailed information about the figure and clustering details.

While secondary-structure conformational features are very descriptive of many classes of riboswitches, their folding dynamics are also critical. A typical example is the TPP riboswitch which can fold into alternative structures depending on the presence of the TPP ligand. The tertiary structure stabilized in the presence of TPP is shown in Figure 1A [43]. Both the ligand-bound and the unbound secondary structures necessary for TPP riboswitch regulatory function are shown in Figure 1B. One of the major computational tools to explore possible folding trajectories is the free energy landscape. The free energy landscape was originally defined for protein folding [44]. In a typical RNA free energy landscape, possible conformations are shown with their corresponding free energy and pairwise distances from one another. In an effort to investigate the thermodynamic equilibrium of RNA folding, Quarta et al. [45] presented a case study of the energy landscape of the TPP riboswitch where the base-pairing distances between the structural possibilities form two major clusters. The clusters corresponded to native and ligand-bound structural conformations. After repeating this process for various choices of elongation of the TPP riboswitch, they showed that for certain ranges of length, each cluster corresponds to one of the two structures of the riboswitch (see Figure 1C).

In [46], the dynamics of energy landscapes across elongation of various riboswitches were investigated and it was shown that such landscapes have different clustering dynamics across kinetically and thermodynamically driven riboswitches. This work highlights the fact that even in a kinetically-driven regulation scenario, investigation of the dynamics of the thermodynamic equilibrium across the elongation can be informative. In a more recent work, energy landscape analyses led to strong evidence of evolutionary co-variation of base-pairs that favor a conserved alternative structure of the purine riboswitch [47]. In addition, prediction of structural switching in RNA has been addressed by [48,49] using abstract shapes to represent different secondary structural conformations. Freyhult et al. 2007 [50,51] examined the lowest free energy structural conformations having a certain base-pairing distance to the actual structure of the RNA to explore the structural neighbors of an intermediate, biologically active structure. A more recent work [52] presents an ingenious and a significant decrease of computational consumption of estimating the likelihood of structural neighbors. However, to date there is no computational method that can identify the diverse and structurally complex riboswitches with high confidence.

Investigation into the folding dynamics of the nascent RNA based on free energy sampling and pair-wise distances can be computationally costly. Finding a sample size that sufficiently reflects the RNA folding space behavior can be difficult and prone to model parameter biases. Furthermore, even if optimized parameters and sufficient samples were available, it would still be difficult to make comparisons across RNA elements. The latter is mainly due to the fact that the characteristics of such folding distributions (here, free energy vs. structural distance within a given ensemble of secondary structures) are not well understood.

One statistic to evaluate the distribution characteristics of any probabilistic model is the Shannon entropy [53]. While the conformation with maximum-likelihood under a given SCFG is referred to as the optimum structure under that model, all of the other sub-optimal conformations can be associated with a probability. Hence, the Shannon entropy (expected log-likelihood) of such a probabilistic folding space is $H(S)=-\sum _{s\in S} p(s)\log {p(s)}$, where *S* is the folding space containing all possible secondary structures *s* valid on the desired RNA sequence, each of which associated with the corresponding probabilities of occurrence *p*(*s*). Here, the notion of probability can also be interpreted as the frequency of occurrence of a particular conformation for the RNA sequence. Alternative formulations and approximations of Shannon entropy exist in RNA secondary structure studies, such as [54]. Exact calculations of Shannon entropy under a given SCFG as a probabilistic secondary structural folding model, however, was done in [55] and shown to be computationally convenient achievable in polynomial time *O*(*n* ^3^), where *n* is the length of the RNA sequence. In an independent work, [56] also offered an algorithm to calculate the Shannon entropy of the stochastic context-free grammar BJK [57] with parameter sets derived from a given alignment. Other measures of structural diversity such as ensemble diversity computed by RNAfold -p in the Vienna RNA Software Package [58] also exist. In this work, structural diversity is measured by the exact RNA secondary structural information theoretic-uncertainty (or here, Shannon entropy) of the complete SCFG-modeled folding space of the RNA, as computed by [55]. From hereon, we refer to this measure as structural entropy. We investigated the significance of structural entropy of RNAs with more than one biologically functional secondary structural conformation. A diverse set of prokaryotic RNA elements, validated to have such potential were used for this purpose. The performance of structural entropy to distinguish riboswitches was compared to other similar features under different negative-control sets. We then made an attempt to develop a computational method for riboswitch identification via structural entropy on a genome-wide level. The goal of the presented results of the genome-wide tests, however, is mainly exploratory and aim to investigate the genomic regions or elements that the developed method is highly sensitive to.

It has been previously shown that both high and low structural entropy values of certain classes of ncRNAs can be potentially significant. For instance, for certain riboswitches, GC-composition was co-associated with significantly high structural entropy, regardless of model accuracy to RNA secondary structure [55]. This observation raised the possibility that RNAs under selective pressure to have alternative folds, may have higher (not lower) structural entropy than expected. As discussed previously in [55], this seemingly nonintuitive observation is not theoretically impossible. The above intuition lies at the center of the proposed methodology, as will be shown.

### Our approach

#### Folding models

The folding model for which the structural entropy of the RNA is computed is very critical. SCFG folding models can be very lightweight and consist of only few grammar rules and parameters, or they can be very sophisticated consisting of thousand parameters [13,59]. In [55], it was shown that the structural entropy value is very model sensitive. On the other hand, parameters of SCFG models are usually set by maximizing their prediction accuracy using maximum-likelihood approaches. There is no guarantee, however, that folding models optimized for such criteria also preserve information about folding dynamics of such RNAs. Increasing the accuracy of folding models under current approaches may be done at the expense of altering the folding space of possible structures under that model, thus losing the information about folding dynamics of the RNA. In order to avoid potential biases in our preliminary examination, it was essential that we include models not trained to best predict secondary structure in addition to models that do. Two different SCFG models were chosen for this study, one being a structurally unambiguous SCFG model with parameters trained to best predict RNA secondary structure, and one being a structurally ambiguous model with symmetric rules and probabilities. The theoretical implications of structural ambiguity may fall outside the scope of this work and the interested reader can refer to [55]. Here, we merely treat them as two different folding models.

#### Gathering data

There is a significant amount of sequence and/or structural similarity within each class of riboswitch. This is due to the fact that these riboswitches have been discovered using sequence and/or structural homology. Here, however, we are interested in capturing the universal characteristics of RNAs with alternative fold(s), mainly riboswitches, as a basis for an identification method for conformational switches. In order for our method to be less biased towards a specific structural conformation, we avoided using homologous RNA sequences or sequences that belong to closely related organisms, where possible. We also resorted to only evaluating riboswitches that have been experimentally validated to be functional rather than computationally discovered ones. The data set gathered in this work is a compromise between the above considerations and the need to include a diverse set of riboswitches in our data set. Although the attempt to computationally extract a universal feature from the diversity of prokaryotic riboswitches each having unique structural and biological characteristics is a great oversimplification, it serves as a common ground for comparing various features that aim to capture the RNA conformational dynamics as a whole.

#### Negative controls

One of the main challenges of our test, was the preparation of a reliable negative control. Folding models deployed here are very lightweight and simplistic, giving rise to potential unrelated dependencies to the factors such as genomic composition of RNA sequence. Therefore, gathering real biological sequences that are as similar to RNA sequences as possible while not having potential for alternative fold(s) is very critical to the significance of our test. Here, we relied on the following sets of negative controls: 1. dinucleotide shuffles of riboswitches (generated using [60]), 2. Mutagenesis; Structural mutants of the gathered sequences experimentally tested for not being functional, 3. The reverse complements (or antisense sequences) of gathered riboswitches, and 4. Sequences of the non-coding regions that are likely to be riboswitches. The choice of antisense as a negative control is explained in the Methods section.

#### Comparison to other methods

Two additional measures of structural diversity were used to assess the significance of structural entropy values in collected data. The first measure was the base-pairing entropy [54] of the BJK model *BJKbp* as defined in ([61] Eq. 3). For more information see the Methods section. The second measure, denoted as *Sil*, was obtained from clustering the RNA energy landscape. The *Sil* value reflects how well the energy landscape clusters into two. Calculations for *Sil* were according to [46]. We then compared the performance of classifiers designed to distinguish riboswitches from various negative controls. In order to evaluate the performance of structural entropy to detect alternative fold, we compared it to measures from RNAShapes [49] and FFTbor [52] predictions. These measures corresponded to energy disequilibrium of alternative folds: *p*1/*p*2, where *p*1 was the highest value in the predictions of the corresponding software and *p*2 was the second highest value. For RNAShapes, *p*1 is the probability of the most likely abstract shape of structure, whereas *p*2 is the second most. For FFTbor, *p*1 is the probability of the MFE structure and *p*2 is the probability of an alternative folding scenario where the structure has a particular base-pair distance with the MFE structure. Features used in this work are shown in Table 1. Please see the Methods section for further details.

**Table 1:** **List of various sequence and structural features used throughout the work**

**Feature**	**Cardinality**
RND	Real positive
BJK	Real positive
BJKbp	Real positive
Sil	Real positive
FFTbor	Real positive
RNAShapes	Real positive
GC	Real positive less than one
MFE	Real negative
CFE	Real negative
L	Real positive

## Results and discussion

The two lightweight SCFG folding models used to calculate structural entropy are denoted here as BJK and RND models, which are taken from the literature (Please see the Methods section). RNA encoded sequence from Bacteria validated to have potential for two alternative folds were gathered from the literature (see Table 2) as representatives of RNAs having potential for alterative folding. This generally consisted of riboswitches and some other ribo-regulators, although we refer to all these sequences as riboswitches, here. A subset of such sequences were selected as the positive control set of sequences having two structures. The criterion for selecting such a subset was minimum length of the RNA that exhibits alternative folds for each sequence. This criterion is further explained in Methods. The resulting set of length variant sequences are described in Tables 2 and 3.

**Table 2:** **Data collection**

**ID**	**Riboswitch**	**Organism (P/N)**	**Alteration**	**Grouping**	**References**
ID01	Alpha Operon	*Escherichia coli* (N)	Slow-fast	Train	[90,91]
ID02	ATP	*Bacillus subtilis* (P)	Enzyme	Test	[92]
	ATP^[1]^	*Salmonella* (N)	Enzyme	None	[73]
ID03	c-di-GMP	*Geobacter sulfurreducens* (N)	Ligand	Train	[40]
ID04	c-di-GMP	*Candidatus Desulforudis* (P)	Ligand	Test	[93]
ID05	Cobalamin	*Escherichia coli* (N)	Ligand	Train	[27]
ID06	Cobalamin	*Bradyrhizobium japonicum* (N)	Ligand	Train	[94]
ID07	Cobalamin	*Salmonella* (N)	Ligand	Test	[95]
	D. peptide^[2]^	*Synechococcus sp. CC9902* (N)	Motif	None	[96]
ID08	Fluoride	*Pseudomonas syringae* (N)	Ligand	Train	[97]
ID09	Fluoride	*Thermotoga petrophila* (N)	Ligand	Train	[98]
ID10	Fluoride	*Bacillus cereus* (P)	Ligand	Test	[97]
ID11	FMN	*Fusobacterium nucleatum* (N)	Ligand	Train	[99,100]
ID12	FMN	*Escherichia coli* (N)	Ligand	Train	[20,101]
ID13	FMN	*Bacillus subtilis* (P)	Ligand	Test	[99-101]
	glmS	*T. tengcongensis* (N)	None	None	[75,76,102,103]
	glnA	*Synechococcus elongatus* (N)	Motif	None	[96]
ID14	Glycine	*Fusobacterium nucleatum* (N)	Ligand	Train	[104-106]
ID15	Glycine	*Bacillus subtilis* (P)	Ligand	Test	[104]
	Hammerhead I	*Schistosoma Mansoni* (-)	None	None	[34,107]
	Hammerhead II	*Marine metagenome* (-)	None	None	[108]
ID16	Lysine	*Thermotoga maritima* (N)	Ligand	Train	[109,110]
ID17	Lysine	*Bacillus subtilis* (P)	Ligand	Test	[110]
ID18	Magnesium	*Salmonella enterica* (N)	Mg2^+^	Train	[18,20]
ID19	Magnesium	*Escherichia coli* (N)	Mg2^+^	Train	[18]
ID20	Magnesium	*Bacillus subtilis* (P)	Mg2^+^	Test	[19]
ID21	Moco	*Escherichia coli* (N)	Ligand	Train	[111]
ID22	pH-responsive	*Escherichia coli* (N)	pH	Train	[17]
ID23	pH-responsive	*Serratia marcescens* (N)	pH	Test	[17]
ID24	preQ1 II	*Streptococcus pneumoniae* (P)	Ligand	Train	[40,112]
ID25	preQ1 I	*Bacillus subtilis* (P)	Ligand	Test	[113]
ID26	Purine (Adenine)	*Vibrio vulnificus* (N)	Ligand	Train	[39]
ID27	Purine (Adenine)	*Bacillus subtilis* (P)	Ligand	Test	[39]
ID28	Purine (Guanine)	*Bacillus subtilis* (P)	Ligand	Test	[39,114]
ID29	ROSE-1	*Bradyrhizobium japonicum* (N)	Heat	Train	[21,22]
ID30	ROSE-2	*Escherichia coli* (N)	Heat	Train	[21]
ID31	ROSE-2387	*Mesorhizobium loti* (N)	Heat	Test	[21]
ID32	ROSE-N1	*Rhizobium* (N)	Heat	Test	[21]
ID33	ROSE-P2	*Bradyrhizobium* (N)	Heat	Train	[21]
ID34	SAH	*Ralstonia solanacearum* (N)	Ligand	Train	[40,115]
ID35	SAM-I	*T. tengcongensis* (N)	Ligand	Train	[31]
ID36	SAM-I	*Bacillus subtilis* (P)	Ligand	Test	[116-119]
ID37	SAM-II	*Agrobacterium tumefaciens* (N)	Ligand	Train	[120]
ID38	SAM-III (SMK)	*Streptococcus gordonii* (P)	Ligand	Train	[121]
ID39	SAM-III (SMK)	*Enterococcus faecalis* (P)	Ligand	Test	[121-123]
ID40	SAM-IV	*Streptomyces coelicolor* (P)	Ligand	Train	[42]
ID41	SAM-IV	*Mycobacterium tuberculosis* (P)	Ligand	Test	[42]
ID42	SAM-SAH	*Roseobacter* (N)	Ligand	Train	[41]
ID43	SAM-SAH	*Oceanibulbus indolifex* (N)	Ligand	Test	[41]
ID44	SAM-V	*Cand. P. ubique* (N)	Ligand	Train	[124]
ID45	SAM-V	*Cand. P. ubique* (N)	Ligand	Test	[125]
ID46	THF	*Eubacterium siraeum* (P)	Ligand	Train	[126,127]
ID47	THF	*Clostridium kluyveri* (P)	Ligand	Test	[126]
ID48	TPP	*Escherichia coli* (N)	Ligand	Train	[30,128-130]
ID49	TPP	*Bacillus subtilis* (P)	Ligand	Test	[26,129]
ID50	Tryptophan	*Escherichia coli* (N)	Complex	Train	[8,131]
ID51	Tryptophan	*Bacillus subtilis* (P)	Complex	Test	[132,133]
ID52	Tuco	*Geobacter metallireducens* (N)	Ligand	Test	[111]
	yxkD	*Bacillus subtilis* (P)	Motif	None	[76]

Collected sequences from literature observed to have more than one secondary structure. P corresponds to gram-positive and N corresponds to gram-negative. Genomic locations are available in Table 3.

^[1]^Table 2: This sequence overlaps codons. pH also has a role in alteration of structure.

^[2]^Table 2: Downstream-peptide.

**Table 3:** **Genomic locations of collected sequences**

**ID**	**Accession**	**Start**	**End**	**Strand**	**Length**
ID01	U00096.3	3442440	3442547	-	108
ID02	NC_000964.3	486099	486230	+	132
ID03	AE017180.2	2773395	2773492	+	98
ID04	CP000860.1	1860063	1860186	-	124
ID05	U00096.3	4163564	4163632	+	69
ID06	BA000040.2	5279368	5279482	-	115
ID07	AE006468.1	2113803	2113897	-	95
ID08	CP000075.1	1675079	1675157	-	79
ID09	CP000702.1	1794825	1794895	+	71
ID10	AE017194.1	4815592	4815665	+	74
ID11	AE009951.2	2496	2668	+	173
ID12	U00096.3	3184455	3184718	-	264
ID13	NC_000964.3	2431380	2431615	-	236
ID14	AE009951.2	963901	963988	-	89
ID15	NC_000964.3	2549381	2549501	-	121
ID16	AE000512.1	1519015	1519250	-	236
ID17	NC_000964.3	2910878	2911045	-	170
ID18	CP001363.1	4712312	4712483	+	172
ID19	U00096.3	4467416	4467525	+	110
ID20	NC_000964.3	1395622	1395825	+	204
ID21	U00096.3	816923	817041	+	119
ID22	U00096.3	3238486	3238569	+	84
ID23	CP003959.1	4635235	4635309	+	75
ID24	AE007317.1	904178	904257	+	80
ID25	NC_000964.3	1439279	1439338	+	60
ID26	AE016796.2	504379	504491	+	113
ID27	NC_000964.3	626329	626426	-	98
ID28	NC_000964.3	2320055	2320196	-	142
ID29	U55047.1	3107	3215	+	109
ID30	U00096.3	3867416	3867488	-	73
ID31	BA000012.4	1943727	1943820	-	94
ID32	AY316747.1	197909	198004	+	96
ID33	AP012279.1	5017601	5017677	-	135
ID34	AL646052.1	1348529	1348625	+	97
ID35	AE008691.1	1750249	1750372	-	124
ID36	NC_000964.3	1180646	1180802	-	157
ID37	AE007869.2	2703460	2703559	+	100
ID38	CP000725.1	1038292	1038371	+	80
ID39	CP003726.1	618415	618496	+	82
ID40	NC_003888.3	2308634	2308770	-	137
ID41	AE000516.2	3723565	3723713	+	149
ID42	AAYC01000001.1	142052	142099	+	48
ID43	ABID01000011.1	17036	17084	-	49
ID44	CP000084.1	1005827	1005879	+	53
ID45	CP000084.1	1127359	1127423	-	65
ID46	FP929059.1	95139	95281	-	144
ID47	NC_009706.1	3903929	3904072	+	144
ID48	U00096.3	2185279	2185426	-	148
ID49	NC_000964.3	1242265	1242422	+	158
ID50	U00096.3	1322975	1323055	-	81
ID51	NC_000964.3	2377419	2377559	-	141
ID52	CP000148.1	1157816	1157926	-	111

Column ID corresponds riboswitches in Table 2.

### Mutagenesis

To investigate the relationship of various structural features to the folding space of the riboswitches, we compared their wild-type value to that of structural and non-structural mutants. By *structural* mutants, we mean those mutant sequences that were designed to disrupt either of the two biologically functional conformations of the riboswitch. These structural mutants, whose regulatory functions had been experimentally investigated, were gathered from the literature. These mutant sequences may not have been naturally occurring biological sequences. Nevertheless, having very similar sequence features to their wild type, this enables us to evaluate the variations of structural features with respect to loss of functionality given closest possible negative controls. The percentage of change in feature values for mutants relative to the wild type is shown in Table 4. If there is a relationship between the features and alternative folds, one would expect the values corresponding to structural mutants (denoted as YES) to be significantly less than that of the wild type and non-structural mutants (denoted as NO). A simplified criterion to calculate the performance of each feature was to define true positives as negative values in structural mutants and true negatives as zero or positive values in non-structural mutants. Hence, we calculated sensitivity and specificity of each feature to structural mutants under the above criterion. Here, Sensitivity and Specificity symbolize the performance of a classifier that, based on the diversity value of the wild type and a non-functional mutant, predicts if the mutant is a structural mutant (denoted YES) or not. The classifier rule here is that structural mutants must have a lower value. Performance of each feature is shown in Table 5. The performance of the base-pairing entropy *BJKbp* is higher than other features on average. This suggests that structural mutants are expected to have lower base-pairing entropy than non-structural mutants and wild type 83.33 percent of the times, while non-structural mutants are expected to equal or higher values than the while type 83.33 of the times. The performance of the structural entropy under the same folding model *BJK* was slightly lower, while being higher than those for the *RND* and *Sil* features. Features *BJKbp*, *BJK*, and *RND* corresponding to the *B. subtilis* Magnesium structural mutants M5 and M6 were all positive, implying that our hypothesis of higher structural entropy and alternative fold does not hold for this riboswitch. The average silhouette index of energy landscapes (*Sil*) has a much better performance for the mentioned riboswitches. This could either be because SCFG models fail to capture conformational dynamics of this riboswitch or the thermodynamic equilibrium between its alternative folds is more subtle than expected.

**Table 4:** **Mutagenesis**

**Wild-type**	**Riboswitch (Length)**	**Organism**		**Sensitivity %**	**Specificity %**	
**ID49**	**TPP (158)**	***B. subtilis***		**56.9**	**51.8**	
Mutants [26]	Function	Disruption of only *one* structure	*Δ*RND %	*Δ*BJK %	*Δ*BJKbp %	*Δ*Sil %
+30	Disrupts anti-antiterminator	Yes	0.7	-2.6	-3.9	-55.2
+118	Disrupts anti-terminator	Yes	-0.4	5.3	-0.7	-50.3
+80	Disrupts thi-box	No	0.8	3.3	0.8	-38.2
+97	Disrupts thi-box	No	-0.8	1.9	1.6	-63.2
**Wild-type**	**Riboswitch (Length)**	**Organism**		**Sensitivity %**	**Specificity %**	
**ID13**	**FMN (236)**	***B. subtilis***		**81.8** ^**[1]**^	64.3	
Mutants [26]	Function	Disruption of only *one* structure	*Δ*RND %	*Δ*BJK %	*Δ*BJKbp %	*Δ*Sil %
G34C/G35C	Disrupts anti-terminator	Yes	-1.6	-5.5	-2.4	15.4
C86T	Disrupts rfn-box	No	0.2	-0.1	0.6	11.8
C49T	Disrupts rfn-box	No	0.3	0.5	0	-14.3
G157A/G160A	Disrupts anti-antiterminator	Yes	0	-0.7	-0.9	66.7
**Wild-type**	**Riboswitch (Length)**	**Organism**		**Sensitivity %**	**Specificity %**	
**ID36.1** ^**[2]**^	**SAM-I (159)**	***B. subtilis***		**94**	**88.7**	
Mutants [134]	Function	Disruption of only *one* structure	*Δ*RND %	*Δ*BJK %	*Δ*BJKbp %	*Δ*Sil %
Ma	Disturbs both structures	No	2.3	15.8	10.7	-48.8
Mab	Disrupts anti-terminator	Yes	-2.3	-0.29	-0.4	4.1
Mc	Disrupts anti-terminator	Yes	0.3	-0.31	-0.8	-0.3
Mabc	Compensates mutations to wild type	No	-1.1	-0.32	-0.7	-3.2
**Wild-type**	**Riboswitch (Length)**	**Organism**	**Reference**	**Sensitivity %**	**Specificity %**	
**ID18**	**Magnesium (172)**	***Salmonella enterica***		**64.5** ^**[3]**^	**43.5**	
Mutant [20]	Function	Disruption of only *one* structure	*Δ*RND %	*Δ*BJK %	*Δ*BJKbp %	*Δ*Sil %
C145G	Favors high Mg2^+^ conformation	Yes	1.7	-1.8	-4.7	-10.1
Mutants [20]	Function	Disruption of only *one* structure	*Δ*RND %	*Δ*BJK %	*Δ*BJKbp %	*Δ*Sil %
M1	Favors +FMN conformation	Yes	0.4	-3.8	-5.8	-43
M2	Favors -FMN conformation	Yes	-1.4	-1.9	-1.2	-5.7
**Wild-type**	**Riboswitch (Length)**	**Organism**		**Sensitivity %**	**Specificity %**	
**ID20**	**Magnesium (204)**	***B. subtilis***		**78**	**65**	
Mutants [19]	Function	Disruption of only *one* structure	*Δ*RND %	*Δ*BJK %	*Δ*BJKbp %	*Δ*Sil %
M5	Disrupts termination	Yes	2.7	0.9	0.7	-12.3
M6	Distrupts anti-terminator	Yes	3.9	12.4	8	-14.8
**Wild-type**	**Riboswitch (Length)**	**Organism**		**Sensitivity** %	**Specificity %**	
**ID33**	**ROSE-P2 (135)**	***Bradyrhizobium***		**22.7** ^**[4]**^	**22.2**	
Mutant [22]	Function	Disruption of only *one* structure	*Δ*RND %	*Δ*BJK %	*Δ*BJKbp %	*Δ*Sil %
*Δ*G83^[5]^	Deletion of a critical nucleotide	Yes	-2.6	-8.1	-4.7	8.6

Percentage of change in entropy values of mutants compared to wild type. Mutation names are according to the literature. Type of disruption to wild type activity/conformation is denoted in column function (please see references for more detail on mutation information). Mutants have same length as the wild type, except for the ROSE-P2 thermosensor. Wild-type segments are the same as gathered data, except for the SAM-I riboswitch where a homologue has been used. *Δ*RND% and *Δ*BJK%, refer to structural entropy values for the RND and BJK models, respectively. *Δ*BJKbp% refers to the base-pairing entropy of the BJK model as defined by [54]. *Δ*Sil% refers to the two-cluster average silhouette index of the energy landscape of the RNA as calculated by [46]. Sensitivity% and specificity% refer to BJK model accuracy to the secondary structural conformation, with disregard to pseudoknots.

^[1]^Table 4: Two out of the 55 base-pairings of the *B. subtilis* FMN sequence are G-A pairs.

^[2]^Table 4: ID36.1 is the *metI* SAM-I riboswitch in *B. subtilis* and has sequence identity of 76% with ID36 *yitJ* *B. subtilis* SAM-I riboswitch using BLASTⒸ. Sequence location on Location on the *B. subtilis* str. 168 strain embAL009126.3 (1258304-1258462), forward strand.

^[3]^Table 4: CYK structural prediction under the BJK model and that of the MFE model via viennaⒸdetect different alteration of the Magnesium riboswitch in *Salmonella enterica* serovar Typhimurium. Structural distance of the MFE prediction to the high Mg2^+^ and low Mg2^+^ structures are 28 and 120, respectively while they are 114 and 74, under CYK-based structural prediction of the BJK model. Sensitivity and specificity values for the BJK model prediction of the low Mg2^+^ conformation are 22% and 22%.

^[4]^Table 4: One out of the 44 base-pairings of the *Bradyrhizobium* ROSE-P2 sequence is a G-G pair.

^[5]^Table 4: The *Δ*G83 mutant is one nucleotide shorter than the ROSE-P2 135nt-long wild type.

**Table 5:** **Mutagenesis results**

**Feature**	**Sensitivity (%)**	**Specificity (%)**	**MCC**
BJKbp	83.33	83.33	0.645
BJK	75	66.67	0.403
RND	41.67	66.67	0.08
SIL	66.67	16.67	-0.175

Classification rule: Lower value than wild type predicts structural mutant, while higher or equal value predicts non-structural mutants. Positive control: Structural Mutants of a given wild type. Negative control: non-structural mutants of a given wild type. See the Methods section for details on calculating sensitivity and specificity.

### Sense-antisense classification results

Classification of the RNA sequence into riboswitches and antisense sequences was done using binomial logistic regression. Sequence features, such as Length *L*, Minimum Free Energy *MFE*, GC-composition *GC*, and structural entropy were used for classification. The MFE value was included as a relative measure of structural stability. An initial investigation of the power of selected features in sense-antisense discrimination was done through cross-validation for all 104 (52 riboswitches and 52 antisense) sequences. Binomial logistic regression classification probabilities were assigned to each sequence based on the other 104 sense and antisense sequences. It is shown in Table 6 that features {*L,GC,GU,Sil*} result in the highest true positive rate, lowest false positive rate, and highest area under the receiver operating characteristic (ROC) curve. This result suggests that the folding space of the riboswitch sequence is expected to be different than that of its antisense, since the *Sil* feature is based on the clustering of the energy landscape, although further investigation into this assumption is needed.

**Table 6:** **Classification performance using cross validation**

**Classifier**	**TP rate**	**FP rate**	**MCC**	**R.O.C. area**
{*L,GC,GU,Sil*}	0.750	0.250	0.500	0.826
{*L,GC,GU*}	0.673	0.327	0.346	0.700
{*L,GC,GU,BJK*}	0.644	0.356	0.289	0.691
{*L,GC,GU,BJKbp*}	0.654	0.346	0.309	0.690
{*L,GC,GU,RND*}	0.654	0.346	0.308	0.689
{*L,MFE,GC,GU,RND*}	0.673	0.327	0.346	0.714
{*L,MFE,GC,GU*}	0.654	0.346	0.308	0.707
{*L,MFE,GC,GU,BJK*}	0.663	0.337	0.327	0.703
{*L,MFE,GC,GU,BJKbp*}	0.663	0.337	0.327	0.701
{*L,MFE,GC,GU,Sil*}	0.625	0.375	0.250	0.697
{*L,MFE,GU*}	0.663	0.337	0.327	0.710
{*L,MFE,GU,RND*}	0.663	0.337	0.327	0.702
{*L,MFE,GU,BJKbp*}	0.663	0.337	0.32	0.701
{*L,MFE,GU,Sil*}	0.654	0.346	0.308	0.701
{*L,MFE,GU,BJK*}	0.644	0.356	0.289	0.699
{*L,MFE,GC,RND*}	0.663	0.337	0.327	0.708
{*L,MFE,GC,BJK*}	0.663	0.337	0.327	0.703
{*L,MFE,GC,BJKbp*}	0.635	0.365	0.269	0.683
{*L,MFE,GC*}	0.606	0.394	0.212	0.650
{*L,MFE,GC,Sil*}	0.635	0.365	0.270	0.644
{*L,MFE,GCU,RND*}	0.644	0.356	0.289	0.693
{*L,MFE,GCU,BJK*}	0.625	0.375	0.250	0.617
{*L,MFE,GCU,BJKbp*}	0.596	0.404	0.193	0.595
{*L,MFE,GCU*}	0.587	0.413	0.174	0.581
{*L,MFE,GCU,Sil*}	0.548	0.452	0.097	0.554

104-fold binomial logistic classifiers on all of the 52 riboswitch sequences and their antisense sequences. classifier features shown in legend. WekaⒸopen source software package used. Features *L,MFE,GC,GU,GCU* and *U* denote length, MFE, GC-composition, and uracil frequency, respectively. Features *RND* and *BJK* denote structural entropy of the RND and BJK models, respectively. as defined in [54]. Feature *BJKbp* denotes base-pairing entropy as defined in [54]. Feature *Sil* denotes the two-cluster average Silhouette index of energy landscape as calculated in [46].

**Figure 2:**
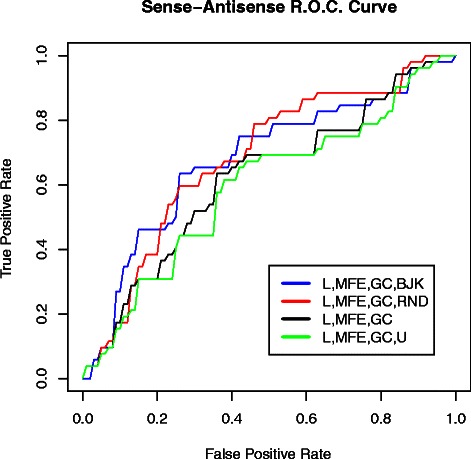
Classification ROC curve. ROC curves of 104-fold binomial simple logistic classifiers on all the 52 riboswitch sequences and their antisense sequences. classifier features shown in legend. WekaⒸopen source software package used to assess probability distributions. Resolution (0.01) was used to calculate true and false positive rates. Corresponding threshold for a given false positive rate was used to calculate the true positive rate. Values strictly higher than threshold were used to calculate true positive rates.

The performance of classifiers that involved uracil composition were more dependent on sequence features rather that structure and subsequently more prone to data fitting. The reason is that uracil composition can be different the sense and antisense. Excluding classifiers that incorporate uracil composition (i. e. forth set of rows in Table 6) showed that the features sets {*L,MFE,GC,RND*} and {*L,MFE,GC,BJK*} had a fairly acceptable performance. The performance of the corresponding feature sets were higher than the {*L,MFE,GC*} classifier. Furthermore, inclusion of uracil composition into the classifier lowered performance (See {*L,MFE,GC,U*} in Figure 2). It is noteworthy to recall that the above classifiers neither represent the most informative features of the data nor are they tuned for best performance (Please refer to Methods section for details on calculating performance). Therefore, structural entropy may be informative in sense-antisense classification since *L* and *GC* are equal for each pair of sense and antisense. The performance of {*L,MFE,GC,BJKbp*} was also higher than {*L,MFE,GC*} but slightly lower than feature sets that incorporate structural entropy values. The ROC curve corresponding to these classifiers is shown in Figure 2.

The sense-antisense results show that the above approach has a high false positive rate, should it be used for riboswitch discovery. It also does not fully address all questions about its performance. For instance, is structural entropy orthogonal to results of other available methods that aim to capture conformational switches, or is it highly correlated with them? What is the significance of incorporating features *L*, *GC*, and *MFE* in the classifiers? How much of the performance of the above classifiers, such as {*L,GC,GU,Sil*}, reflect structural characteristics of the riboswitches and how much is due to other features of the data? How generalizable are the results and what is the performance of classification when tested on riboswitches from distant organisms with very different genomic compositions? Can we distinguish riboswitches from their antisense and other similar sequences with no structure, simultaneously? Does the structural entropy of a typical riboswitch tend to be lower or higher than that of its antisense sequence? To address the above questions, first we calculated the correlation between structural entropy features and other results from gathered tools. For this purpose, we performed the correlations for all riboswitches and their antisense sequences, totalling 104 sequences. Correlation values between structural entropy and other conformational features are illustrated in Table 7 for both folding models, RND and BJK, along with correlation values corresponding to structural entropy normalized to sequence length. By inspection, we can see that structural entropy is not necessarily highly correlated with other features, suggesting the possibility that it may contain additional information about RNA sequences, in general. We neither rigorously calculated the significance of correlation values nor did we further evaluate the orthogonality of structural entropy to other features. We then selected classifiers {*L,GC,GU,Sil*}, {*L,MFE,GC,RND*}, {*L,MFE,GC,BJK*}, and {*L,MFE,GC*} for further investigation. They are referred to as LGCGUSIL, LMFEGCRND, LMFEGCBJK, and LMFEGC. We divided our data into training and test sets, each having different average GC-composition. Sequence segments and their corresponding structures are included in sections Training set and Test set in Appendix. We then evaluated the performance of the classifiers from the training set using the test set.

**Table 7:** **Correlations between entropy values and other approaches**

**Correlations**	**FFTbor**	**RNAShapes**	**Sil**
RND	-0.46	-0.12	-0.42
BJK	-0.5	-0.18	-0.46
RND Normalized	-0.29	-0.15	-0.34
BJK Normalized	-0.34	-0.36	-0.41

RND and BJK are the structural entropy values for corresponding models. Normalized RND or BJK are corresponding values normalized to sequence length. FFTbor, RNAShapes, and Sil are other conformation features calculated according to Methods section.

#### Random shuffles

**Figure 3:**
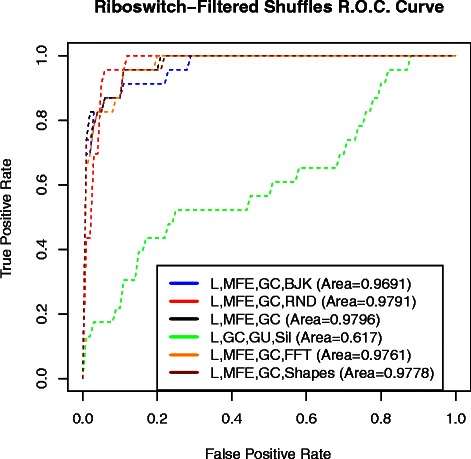
Classification ROC Curve. ROC curves of binomial logistic classifiers on the riboswitches of the test set (23 sequences) and their filtered random shuffles (151 sequences). Classifier features shown in legend. SPSSⒸwas used to estimate logistic regression coefficients derived from the training set consisted of the 29 riboswitches and 216 sequences as negative control. Resolution (0.01) was used to calculate true and false positive rates. Corresponding threshold for a given false positive rate was used to calculate the true positive rate. Values strictly higher than threshold were used to calculate true positive rates. In the legend, the area under the ROC curve corresponding to each classifier is shown.

First, in order to assess the relationship between the performance of the above binomial-logistic-regression classifiers to structural features, we performed dinucleotide shuffling test [60]. We originally generated 10 dinucleotide shuffles for each of the riboswitches in the training and test sets. We used riboswitches of the training set and their corresponding random shuffles to estimate binomial logistic-regression coefficients. We then used the coefficients to classify sequences of the test set and their corresponding random shuffles. However, the classification performance and corresponding ROC curves were highly dependent on the MFE feature (data not shown). In order to both have a better insight into the structural entropy feature and a more clear comparison to other methods, we then filtered both the random shuffles of the training and test sets to have similar MFE values to their corresponding riboswitches and repeated the test (please see Methods section and Table 8 for information on the filtered dinucleotide shuffles). The ROC curves and performance values can be seen in Figure 3 and Table 9, respectively. The classifier LGCGUSIL poorly distinguishes riboswitches from random shuffles compared to the other classifiers. This suggests that the high performance of this classifier in sense-antisense classification was not necessarily due to structural features. Although the RNAShapes classifier has higher performance as shown in Table 8, it only corresponds to one point of the ROC curve. In order to have a more comprehensive measure of performance, we calculated the area under the ROC curve for the classifiers. The performance of most classifiers is roughly similar with the LMFEGCRND having the second highest value after LMFEGC (see Figure 3 and area under ROC curve in the legend). Although filtering only for those dinucleotide shuffles having similar MFE would seem a reasonable negative control, we did not further investigate random shuffle test. Preparing a random-shuffle negative control ensemble of sequences with similar length, composition, and MFE values that homogeneously represent all riboswitches may be a formidable task and not necessarily helpful with evaluating our approach that focuses on *real* biological sequences as negative control.

**Table 8:** **Average and standard deviation values of Length, MFE, and GC-compositions of the training and test sets along with their filtered dinucleotides shuffles**

**Train**	**L**	**MFE**	**GC**	**std(L)**	**std(MFE)**	**std(GC)**
Riboswitch	114.10	-41.05	0.52	49.27	23.83	0.10
Shuffles	127.0	-38.19	0.55	47.85	18.51	0.09
**Test**	**L**	**MFE**	**GC**	**std(L)**	**std(MFE)**	**std(GC)**
Riboswitch	120.74	-44.63	0.49	46.71	18.60	0.09
Shuffles	141.77	-40.09	0.51	38.97	11.97	0.08

Column Riboswitch denotes riboswitches. Column Shuffles denotes filtered dinucleotide shuffles. Total number of filtered dinucleotide shuffles are 216 and 151, in the training and testing sets, respectively.

**Table 9:** **Classification performance in dinucleotide shuffling test**

**Classifier**	***TP%***	***FP%***
LMFEGCBJKbp	65.2	0.7
LMFEGCBJK	69.6	0.7
LMFEGCRND	69.6	3.3
LMFEGC	69.6	0.7
LGCGUSIL	0.0	0.0
LMFEGCFFT	65.2	1.3
LMFEGCShapes	73.9	0.7

Actual length of sequences used in this test. Column Features denotes features used from the training set. *T* *P* *%* denotes percentage of true positives. *F* *P* *%* represent the percentages of dinucleotide shuffles that are misclassified as riboswitches. Please see Methods section for details on preparing dinucleotide shuffles.

To evaluate the significance of feature *L*, we considered both constant choice of length and variable choice of length for riboswitches. Apart from the antisense sequence, untranslated regions (UTR) shorter than 80 nt have been selected as another negative set, since they are unlikely to contain structures over such a short length. Some riboswitch sequence segments, however, were selected to be shorter than this length. The length of the corresponding UTR (from transcription binding site to the translation start codon) for riboswitches, however, were not shorter than 80 nt. UTRs corresponding to the *σ*-70 in *E. coli* with distance less than 80 nt from the translation start codon were used here as sequences that do not contain structure. 30 sequence segments were selected from *σ*-70 *E. coli* UTRs shorter than 80 nt (see Table 10 for information on sequence locations). The section Methods extensively discusses the criteria for selecting the subsets, dividing the riboswitches and *E. coli* UTRs into training/test sets, as well as information on data sets. Average and standard deviation of features *L*, *MFE*, and *GC* for the training and test sets are shown in Table 11. The free energy of the centroid structure [35] calculated by CentroidFoldⒸ [62], denoted here as CFE, was also used as a substitute for *MFE*.

**Table 10:** **Short UTR collection**

**Start**	**End**	**Strand**	**Gene**	**Length**
42325	42403	+	*fixA*	79
246641	246712	+	*yafL*	72
570070	570116	+	*ybcL*	47
848134	848173	-	*dps*	40
879876	879950	+	*dacC*	75
989579	989637	-	*pncB*	59
1108480	1108558	+	*mdoG*	79
1331812	1331879	+	*cysB*	68
1397550	1397576	-	*fnr*	27
1570069	1570096	-	*gadB*	28
1732381	1732459	+	*mepH*	79
1927731	1927756	-	*yebE*	26
2039370	2039399	+	*zinT*	30
2268700	2268748	+	*rtn*	49
2380676	2380735	+	*elaD*	60
2541550	2541579	-	*cysP*	30
2823813	2823854	+	*srlA*	42
2982146	2982216	-	*kduI*	71
3134393	3134425	-	*pitB*	33
3276888	3276936	+	*kbaZ*	49
3467875	3467918	-	*chiA*	44
3651959	3651984	+	*slp*	26
3735493	3735520	+	*malS*	28
3845190	3845221	-	*uhpT*	32
3909548	3909591	-	*pstS*	44
4028994	4029036	-	*fadB*	43
4213425	4213501	+	*aceB*	77
4244442	4244487	-	*malE*	46
4358054	4358129	-	*cadB*	76
4492620	4492646	+	*indK*	27

30 randomly chosen untranslated regions of lengths less than 80 nt corresponding to the *σ*-70 transcription factor binding sites in *Escherichia coli* str. K-12 substr. MG1655 (GenBankⒸID: U00096.2). Column Start denotes start of the binding site. End denotes the downstream start codon. Gene denotes the name of the first gene in the corresponding mRNA. Length denotes the length of the UTR.

**Table 11:** **Riboswitch statistics**

**Total**	**L**	**MFE**	**GC**	**std(L)**	**std(MFE)**	**std(GC)**
Sense	117.04	-42.63	0.51	47.81	21.54	0.09
Antisense	117.04	-37.73	0.51	47.81	19.55	0.09
UTR	49.53	-6.48	0.37	19.37	5.77	0.08
**Train**	**L**	**MFE**	**GC**	**std(L)**	**std(MFE)**	**std(GC)**
Sense	114.1	-41.05	0.52	49.27	23.83	0.1
Antisense	114.1	-37.73	0.52	49.27	21.32	0.1
UTR	48.18	-5.4	0.35	18.27	5.1	0.08
**Test**	**L**	**MFE**	**GC**	**std(L)**	**std(MFE)**	**std(GC)**
Sense	120.74	-44.63	0.49	46.71	18.6	0.09
Antisense	120.74	-37.74	0.49	46.71	17.54	0.09
UTR	51.31	-7.9	0.39	21.35	6.47	0.08

Average and standard deviation values of Length, MFE, and GC-compositions of the training and test sets. Column Sense denotes riboswitches. Column UTR denotes *E. coli* UTR sequences collected.

The performance of the tri-state classifier was evaluated by estimating classifier parameters from multinomial logistic regression of the training sets and then calculating the correct classification of sequences having zero (*E. coli*) riboswitch structure, one possible structure (antisense), or two (riboswitch) structures that are in the test set. We also evaluated the performance of classifiers that incorporate features from FFTbor and RNAShapes software packages calculated according to Methods section. Feature sets {*L,MFE,GC,FFTbor*} denoted as LMFEGCFFTbor and {*L,MFE,GC,RNAShapes*} denoted as LMFEGCShapes were included in the test.

Classification performance values are denoted in Table 12 along with sensitivity of each classifier. Sensitivity of tri-state classifiers were defined here as total number of correctly classified sequences divided by total number of sequences classified. Model LMFEGCBJK resulted in both highest sensitivity (80.2*%*) and highest percentage of correctly classified riboswitches (91.3*%*). Performance of other classifiers was in the same range. Further tests are needed to make better comparison between the performance of the classifiers. The low performance of the LMFEGCRND model shows that classification is potentially sensitive to features length, GC-composition, and MFE, since they are different between the training and the test sets (see Table 11). Furthermore, choice of modeling is very critical in designing sense-antisense classification. The BJK model, being a more accurate folding model leads to higher performance.

**Table 12:** **Classification performance**

**Classifier**	***TP%***	***F*** ***P***_**1**_ ***%***	***F*** ***P***_**2**_ ***%***	**Sensitivity**	**Sig.**
LMFEGCBJK	91.3	43.5	15.4	72.9	MFE
LMFEGC	82.6	30.4	23.1	71.2	MFE
LMFEGCRND	73.9	30.4	38.5	64.4	L,MFE
LMFEGCFFTbor	82.6	30.4	23.1	71.2	-
LMFEGCShapes	87.0	34.8	23.1	71.2	-

Classifier Performance. Actual length of sequences used. Column Classifier denotes features used from the training set. *T* *P* *%* denotes percentage of true positives. *F* *P*_1_ *%* and *F* *P*_2_ *%* represent the percentages of antisense sequences and *E. coli* UTRs that are misclassified as riboswitches, respectively. Sensitivity denotes overall percentage of correctly classified sequences. Sig. denotes significant (less than 0.05 in the training set) features of the multinomial classifier.

**Figure 4:**
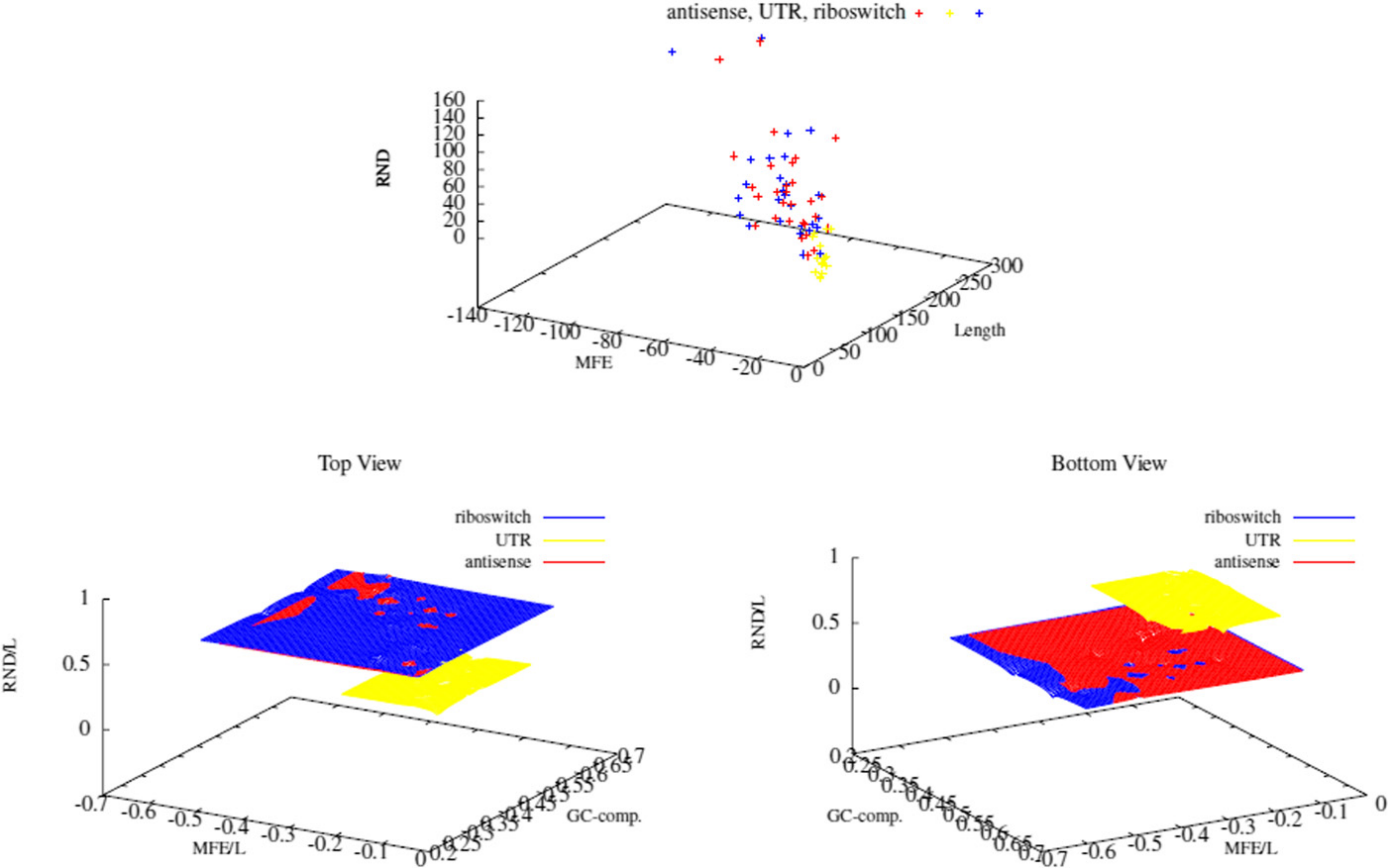
Structural Entropy vs. GC-comp. and MFE. 3D-plot of features MFE, Length and Structural Entropy of the training set sequences classifier under the RND model. Grid-view of different sets of sequences are shown in the top and Bottom views, riboswitches, *E. coli* UTRs, and antisense sequences. Axes *R* *N* *D*/*L* and *M* *F* *E*/*L* show Structural Entropy and MFE normalized by the length of the sequence, respectively. Euclidean distance to actual values was used to generate the grids.

Regression coefficients of the classifiers corresponding to riboswitches are shown in Column *β*_2_ of Table 13. Coefficients corresponding to MFE and structural entropy, are the second and forth values, respectively. For both the LMFEGCRND and LMFEGCBJK models, MFE coefficients are negative while structural entropy coefficients are positive (values are normalized with respect to antisense). This implies that if we input a riboswitch and its reverse complement to the regression-based classifiers, the strand with lower Minimum Free Energy and higher structural entropy is more likely to be the riboswitch. Hence, we have reason to believe that despite having a more stable structure, riboswitches tend to have higher structural entropy than expected^b^. We find this observation significant, since they are consistent across two different folding models. 3D-plots of the MFE, GC-composition, and structural entropy values under the RND model for sequences of the training set are depicted in Figure 4. Top and bottom views of the grid-view of values normalized to sequence length roughly shows this distinction.

**Table 13:** **Logistic regression coefficients of classifiers**

**Classifier**	***β***_**1**_	***β***_**2**_
LMFEGCRND	3.191,.336,.683,-.723,-.465	5.052,-.161,-.089,-7.454,.220
LMFEGCBJK	10.597,-.203,.367,-10.856,.651	5.524,-.082,-.132,-9.247,.120
LMFEGC	3.869,.052,.525,-1.419	3.373,-.025,-.068,-6.234

Regression coefficients (exponents) of the multinomial logistic regression classifier: intercept, Length, MFE, GC-composition, Entropy. Parameter vectors *β*_1_ and *β*_2_ denote coefficients for *E. coli* UTRs and ribswitch sense sequences for the riboswitches of the training set, respectively. Coefficients normalized with respect to those for riboswitch antisenses. i. e. antisense coeficients being 0.

Testing the classifiers on constant lengths of sequences (for all training and test sets) did not increase performance (see Table 14 in Appendix), although the *RND* was significant for sequences of length 150 nt in the training set. Constant length selection was based on extending (or shortening) the original choice of length of sequences from both 5’ and 3’ directions such that the center of the sequence does not change. We refer to this original choice of length as the actual length, hereon. We chose this scheme for simplicity. Other sequence selection methods may be preferred, since the alternative fold may occur on varying parts (5’ or 3’) of the riboswitch sequence, in general. Substitution of CFE feature instead of MFE feature resulted in lower performance of classifiers (comparing Tables 10, 11, 12, 13, 14 and 15 in Appendix).

**Table 14:** **Classification performance for different choices of length**

**Variable Length**					
**Features**	***T*** ***P*** ***%***	***F*** ***P***_**1**_ ***%***	***F*** ***P***_**2**_ ***%***	**Sensitivity**	**Sig.**
L,MFE,GC,RND	69.6	39.1	7.7	61	MFE,GC
L,MFE,GC,BJK	87.0	34.8	0.0	71.2	GC
L,MFE,GC	87.0	30.4	0.0	76.3	L,MFE
**100**					
**Features**	***TP%***	***FP***_***1***_ ***%***	***FP***_***2***_ ***%***	**Sensitivity**	**Sig.**
MFE,GC,RND	69.6	26.1	7.7	66.1	-
MFE,GC,BJK	65.2	21.7	7.7	64.4	-
MFE,GC	56.5	21.7	15.4	64.4	-
**150**					
**Features**	***TP%***	***FP***_***1***_ ***%***	***FP***_***2***_ ***%***	**Sensitivity**	**Sig.**
MFE,GC,RND	69.6	26.1	23.1	57.6	MFE,RND
MFE,GC,BJK	69.6	39.1	7.7	59.3	MFE
MFE,GC	69.6	39.1	0.0	64.4	-
**200**					
**Features**	***TP%***	***FP***_***1***_ ***%***	***FP***_***2***_ ***%***	**Sensitivity**	**Sig.**
MFE,GC,RND	65.2	34.8	7.7	62.7	MFE
MFE,GC,BJK	78.3	34.8	7.7	66.1	MFE
MFE,GC	82.6	39.1	0.0	76.3	MFE

Sub-section Variable Length refers to results of actual sequence lengths for both training and test sets (equal number of varying sequence lengths of 100, 150, and 200 from *E. coli* UTR chosen as negative set). Sub-sections 100, 150, and 200 refers to results where all sequences in the training and test sets have a constant length. Column Features denotes features used from the training set. *T* *P* *%* denotes percentage of true positives. *F* *P*_1_ *%* and *F* *P*_2_ *%* represent the percentages of antisense sequences and *E. coli* UTRs that are misclassified as riboswitches, respectively. Sensitivity denotes overall percentage of correctly classified sequences. Sig. denotes significant (less than 0.05 in the training set) features of the multinomial classifier.

**Table 15:** **Classification performance using centroid free energy**

**Classifier**	***T*** ***P*** ***%***	***F*** ***P***_**1**_ ***%***	***F*** ***P***_**2**_ ***%***	**Sensitivity**	**Sig.**
LCFEGCRND	65.2	30.4	15.4	66.1	CFE
LCFEGC	78.3	56.5	15.4	61	L,CFE
LCFEGCBJK	82.6	65.2	15.4	59.3	GC

Classifier Performance. Actual length of sequences used. Feature CFE denotes centroid free energy as calculated by CentroidFoldⒸ [62]. Column Classifier denotes features used from the training set. *T* *P* *%* denotes percentage of true positives. *F* *P*_1_ *%* and *F* *P*_2_ *%* represent the percentages of antisense sequences and *E. coli* UTRs that are misclassified as riboswitches, respectively. Sensitivity denotes overall percentage of correctly classified sequences. Sig. denotes significant (less than 0.05 in the training set) features of the multinomial classifier.

### Association with high entropy

Mutagenesis results suggested an association between alternative folds and higher structural entropy. Furthermore, regression approaches to estimate the structural entropy of the riboswitch with respect to various sequence and structural features such as MFE lead to higher classification performance in discriminating riboswitches from their antisense control, compared to classifiers that do not incorporate the structural entropy measure. Similar to Mutagenesis results, we observed that riboswitches tend to have higher structural entropy than what is expected of their antisense sequences. Dinucleotide shuffles test also showed an slight increase in the specificity of classifiers using one of the structural entropy features (The RND model) compared to other models and methods. We hypothesize that the structural entropy value of riboswitches may be a significant factor within the context of their length, GC-composition, and folding stability (here, MFE). In specific, conformational switches (here riboswitches) show a slight but consistent increase in their entropy values than structural mutants or antisense. The higher entropy was not, however, observed in the dinucleotide shuffling test. Under this hypothesis and the limitations of our tests from both mutagenesis and sense-antisense classification, the *B. subtilis* Magnesium and SAM-I riboswitches seem to be two immediate outliers.

Base-pairing entropy feature had higher performance in the Mutagenesis results, while the energy landscape index led to higher performance in sense-antise classification. Putting Mutagenesis and sense-antisense results in one perspective, however, suggests a more consistent conclusion about the structural entropy compared to the other two features.

Some of the challenges in our approach to develop riboswitch identifiers were choices of sequence segment and folding model. We found it very difficult to find a subset of sequence segments from riboswitches for our training set that had the highest structural entropy. These difficulties included but were not limited to high sensitivity of structural entropy to sequence length and location and the possible varying lengths of riboswitches that have alternative structures. We arbitrarily included varying lengths of riboswitches in our training set rather than constant length, since the performance of classifiers with constant length was either lower or similar to those with varying length. Methods based on optimization of both sequence length of riboswitches and modeling their folding dynamics may prove rewarding in this regard.

The optimum length of a sequence segment that leads to identifying riboswitches can vary from one organism to another; Constant length of 100 nt segments for *E. coli* are more suitable, while 157 nt segments lead to higher performance for *B. subtilis* riboswitches. Results about sequence segments, however, had low significance due to low number of riboswitches tested in each case. We only propose that it may be possible that riboswitches from different organisms may have different ranges of sequence lengths over which alternative structure prediction becomes significant. Optimizing search parameters on a new organism sequence is potentially a difficult task. One alternative may be evaluating the behavior of structural entropy-based classifiers on data sets that are peculiar to that organism. We have not explored this approach.

### Choice of model

Classification performance of sense-antisense, genome-wide sliding window tests, and mutagenesis all suggest that the BJK folding model is more sensitive to changes in the folding space than the structurally ambiguous RND model. The classification performance of the LMFEGCBJK model both on the test set and on the *B. subtilis* riboswitches is high given the right sequence segment is chosen. Also, the RND model does very poorly in distinguishing the folding space of riboswitch mutants from that of their wild types. On the other hand, binomial logistic regression based classification of sense and antisense of all riboswitches assigns slightly higher ROC area to the classifier that deploys the RND model (see Figure 2). Furthermore, riboswitch identifiers based on the RND model are more robust in terms of sequence positioning than their BJK counterparts. The RND model only enforces Watson-Crick and G-U base-pairing and is fairly a simplistic structural model. The acceptable performance of the RND model in genome-wide approaches may be due to having less structural constraints than BJK. It may be possible that training secondary structural folding models to predict RNA secondary structures comes at the cost of loss in folding dynamics information. Overall, design of a more efficient SCFG model (possibly a heavyweight folding model) to characterize better the riboswitch folding space is another bottleneck of this approach, since modeling techniques to capture conformational features have not been developed. Current RNA structure modeling is centered around prdiction of the RNA secondary structure rather than its conformational dynamics.

### Genome-wide analysis

Sequence segments predicted to have potential for alternative fold for the two *B. subtilis* and *E. coli* intergenic regions are presented in Genome-wide scan results. The power of regression based classifiers as riboswitch predictors is not significantly high; None of the known *B. subtilis* riboswitches fell in the top 100 hits in genomic scans, though certain of those hits are known regulatory elements. The lack of high performance implies sensitivity of our approach to training set genomic features. Exploring other classification schemes, such as neural networks (for instance, similar to [63]) as well as incorporating a different negative-control training data than the antisense may lead to higher performance. In order to develop an organism-specific riboswitch predictor, one may gather sequences with no structure from the target organism and deploy it as a negative control for a classifier that takes structural entropy as a feature.

Many hits fell immediately upstream of operons, which could be indicative of *cis*-regulation. Our genome-wide scan results show dependency of genomic features such as the uracil composition. Furthermore, results presented for various genome-wide scans cannot be taken into account individually, since the above methodology is a length based method. In other words, in order to identify genomic regions with highest likelihood of having a riboswitch, it is essential to combine results of genome-wide scans under different window sizes. Such combining of results also seems organism-specific. The optimum length(s) of genome-wide window scans for the riboswitch identification can be different from one organism to another. The complexity of such a test and its corresponding rigorous statistical analyses fell outside the scope of this work. Here, we limited ourselves to few genomic scans and present a subset of regions that are riboswitch candidates in most of the genome-wide tests.

#### The *cotH* gene

The top two sequence segments predicted to be riboswitches are both upstream of *cotH* gene and in close proximity of one another. In fact, a 628 nt long segment is classified to be a riboswitch (four consecutive sequence segments). The 5’ half of this segment, {3717412 nt - 3717725 nt}, contains the top two hits which are also predicted to be riboswitches by the model LMFEGCBJK in position {3717098 nt - 3717725 nt} on the complementary strand of *B. subtilis*. Naclerio et al., 1996 [64] discuss possible regulation in the vicinity of *cotH* gene. They also stated that no homology to this gene was revealed in the sequences presented in the data bank at the time. They hypothesized that this gene plays an important role in the formation of the spore coat. A more recent paper [65] reports about the *cotH* promoter mapping 812 bp upstream of beginning of its coding region. This region covers the top two hits we have. In fact, a 200 nt scan reveals that many consecutive segments belonging to this region have significant RND entropy values (<0.05). Most interestingly, the segment with highest RND entropy value on a genome-wide level and under the 200 nt window occurs 399 nt upstream of the *cotH* gene at location {3717398-3717597 +}. The authors also talk about *cotG* and *cotH* genes and that they are both divergently transcribed by *σ*-K and a potential for extensive secondary RNA structures in this unusually long untranslated region. The *cotG* is located in the forward strand. There are also many hits around 2000 nt upstream of the *cotG*-containing operon under various sliding window tests. An interesting observation about the nucleotide composition of the top hit reveals that it uniquely contains periodic runs of 6 consecutive thymines with periodicity of 12 and 15 interchangeably. A search for similar runs of thymines was done on both strands of *B. subtilis* by relaxing periodicity from 10 nt to 18 nt and constraining it to having at least six consecutive runs of 6-thymines using the pattern locator software [66]. The only two hits were found on the reverse strand and overlapping with the top hit: {3717502-3717606} and {3717367-3717468}.

The most significant structural entropy value for the longest window size (200 nt) on the *B. subtilis* genome occurred in an unusually extensive secondary structure within that genome. It may be possible that longer RNA structures contain segments having significantly high entropy (structural entropy) on a genome-wide scale. This implies that longer RNAs could potentially have a uniquely high number of secondary structural conformations. This unusually high secondary structural diversity may be related to their regulatory role. We have not yet examined the secondary structural space of other long secondary structures in various organisms. The significantly high structural entropy feature, however, may be typical of other longer secondary strucures. In a recent study on the newly discovered class of RNAs known as long ncRNAs (lncRNA), Cloutier et al. [67] show that yeast lncRNAs are involved in the timing of gene expression. It may be possible that their proposed lncRNA-dependent *quick shift* of gene expression is related to a high potential for diverse secondary structural conformations.

#### The BSU tRNA 75 operon

The sequence segment with highest classification probability that also has signficant MFE and structural entropy values is located about 2277 nt of the upstream region of the BSU tRNA 75 Operon. The antisense control of this segment is located in a putative transcriptional regulator. It is interesting, however, that this hit occurs upstream of a tRNA operon. A 200 nt scan reveals more hits upstream of this operon that have significant structural entropy values some of which are closer to the tRNA operon (around 2000 nt upstream). From locations of tRNA operon [68], it can be seen that out of the five consecutive tRNA genes with isotypes Glu, Val, Thr, Tyr, and Gln, the Thr operon has attenuation [69]. Although the long distance from the downstream translation start codon does not make this a reliable riboswitch prediction, the significance of hits in the intergenic region upstream of the Thr gene and the fact that the other top hit in our classification approach was located in a long RNA, suggest the possibility that there may be a long regulatory RNA residing upstream of the mentioned tRNA operon, raising the interesting possibility of a putative riboswitch regulating an attenuation mechanism. Another possiblity is the existence of an open reading frame downstream of the hit. We applied GeneMark [70] and GLIMMER [71,72] gene prediction programs on the 2000 nt long downstream sequence of this hit. The *Bacillus anthracis* gene model was used in GeneMark, since *B. subtilis* was not available. Both programs had similar gene prediction results, showing the possibility of three open reading frames on the complementary strand, with the closest one ending 46 nt downstream of the hit.

#### lysP

One of the most significant hits in our classification under the 157 nt scan occurs immediately upstream of the *lysP* gene. The segment corresponding to this location also has the most significant (highest) RND entropy value while having significantly low MFE (p-Val. <0.05) on a genome-wide level. This is also true for the 150 nt window scan. Furthermore, the 200 nt scan assigns significantly high structural entropy (RND p-Val. <0.05) as well as classification probability of higher than 0.8 for this location. The 150 nt-long segment is located at {3421066-3421215 -} between the lysine permease and BSU MISC RNA 54. Other adjacent hits that overlap BSU MISC RNA 54 do not have such high structural entropy or classification probability. It may be possible that this segment plays a crucial role in regulating the downstream gene.

### Cross-organism riboswitch candidates

The *B. subtilis* (BSU16090) had a homologue in the *E. coli* operon: (sdhA, sdhB, sdhC, sdhD, sucA, sucB, sucC, sucD), that contains the sucC as its last gene, with e-value 3e-141 (see Methods section for details). The genes were associated with top hits on their upstream in *B. subtilis* and *E. coli* with probabilities 0.897 and 0.905, respectively. Also, the *B. subtilis* sucA (BSU19370) gene had a homologue in the *E. coli* fixA,fixB,fixC,fix operon with e-value 1e-18, both of which were associated with high hits. Other matches were the *B. subtilis* tagA gene and the *E. coli* (rfe- wzzE- rffEDGHCA- wzxE- rffT- wzyE- rffM) operon (e-value 1e-23), the *B. subtilis* citB and the *E. coli* acnA genes (e-value 0), the *B. subtilis* cspB and the *E. coli* cspG genes (e-value 2e-17), and the *B. subtilis* ydaB gene and the *E. coli* caiA,caiB,caiC,caiD,caiE,caiT operon (e-value 3e-27). None of the above *B. subtilis* genes, however, had homologs in *S. elongates* that was associated with top hits in that organism, except for the *B. subtilis* ydaB gene. The ydaB gene had a homologue in *S. elongates* syc0203_c (e-value 7e-13) where the associated upstream region contained a hit with probability 0.714. Other homologues were also observed between *B. subtilis* and *E. coli*. However, the upstream information for those hits was not available, since they either had intergenic regions shorter than 150 nt or they contained annotated regulatory elements which were excluded from the scanning procedure.

## Conclusion

Riboswitches are comprised of a diversity of biological functionality, as well as having different conformational dynamics. In this work, we made an attempt to characterize the potential for an alternative fold (switching ability) ubiquitous in various regulatory elements, regardless of their annotation and structural complexities. Various tests showed that there is in fact a relationship between higher structural entropy and RNA switching ability. This relationship was shown to be modest but consistent across various tests. Furthermore, incorporating the structural entropy feature under the simplistic and symmetric RND folding model was shown to be informative in distinguishing riboswitches from their random shuffles. Unlike results from mutagenesis and antisense tests, the structural entropy feature of riboswitches was not on average higher than their corresponding random shuffles. Given both the diversity of conformational arrangement of gathered riboswitches and the simplicity of models used, there is potential for this feature in detecting RNA conformational switching and ultimately in *de novo* riboswitch discovery.

Classifiers based on structural entropy optimized through sequence and structural features were devised to distinguish between the putative riboswitch and the antisense control. They were also used as riboswitch identifiers in various prokaryotes. Potential shortcomings and considerations were also explored. Factors such as, length, organism the riboswitch is taken from, and the type of riboswitch should be considered in preparing a training set for future approaches. The lightweight RND folding model tended to have a very consistent and robust result in distinguishing extensive secondary structures from other intergenic regions on genome-wide scale, regardless of test parameters.

Structural entropy using stochastic context-free grammars provides a means to better understanding the conformational dynamics of the RNA, in general. Current modeling training techniques for SCFGs focus on higher accuracy to predict RNA secondary structure, and not necessarily higher accuracy of folding space or dynamics. Interestingly, the more simplistic RND folding model used in our approach had a higher performance than the more accurate BJK model, under serveral tests. The use of a more complex and accurate folding model may not necessarily result in a better exploration of the folding dynamics of non-coding RNAs. Fully understanding the behaviour of structural entropy may require extensive exploratory investigation, both from modeling and data collection perspectives.

## Methods

### Data collection

Sequences with concrete evidence of alternative structures were gathered from the literature (see Table 2). Prokaryotic sequences believed not to have structure were selected from *E. coli* and are listed in Table 9 as negative set. 30 genome locations corresponding to *σ*-70 transcription factor binding sites that are less than 80 nt upstream of their corresponding start codon were randomly chosen from *E. coli* such that they are fairly evenly distributed across the genome. Data was manually gathered from the EcoCyc website (http://ecocyc.org/).

### Mutagenesis

Sensitivity and specificity values of Table 5 were calculated from Table 4. Sensitivity: the percentage of structural mutants (annotated by YES in Table 4) having lower values than the wild type (corresponding negative value in Table 4). Specificity: the percentage of non-structural mutants (annotated by NO in Table 4) having higher or equal values with respect to the wild type (corresponding positive or zero value in Table 4). Mathews Correlation Coefficient is shown in column MCC. Features Base-pairing entropy (BJKbp), Structural entropy under BJK and RND models, (BJK) and (RND), respectively, and two-cluster average silhouette index of energy landscape (Sil) were investigated. For the case of the *Bradyrhizobium* ROSE-P2, entropy values were compared with −0.74 rather than zero for wild type, since the length of the 135nt-long riboswitch was decreased by 1 and this decrease in length is expected to have linear effect on structural entropy values.

### Classification

#### Preparing the positive control set

The criterion for building the positive control set was taking the minimum-length sub-sequence for the corresponding riboswitch with evidence for alternative structures. Comprehensive structure information was not available for certain sequences. We decided to include them to increase our data set size. The structures of most sequences were experimentally validated, although a few structures of the riboswitches were inferred in combination with structural homology approaches. Only the expression platform components for the Cobalamin riboswitches were used, since they contain alterations; a typical riboswitch has an aptamer and an expression platform component, where the aptamer binds to the ligand, triggering allosteric rearrangement of the conformation of the expression platform component of the riboswitch which in turn regulates the expression of the downstream gene. Cobalamin riboswitches are also significantly longer than other sequences, e.g. *Salmonella enterica serovar Typhimurium*’s Cobalamin riboswitch was over 300 nt long. Including such long sequences could have been problematic, for both sensitivity of structural entropy on sequence length and the fact that RNA structures longer than 200 nt are usually predicted with low confidence under SCFG models as well as computational constraints. Also, certain sequences were excluded from the test. In the column Grouping of Table 2 we denote None for such sequences. Excluded sequences are as follows: *Salmonella* ATP regulatory element, located in the *mgtM* gene before the *mgtCBR* operon, was excluded since it was the only RNA in our set that had complete overlap with codons [73]. *Synechococcus sp. CC9902* Downstream-peptide motif was excluded, since evidence for alteration was not available. *T. tengcongensis* glmS ribozyme-riboswitch was excluded, since the glmS ribozyme does not undergo “large conformational changes concomitant with ligand binding” [74] and is the only RNA element in our gathered data that functions as a self-cleaving ribozyme upon binding to glucosamine-6-phosphate (GlcN6P) ligand [75]. *Synechococcus elongatus* glnA motif was excluded, since no evidence of alteration was available. *Schistosoma Mansoni* Hammerhead type I ribozyme was excluded, since its structure does not alter. The pseudoknotted *marine metagenome* Hammerhead type II ribozyme was also excluded, since there was no evidence of alteration of the secondary structure. Finally, *Bacillus subtilis* yxkD motif was excluded, since there was no concrete biological evidence for it being a functional riboswitch or ribo-regulator, although it is predicted partially to have an alternative structure [76].

#### Choice of reverse-complement (Antisense) as a negative control

As we know, riboswitches are under selective pressure to preserve their potential for alternative folds due to their biological role. The reverse-complement of the RNA or the antisense sequence was here assumed not to have potential for two alternative structures; they may have at most one structure, since they are complementary to the sense. A *cis*-regulator has an alternative fold, typically through conformational rearrangements of the expression platform to be able to regulate the expression of the downstream genes in the same mRNA, while the antisense is not necessarily under such evolutionary pressure (See Background section for exceptions). Experimental work to back this assumption on the gathered riboswitches was not found. The following assumption was made: On average, a given set of validated riboswitch sequences are expected to contain more RNA switching features than their corresponding antisense sequences. Advantages and disadvantages of the choice of reverse complement (or antisense) are as follows: Advantages: In the reverse complement, Watson-Crick pairing positions are kept intact. This implies that the folding space of the antisense may contain secondary structural features similar to the MFE structure of the sense sequence, making it a near negative control. Other more established negative controls such as various sequence shuffling techniques do not guarantee this. In addition, high correlation of structural entropy values to nucleotide composition and length of the sequence [55] make antisense a convenient choice of negative control. Disadvantages: There may be possible co-association with other sequence features such as U-composition. Also, G-U pairs may differ between sense and antisense structures.

#### Training and test sets

The positive control set was divided into the training and test sets. While most gathered sequences were in the two organisms, *B. subtilis* and *E. coli*, they cover a variety of biological functions and structures. We were interested in a method that can identify potential for the RNA to have an alternative secondary structure from a thermodynamic perspective regardless of a specific function or a secondary structural conformation. Hence, the categorization was done such that each of the training and test sets would contain as many adiverse sequences and structures as possible. Furthermore, the training sequences contain those from *E. coli* while the test set contains those of *B. subtilis*. Riboswitches that did not exist in both gram-positive and gram-negatives were evenly distributed between the two sets. Division of data into training and test sets was a compromise between having as diverse riboswitches as possible and being able to assess significance of classification on riboswitches from phylogenetically distant organisms, namely the gram-positive *B. subtilis* and the gram-negative *E. coli*. In the column Grouping of Table 2, the categorization of each sequence is shown. There are a total of 29 sequences in the training set and 23 sequences, in the test set. The 30 *E. coli* UTRs were divided into sets of 17 and 13 for training and test sets, respectively. The categorization was selected such that for an extension of 100 nt UTRs upstream of their corresponding start codons, GC-composition, and the minimum free energy having similar distribution in both sets. A further internal control was the reverse-complement (or antisense) sequence of the riboswitch, adding additional sets of sequences of size 29 and 23 sequence to the training and test sets, respectively. Various classifications in this work always use antisense sequences of riboswitches of identical length for training and test sets, unless indicated otherwise. For the dinucleotide shuffling test, we originally generated 10 dinucleotide shuffles for each riboswitch using [60]. We then filtered the sequences and selected those having more similar MFE values to riboswitches based on the following criterion. For each of the training and test sets, we calculated average and standard deviation of the MFE values. We then eliminated those random shuffles having higher MFE values than average minus one standard deviation, for the training and test sets separately. The resulting statistics can be seen in Table 8. As we can see, the statistics for other features such as length and GC-composition were also altered.

#### Classification criterion

Classification probabilities of having an alternative fold (riboswitch), possibly only one fold (antisense), or no riboswitch structure (*E. coli* UTR) were assigned to each sequence based on multinomial logistic regression of sequences in the training. SPSS 16.0Ⓒsoftware was used to estimate the corresponding parameter vectors. Such parameter vectors were then used to calculate the probabilities of sequences in the test set to belong to each class using their features. All true and false positive rates presented (except the ROC curves) are based on maximum likelihood, where the probability with maximum value determines the class of the sequence in the test set. In this work and all presented tables, the term *probability* (or likelihood score) for being potential riboswitches refers to “trained output logistic model score” assigned to the sequence. Entropy calculations were done according to [55]. Two different lightweight context-free secondary structural models were used as folding distribution models. The first model, denoted here as BJK, was developed by Knudsen/Hein and originally used in the Pfold package [77,78]. The structurally unambiguous grammar was subsequently used in [79] under the name G6 to predict RNA secondary structure using different training sets for RNA secondary structures. Model parameters used here correspond to the benchmark-trained version of this grammar [79] and will be referred to as the BJK model. Average sensitivity and specificity values for the BJK model on the test set of riboswitches are 75.6 and 76.3, respectively. The second model, denoted here as RND was introduced in [55] under the name RND10. This model consists of a structurally ambiguous simple grammar with symmetric rules and probabilities set according to [55]. Also, an effort was done to convert non-stacking heavyweight grammars from [13,59]. Such grammars aim at mirroring the state-of-the-art thermodynamic folding models and are extremely sophisticated, requiring their specific software implementation. The translation of these models into our simple implementation eliminated many of its features. The resulting model did not yield the original accuracy to predict RNA secondary structure, nor was its entropy showing any significant performance in the classifier (data not shown). Minimum-free-energy calculation was done by ViennaⒸSoftware Package [58] using default parameters. Base-pairing entropy for the BJK model, denoted here as BJKbp, was calculated as defined in [54] (implementation by [55]). BJKbp calculation is according to ([61] Eq. 3), where natural logarithm is used for base-pairing entropy calculations: $-(1/n)\times \sum _{i < j} P_{i,j}\times \log P_{i,j}$. Symbol *n* denotes length of the RNA sequence and *P*_*i*,*j*_ denotes the probability of pairing in positions *i* and *j*, under the given SCFG model. The two-cluster average Silhouette index of energy landscape, denoted here as *Sil*, was calculated according to the pipeline used in [46] with the exception that we did not account for pseudoknots and only used MFE predictions of the ViennaⒸSoftware Package for prediction of structures. Also, in the case of random shuffles, we only used the fixed number of 500 structural samples to do the calculations. RNAShapes [49] was used to derive an array of most possible abstract shapes resembling RNA secondary structure. We used parameters -e [-500,10] -p to calculate different conformation probabilities. we used the ratio of the two probabilities *p*1/*p*2, where *p*1 and *p*2 are the highest and the second highest probabilities associated with the predictions. One sequence lead to only one possible conformation as an output, for which we arbitrary chose 100. A similar trend was followed using FFTbor [52] software package.

We also tried to explore GC composition information upstream of gathered sequences relative to that in the riboswitch which did not lead to significantly better results. Sequence-similarity method such, as BLASTⒸ and profile Hidden Markov Models were also examined as classifiers with the mentioned training and test sets. The pipeline was implemented according to [80]. These methods did not result in significant classification performance even after lowering the corresponding threshold to insignificant values.

### Genome-wide scan of the *B. subtilis*, *E. coli*, and *S. elongatus* intergenic regions

*Bacillus subtilis subsp. subtilis str. 168* (taxid:224308), *Escherichia coli str. K-12 substr. MG1655* (GenBankⒸID: U00096.2) and *Synechococcus elongatus PCC 6301* were downloaded from the National Center for Biotechnology Information (NCBI)Ⓒ [81,82]. The newer version of *E. coli* str. K-12 genome (gb|U00096.3) was not used, since operon and *σ*-70 UTR locations were given in the old version. Corresponding locations of *E. coli* riboswitches in the old version were used, where necessary. The operon-location information file for *B. subtilis* was downloaded from [83]. Candidates consisted of sequence segments of lengths 100 nt, 150 nt, and 157 nt. Each intergenic region was divided into segments of such length such that the most downstream segment in each intergenic region ends at the start codon. Only intergenic regions higher than 150 nt were considered. True positives were defined based on sequence segments that had maximum overlap with the original coordinates of the riboswitches. The same process was applied to the *E. coli* genome. Operon locations for the *E. coli* genome were downloaded from the RegulonDB website [84]. Operon locations in *E. coli* also contained RNA elements in our data set. Hence, results for the genome-wide scan of *E. coli* did not contain any sequence within the operon locations and only contained non annotated regions. *S. elongatus* gene locations were downloaded from the MicrobesOnline Operon Predictions website [85,86]. *S. elongatus* intergenic regions were chosen in a similar fashion. Computational complexity of the genome-wide scan on the available cluster and using parallel computation took roughly several days for a window size of 200 nt and overlap of 100 nt.

### Cross-organism riboswitch candidate selection

We specifically looked for homologous genes that have riboswitch candidates in their upstream region in different organisms. First, from the top 100 hits in *B. subtilis*, we collected the genes that are associated with hits having maximum distance of 600 nt to the start codon. We then used tblastx to find their homologues in *E. coli* with an e-value threshold of 1e-6.

## Endnotes

^a^Pseudoknots, another RNA structural feature, are a kind of base-pairings that resemble structural knots and cannot be predicted via context-free grammars. Predictions of pseudoknots based on minimum free energy and context-sensitive grammars are possible, though computationally expensive [87]. ^b^Structural entropy was observed to be positively correlated with the MFE for random computer-generated sequences and structures under the BJK model (the lower the MFE the lower its entropy). While structural entropy under the RND model was observed to be independent of MFE (data not shown). Hence, higher entropy (structural entropy) and lower MFE of riboswitches is an unexpected observation, at least from a random sequence perspective. ^c^Structure partially validated, partially predicted via Vienna Software where not available. ^d^Ten nucleotides added to the structure with no structure. ^e^Partially predicted. ^f^Structural Homology Inferred. ^g^Predicted by pknotsRGⒸprogram [88]. ^h^Structural Homology Inferred. ^i^Predicted by Vienna. ^j^Partially predicted by Vienna.

## Appendix

### Additional tables

Performance of different classifiers was evaluated for both constant and variable choices of length for the riboswitches (Table 14). The performance of classifiers that substitute centroid free energy for minimum free energy is shown in Table 15.

### Collected riboswitch sequences

Riboswitch sequences and their corresponding secondary structures were collected from the literature. Riboswitches used in the training set are shown in Training set in Appendix. Riboswitches used in the test set are shown in Test set in Appendix. Sequences excluded from classification are shown in Excluded set.

#### Training set



### Test set



#### Excluded set



### Genome-wide scan results

#### *Bacillus subtilis*

In order to gain a better insight into the structural entropy of various riboswitches within their genomic context, in an exploratory attempt, we examined both the performance of classifiers and the high entropy (structural entropy) hypothesis on a genome level. In order to assess the performance of the classifier, we used regression values derived from the same training set to assign likelihood of being a riboswtich to various genomic locations of the *B. subtilis* genome. As a departure from classification method, we also searched for sequence segments that support our hypothesis; for that we collected sequence segments having both significantly high structural entropy and low MFE. Finally we explored the high structural entropy hypothesis in an inter-organism test, where we looked for homologous genes across the three distant organisms of *B. subtilis*, *E. coli*, and *S. elongatus*, that are all being associated with significantly high structural entropy values upstream of their coding region.

Eleven of the 23 riboswitches of the test set were also located in *B. subtilis*. The performance of the three tri-state classifiers on the eleven riboswitches and all other intergenic regions of the gram-positive bacterium, *Bacillus subtilis* are shown in Tables 16 and 17 in Appendix, for the actual variable lengths and constant lengths of the test set, respectively. In order to have a broader view of classification performance for the *B. subtilis*, we also replaced the antisense with sequences from intergenic regions, having same length and GC-composition as the sense sequences. The negative set consisted of eleven sequences and denoted here as *F* *P*_3_. Performance values are reported in Table 18 in Appendix.

**Table 16:** **Classification performance in** ***B. subtilis***

**Classifier**	***T*** ***P*** ***%***	***F*** ***P***_**1**_ ***%***
LMFEGCBJK	91.1	54.5
LMFEGC	81.2	36.4
LMFEGCRND	72.7	36.4

Classifier Performance on the eleven *B. subtilis* riboswitches. Actual length of sequences used. Column Features denotes features used from the training set. *T* *P* *%* denotes percentage of true positives. *F* *P*_1_ *%* represent the percentages of antisense sequences that are misclassified as riboswitches.

**Table 17:** **Classification performance for different choices of length in** ***B. subtilis***

**100 nt window**	***T*** ***P*** ***%***	***F*** ***P***_**2**_ ***%***
LMFEGCRND	63.6	29.8
LMFEGCBJK	27.3	15.4
LMFEGC	18.2	9.0
**150 nt window**	***TP%***	***FP***_***2***_ ***%***
LMFEGCRND	72.7	20.5
LMFEGCBJK	63.6	9.6
LMFEGC	45.5	3.2
**157 nt window**	***TP%***	***FP***_***2***_ ***%***
LMFEGCRND	81.8	19.5
LMFEGCBJK	54.5	8.3
LMFEGC	63.6	2.1
**200 nt window**	***TP%***	***FP***_***2***_ ***%***
LMFEGCRND	72.7	14.2
LMFEGCBJK	45.5	6.7
LMFEGC	18.2	1.3

Classifier Performance on the eleven *B. subtilis* riboswitches. Constant length of 100 nt, 150 nt, 157 nt, and 200 nt used for test. *T* *P* *%* denotes percentage of true positives. *F* *P*_2_ *%* represent the overall percentage of sequences that are classified as riboswitches within the *B. subtilis* genome. 50 nt, 75 nt, and 100 nt overlaps were used for for sliding windows of lengths 100 nt, 150 nt, and 200 nt, respectively. No overlaps were used for the 157 nt window. True Positive sequences were according to maximum overlap with the location of the actual length of riboswitches.

**Table 18:** **Classification performance in** ***B. subtilis*** **under 157 nt Length**

**Classifier**	***T*** ***P*** ***%***	***F*** ***P***_**1**_ ***%***	***F*** ***P***_**3**_ ***%***
L,MFE,GC,RND	81.8	19.5	18.2
L,MFE,GC	63.6	2.1	0
L,MFE,GC,BJK	54.5	8.3	18.2

Classifier performance on the eleven riboswitches in *B. subtilis*. Constant length of 157 nt used. Sequence segments chosen according to the scanning procedure. Intergenic regions were divided into non-overlapping 157 nt segments with most downstream segment ending at its corresponding downstream opron. Segments with maximum overlap with riboswitches were chosen as true positives. *T* *P* *%* denotes percentages of correctly classified riboswitches. *F* *P*_1_ *%* denotes percentage of misclassified antisense. *F* *P*_3_ *%* denotes percentage of misclassified negative control segments. Average and standard deviation of the MFE values for the negative control segments are −30.7 and 8.2, respectively.

Operon coordinates were taken from [83]. Performance of classifiers was higher for length 157 nt rather than lengths 100 nt, 150 nt, or 200 nt. This was true even though overlapping sliding windows were used for those lengths (sequence segment with highest overlap was selected as positive hit). In addition, we can see from Table 17 in Appendix that as window size increased, the number of intergenic regions classified as riboswitches (*T* *P*_2_ *%*) decreased. The classification performance of the LMFEGCRND model, however, was maximum at length 157 nt. (Length 157 was found using a rough optimization of various constant-length sequence selection and under the LMFEGCBJK model). We further examined the 157 nt length for two different sets of tests. In the first case, 157 nt-long segments were selected centered at the riboswitch (routine procedure) and in the second case, 157 nt extension of the 5’ start of sequences were chosen. Classification performance is shown in Tables 16 and 19 in Appendix. Performance was very sensitive to positioning of the sequence segment of constant length. For the case of 5’ selections, the LMFEGCBJK model outperformed other models having *T* *P* *%*=90.9 while the centered-segment test had a performance even lower than choosing random positioning. Hence, the LMFEGCBJK is more suitable for high performance where computational complexity is not an issue. For faster genome-wide tests where examining all sequence positions is not possible the LMFEGCRND seemed more appropriate (*T* *P*=81.8*%*) and was based on selection of segments in a non-overlapping fashion, starting at the start codon for each operon. Selecting segments centered at riboswitches resulted in poor performance in *B. subtilis*.

**Table 19:** **Classification performance in** ***B. subtilis*** **under constant length**

**Segment**	**5’**		**Center**	
**Classifier**	***T*** ***P*** ***%***	***F*** ***P***_**1**_ ***%***	***T*** ***P*** ***%***	***F*** ***P***_**1**_ ***%***
L,MFE,GC,BJK	90.9	9.1	63.4	18.2
L,MFE,GC,RND	54.5	0	63.4	36.4
L,MFE,GC	72.7	9.1	63.4	18.2

Classifier performance on the eleven riboswitches in *B. subtilis*. Constant length of 157 nt from 5’ of riboswitch downstream is used for riboswitches (first two columns). Constant length of 157 nt centered at the center of riboswitches used (last two columns). *T* *P* *%* denotes percentages of correctly classified riboswitches. *F* *P*_1_ *%* denotes percentage of misclassified antisense.

The performance of classifiers on the eleven riboswitches were highly dependent on the length and positioning of sequence segments to be tested (see Tables 16, 17 and 19). Furthermore, various riboswitches had different sensitivities to such features (data not shown). We found that sequence segments of length 157 nt resulted in higher performance compared to other lengths tested. Also, without knowledge of the exact location of the riboswitch, the LMFEGCRND model outperformed the LMFEGCBJK model, though the LMFEGCBJK model had a significantly higher performance if sequence segments were positioned at the right locations on the riboswitch. The likelihood for such desired positioning is very low; 1/*W* *L* for each riboswitch, where *WL* is the length of the non-overlapping sliding window.

The ranking of *B. subtilis* riboswitches using their actual length and constant length of 157 nt are shown in Tables 20 and 21, respectively. Classification probability of the LMFEGCRND model corresponding to the sequence segment overlapping with the TPP riboswitch (0.76) was higher than that of other riboswitches with ranking empirical p-value 0.0122. Results for the SAM-I riboswitch, however, were very poor. The actual length of the SAM-I riboswitch used in this study was also 157 nt.

**Table 20:** ***B. subtilis*** **riboswitches ranking under actual-length test**

**Name**	**Probability**
Adenine	0.82
FMN	0.70
TPP	0.68
Tryptophan	0.67
Glycine	0.63
Lysine	0.63
Guanine	0.60
ATP	0.58
Magnesium	0.54
SAM-I	0.53
preQ1	0.451

Ranking probabilities of the eleven *B. subtilis* riboswitches of *B. subtilis* under the LMFEGCBJK classifier. Actual sequence length used as test. Column Probability is the classification probability that the sequence is a riboswitch.

**Table 21:** ***B. subtilis*** **riboswitches ranking under constant-length test**

**Name**	**Overlap**	**Rank**	**p-Value**	**Probability**
TPP	82.9	347	0.0122	0.76
Guanine	90.1	535	0.0189	0.735
ATP	85.6	1159	0.0409	0.676
Lysine	83.5	2278	0.0804	0.612
Adenine	100	2459	0.0868	0.604
FMN	51.7	3880	0.1369	0.547
preQ1	80	4051	0.1429	0.541
Magnesium	62.3	4212	0.1486	0.536
Glycine	91.7	5200	0.1835	0.508
Tryptophan	100	6074	0.2143	0.484
SAM-I	68.8	12330	0.4351	0.356

Ranking probabilities of the eleven *B. subtilis* riboswitches within the 157 nt non-overlaping window scan of the intergenic regions of *B. subtilis* under the LMFEGCRND classifier. Total of 28340 sequence segments belonging to intergenic regions longer than 150 nt were analyzed. Operon coordinates: [83]. Overlap denotes the percentage of overlap of the sequence segment with the riboswitch. Column p-Value is the ranking divided by 28340. Column Probability is the classification probability that the sequence is a riboswitch.

**Figure 5:**
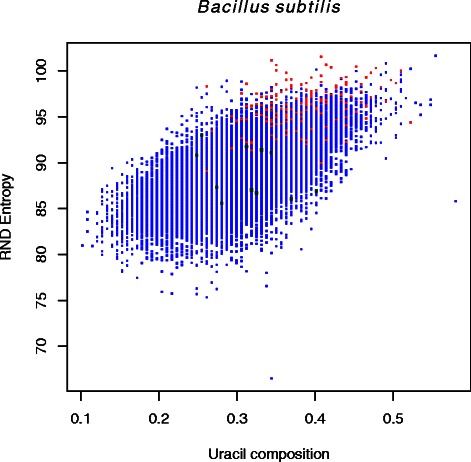
Structural Entropy vs. Uracil-comp. in *B. subtilis*. Entropy Distribution of the 157 nt window-scan results. 28340 candidate segments of *B. subtilis* against uracil composition. Blue denotes all segments. Red denotes those with classification probabilities under the LMFEGCRND are higher than 0.8. Green denotes the eleven bonafide riboswitches of the test set that are in *B. subtilis*.

**Figure 6:**
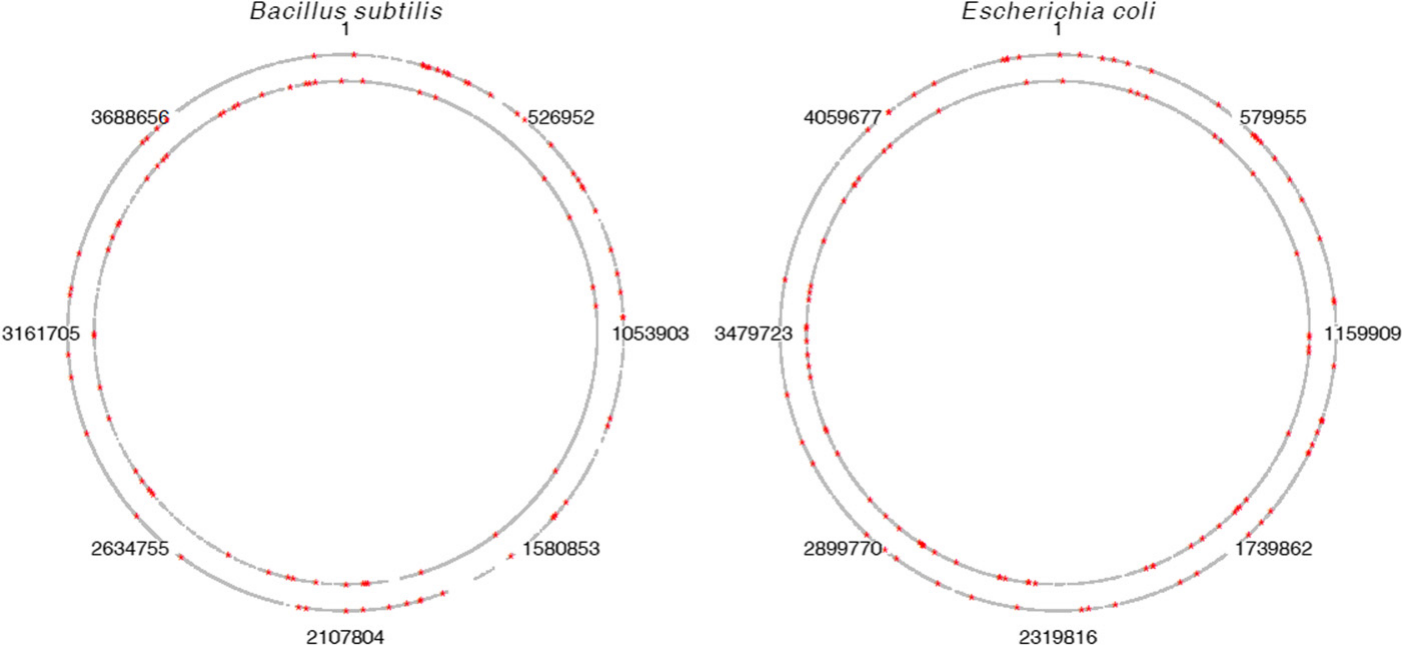
Genomic Distribution. Left: *Bacillus subtilis*. Distribution of locations of sequence segments of the non-overlapping 157 nt window scan of the *B. subtilis* intergenic regions. Location of all segments tested is depicted as grey. Location of segments with uracil composition between 0.2484 and 0.40127 and probabilities higher than 0.8 under the classifier LMFEGCRND are shown in red. Outer circle represents the direct strand while the inner circle represents the complementary strand. 72.2% (39 out of 54) of hits on the forward strand are located in the first half of the genome. 69.6% (32 out of 46) of the hits on the reverse strand are located in the second half of the genome. Right: *Escherichia coli*. Distribution of locations of sequence segments of the 50 nt-overlapping 100 nt window scan of the *E. coli* non-annotated intergenic regions. Location of all segments tested is depicted as grey. Location of segments with uracil composition between 0.23 and 0.34 and probabilities higher than 0.768 under the classifier LMFEGCRND are shown in red (please see Table 28 for more information on the hits). Outer circle represents the direct strand while the inner circle represents the complementary strand. 64.2% (34 out of 53) of hits on the forward strand are located in the first half of the genome. 60% (36 out of 60) of the hits on the reverse strand are located in the second half of the genome.

Table 22 contains the top 50 best hits from each strand of the *B. subtilis* intergenic regions and their corresponding probability values. Sequence segments having classification probabilities higher than or equal to 0.8 fall in the top 50. Plot of structural entropy under the RND model and uracil composition of the sequence segments form the *B. subtilis* showed that structural entropy values were correlated with higher uracil composition (see Figure 5). This may have been partly due to the fact that uracil can bind with more nucleotides to form base-pairs under folding models. In order to suppress the effect of high uracil composition, we sorted the top hits having uracil compositions within the range of known riboswitches in *B. subtilis* in Table 23. The location distribution of these hits can be seen in Figure 6. As we can see, the top hits do not seem to be associated with a specific genomic region as a whole. Sequence segments predicted to be riboswitches were not uniformly distributed across the genome. In order to investigate sequence location of segments with significant structural entropy values, regardless of their regression probabilities, we sorted segments having significantly low MFE (empirical p-value <0.05) while also having structural entropy values on the high 50 percentile. Hits with significant values that had GC and U compositions within the range of known riboswitches in *B. subtilis* are shown in Table 24. Interestingly, all of the hits also had significant structural entropy p-values (<0.05). P-values are calculated empirically and separately for each choice of window size in the genome-wide scan. Finally, significantly high structural entropy values of the 200 nt window scan that also have probability values higher than 0.8, along with other significant hits, are available in Tables 25 and 26 regardless of their MFE or nucleotide compositions.

**Table 22:** **Top classification hits in** ***B. subtilis***

**R**	**Start**	**End**	**Strand**	**Upstream operon**	**Upstream gene**	**Dist. to upstream**	**Uracil**	**Dist. to downstream**	**Downstream gene**	**Downstream operon**	**Probability**
1	3717569	3717725	reverse	BSU36100	ywrD	-1486	0.4076	550	cotH	BSU36060	0.943
2	3717412	3717568	reverse	BSU36100	ywrD	-1643	0.4076	393	cotH	BSU36060	0.935
3	4134175	4134331	reverse	BSU40230	yydA	-182	0.3439	79	yydB	BSU40220	0.931
4	3714883	3715039	forward	BSU36030	ywrK	-859	0.3949	2277	cotG	BSU36070	0.922
5	748990	749146	forward	BSU06780	yeeC	-2912	0.414	707	yeeG	BSU06820	0.919
6	3666640	3666796	reverse	BSU35680	ggaB	-490	0.4968	1335	mnaA	BSU35660	0.908
7	3866327	3866483	reverse	BSU37690	ywfG	-1881	0.3503	79	eutD	BSU37660	0.903
8	681153	681309	forward	BSU06260	ydjN	-201	0.3885	5731	yeaB	BSU06320	0.899
9	2987548	2987704	reverse	BSU29200	accA	-104	0.4268	79	pfkA	BSU29190	0.898
10	1680274	1680430	forward	BSU16080	ylqH	63	0.3822	79	sucC	BSU16090	0.897
11	2730227	2730383	reverse	BSU26730	yrdF	-254	0.4204	79	azlB	BSU26720	0.896
12	2316268	2316424	reverse	BSU22040	ypbQ	-99	0.363	236	ypbR	BSU22030	0.896
13	2219985	2220141	forward	BSU20929	yoyI	-6828	0.4204	2277	yonP	BSU21030	0.896
14	688027	688183	forward	BSU06320	yeaB	-114	0.3885	79	yeaC	BSU06330	0.893
15	243578	243734	forward	BSU02170	ybfB	-5370	0.363	236	purT	BSU02230	0.89
16	984466	984622	reverse	BSU09120	yhcK	-1189	0.3885	79	cspB	BSU09100	0.889
17	2376780	2376936	forward	BSU22510	ypjC	-15199	0.4395	16564	ypzI	BSU22869	0.888
18	748205	748361	forward	BSU06780	yeeC	-2127	0.4395	1492	yeeG	BSU06820	0.886
**19**	3421066	3421222	reverse	BSU33340	sspJ	-320	0.3312	79	lysP	BSU33330	0.885
20	2093235	2093391	forward	BSU19200	desR	-852	0.4331	4789	yoyB	BSU19259	0.88
21	3941212	3941368	reverse	BSU38430	gspA	-3269	0.4777	1649	ywbA	BSU38390	0.879
22	1493630	1493786	forward	BSU14230	ykuV	-230	0.3503	79	rok	BSU14240	0.879
23	2531945	2532101	forward	BSU24210	yqiG	-14308	0.3439	9028	yqhQ	BSU24490	0.879
24	746478	746634	forward	BSU06780	yeeC	-400	0.5095	3219	yeeG	BSU06820	0.878
25	2096100	2096256	reverse	BSU19230	yocJ	-171	0.4268	393	yocI	BSU19220	0.877
26	300673	300829	forward	BSU02770	yccK	-1196	0.3822	79	ycdB	BSU02790	0.875
27	3373963	3374119	reverse	BSU32890	yusQ	-2575	0.4076	393	fadM	BSU32850	0.874
28	3686143	3686299	forward	BSU35770	tagC	-1298	0.4586	2591	gerBA	BSU35800	0.87
29	1335487	1335643	reverse	BSU12820	spoIISB	-12876	0.5032	13895	xre	BSU12510	0.868
30	4139318	4139474	forward	BSU40240	yycS	-3475	0.3567	864	rapG	BSU40300	0.865
31	1268672	1268828	forward	BSU11970	yjcS	-1	0.4458	79	yjdA	BSU11980	0.865
32	3685829	3685985	forward	BSU35770	tagC	-984	0.414	2905	gerBA	BSU35800	0.865
33	3681213	3681369	forward	BSU35670	gtaB	-14627	0.363	79	tagA	BSU35750	0.864
34	1122705	1122861	forward	BSU10490	sipV	55	0.4013	79	yhjG	BSU10500	0.86
35	3671690	3671846	reverse	BSU35700	tagH	-1795	0.3694	393	ggaA	BSU35690	0.859
36	2160701	2160857	forward	BSU20000	yosU	-1938	0.4395	9028	yosA	BSU20190	0.859
37	1097850	1098006	reverse	BSU10230	yhfH	-191	0.465	79	gltT	BSU10220	0.858
38	1467020	1467176	forward	BSU13960	ykwC	-342	0.414	707	pbpH	BSU13980	0.857
**39**	191850	192006	forward	BSU01590	ybaS	-12186	0.3057	2277	trnSL-Glu2	BSU_tRNA_75	0.856
40	20723	20879	forward	BSU00120	yaaE	-86	0.3185	79	serS	BSU00130	0.856
41	2691445	2691601	reverse	BSU26240	yqaO	-1121	0.3439	79	yqaQ	BSU26220	0.852
42	3158851	3159007	reverse	BSU30890	ytxO	-328	0.363	3376	ytdA	BSU30850	0.852
43	1958206	1958362	forward	BSU18190	yngC	-9863	0.3949	44353	iseA	BSU18380	0.852
44	557716	557872	forward	BSU05100	yddT	-188	0.3503	79	ydzN	BSU05109	0.851
45	3907629	3907785	reverse	BSU38100	ywcH	-2594	0.3567	393	ywcI	BSU38080	0.851
46	1926523	1926679	forward	BSU17950	yneJ	-1482	0.3949	79	citB	BSU18000	0.851
47	1017271	1017427	forward	BSU09400	spoVR	-139	0.363	1649	lytE	BSU09420	0.85
48	1493595	1493751	reverse	BSU14250	yknT	-729	0.4522	1649	ykuT	BSU14210	0.85
49	2477743	2477899	forward	BSU23830	yqjL	66	0.4076	1335	zwf	BSU23850	0.849
50	2769617	2769773	reverse	BSU27160	cypB	-4194	0.3185	1021	yrhP	BSU27100	0.849
51	2739991	2740147	reverse	BSU26830	yrpE	-1287	0.3694	3533	aadK	BSU26790	0.849
**52**	644384	644540	forward	BSU05940	gcp	-7	0.3694	2120	moaC	BSU05960	0.848
53	4039599	4039755	forward	BSU39100	yxiO	-23552	0.4713	1806	hutP	BSU39340	0.847
54	2203622	2203778	forward	BSU20580	yoqM	-7279	0.363	79	yopS	BSU20780	0.847
55	3014345	3014501	reverse	BSU29460	moaB	-90	0.3694	79	argG	BSU29450	0.847
56	749147	749303	forward	BSU06780	yeeC	-3069	0.363	550	yeeG	BSU06820	0.846
57	665425	665581	forward	BSU06130	ydjC	-677	0.3439	1963	gutB	BSU06150	0.846
58	2106272	2106428	reverse	BSU19360	odhB	-1154	0.3949	79	yocR	BSU19340	0.846
59	226409	226565	forward	BSU02050	ybdO	-82	0.3885	79	ybxG	BSU02060	0.844
60	2106333	2106489	forward	BSU19330	sodF	-1353	0.3885	79	yocS	BSU19350	0.844
61	308175	308331	forward	BSU02840	ycdG	48	0.3503	79	adcA	BSU02850	0.843
62	2678925	2679081	forward	BSU26050	yqdB	-427	0.363	12639	yqaP	BSU26230	0.843
63	3875571	3875727	reverse	BSU37760	rocC	-130	0.3121	79	ywfA	BSU37750	0.843
64	2433680	2433836	reverse	BSU23340	ypuB	-384	0.3885	236	ypzJ	BSU23328	0.843
65	2879134	2879290	reverse	BSU28190	engB	-669	0.4013	79	hemA	BSU28170	0.842
66	1533806	1533962	forward	BSU14610	pdhD	-445	0.3503	236	ykzW	BSU14629	0.841
67	368137	368293	forward	BSU03360	yciC	-802	0.3312	1021	yckC	BSU03390	0.841
68	447000	447156	forward	BSU03930	gdh	-792	0.3121	2120	ycnL	BSU03970	0.84
69	3726630	3726786	forward	BSU36160	ywqM	-2216	0.4331	7144	ywqB	BSU36270	0.84
70	543132	543288	reverse	BSU05000	yddK	-2955	0.4904	11697	immR	BSU04820	0.84
71	3268320	3268476	forward	BSU31810	yuzE	-4017	0.4522	8557	yukF	BSU31920	0.84
72	2065804	2065960	forward	BSU18960	yozM	-348	0.3758	8557	yobN	BSU19020	0.839
73	45296	45452	reverse	BSU01550	gerD	-113140	0.3822	236	abrB	BSU00370	0.837
74	2048779	2048935	reverse	BSU18810	yobA	-1092	0.363	550	yoaZ	BSU18790	0.836
75	3153718	3153874	reverse	BSU30850	ytdA	-938	0.3439	79	menF	BSU30830	0.836
76	3388260	3388416	reverse	BSU33040	fumC	-685	0.3312	393	yuzO	BSU33029	0.834
77	205252	205408	forward	BSU01820	adaB	-283	0.3248	79	ndhF	BSU01830	0.834
78	469269	469425	forward	BSU04160	mtlR	24	0.3121	79	ydaB	BSU04170	0.834
79	1868460	1868616	forward	BSU17360	ymzA	-7	0.3885	79	nrdI	BSU17370	0.834
80	3746069	3746225	forward	BSU36380	rapD	-577	0.3822	2905	ywoH	BSU36440	0.833
81	3467327	3467483	reverse	BSU33800	opuCD	-140	0.3376	79	sdpR	BSU33790	0.832
82	1264932	1265088	reverse	BSU11990	yjdB	-4722	0.5223	79	yjcM	BSU11910	0.832
83	1903262	1903418	reverse	BSU17690	yncM	-170	0.3822	1963	cotU	BSU17670	0.831
84	4204441	4204597	reverse	BSU40960	parB	-1036	0.414	79	yyaD	BSU40940	0.831
85	1017114	1017270	forward	BSU09400	spoVR	18	0.3376	1806	lytE	BSU09420	0.831
86	2709577	2709733	reverse	BSU26490	yrkJ	-346	0.3503	236	yrkK	BSU26480	0.829
**87**	955738	955894	forward	BSU08780	ygaJ	-74	0.3822	79	thiC	BSU08790	0.828
88	554386	554542	reverse	BSU05130	ydeB	-5686	0.4395	1963	lrpB	BSU05060	0.828
**89**	3988764	3988920	reverse	BSU38860	galE	-1105	0.293	79	yxkD	BSU38840	0.825
90	2186812	2186968	reverse	BSU20420	yorD	-94	0.2611	79	yorE	BSU20410	0.825
91	2926840	2926996	reverse	BSU28630	pheT	-89	0.3185	1021	yshA	BSU28610	0.823
92	2054401	2054557	reverse	BSU18840	xynA	-119	0.3822	550	pps	BSU18830	0.822
93	610963	611119	reverse	BSU05660	ydgI	-1149	0.3121	2277	dinB	BSU05630	0.822
94	3457144	3457300	reverse	BSU33700	opuBD	-2583	0.3185	707	yvzC	BSU33650	0.821
95	736435	736591	reverse	BSU06740	yefB	-2481	0.3376	3690	yerO	BSU06700	0.82
96	2061478	2061634	reverse	BSU18930	yobH	-1953	0.3439	864	yozJ	BSU18900	0.82
97	2262616	2262772	reverse	BSU21440	bdbB	-2530	0.3885	15151	youB	BSU21329	0.819
98	4118717	4118873	reverse	BSU40110	bglA	-2370	0.3758	5574	glxK	BSU40040	0.818
99	4204755	4204911	reverse	BSU40960	parB	-722	0.3949	393	yyaD	BSU40940	0.818
100	3648264	3648420	reverse	BSU35530	tagO	-311	0.363	1806	degS	BSU35500	0.818

Top 50 hits of the forward and reverse strands of the *B. subtilis* intergenic regions using no-overlap 157 nt window and under the LMFEGCRND model. The ranking of each hit is denoted in column R. Distance from upstream and downstream operons are the distance from the center of the hit to the stop and start codons of upstream and downstream operons, respectively. Probability denotes the multinomial regression likelihood of being a riboswitch under the LMFEGCRND model.

Interesting hits are shown in bold.

**Table 23:** **Top classification hits in** ***B. subtilis*** **uracil-comp. constrained**

**R**	**Start**	**End**	**Strand**	**Upstream operon**	**Upstream gene**	**Dist. to upstream**	**Uracil**	**Dist. to downstream**	**Downstream gene**	**Downstream operon**	**Probability**
1	4134175	4134331	reverse	BSU40230	yydA	-182	0.3439	79	yydB	BSU40220	0.931
2	3714883	3715039	forward	BSU36030	ywrK	-859	0.3949	2277	cotG	BSU36070	0.922
3	3866327	3866483	reverse	BSU37690	ywfG	-1881	0.3503	79	eutD	BSU37660	0.903
4	681153	681309	forward	BSU06260	ydjN	-201	0.3885	5731	yeaB	BSU06320	0.899
5	1680274	1680430	forward	BSU16080	ylqH	63	0.3822	79	sucC	BSU16090	0.897
6	2316268	2316424	reverse	BSU22040	ypbQ	-99	0.363	236	ypbR	BSU22030	0.896
7	688027	688183	forward	BSU06320	yeaB	-114	0.3885	79	yeaC	BSU06330	0.893
8	243578	243734	forward	BSU02170	ybfB	-5370	0.363	236	purT	BSU02230	0.89
9	984466	984622	reverse	BSU09120	yhcK	-1189	0.3885	79	cspB	BSU09100	0.889
**10**	3421066	3421222	reverse	BSU33340	sspJ	-320	0.3312	79	lysP	BSU33330	0.885
11	1493630	1493786	forward	BSU14230	ykuV	-230	0.3503	79	rok	BSU14240	0.879
12	2531945	2532101	forward	BSU24210	yqiG	-14308	0.3439	9028	yqhQ	BSU24490	0.879
13	300673	300829	forward	BSU02770	yccK	-1196	0.3822	79	ycdB	BSU02790	0.875
14	4139318	4139474	forward	BSU40240	yycS	-3475	0.3567	864	rapG	BSU40300	0.865
15	3681213	3681369	forward	BSU35670	gtaB	-14627	0.363	79	tagA	BSU35750	0.864
16	3671690	3671846	reverse	BSU35700	tagH	-1795	0.3694	393	ggaA	BSU35690	0.859
**17**	191850	192006	forward	BSU01590	ybaS	-12186	0.3057	2277	trnSL-Glu2	BSU_tRNA_75	0.856
18	20723	20879	forward	BSU00120	yaaE	-86	0.3185	79	serS	BSU00130	0.856
19	2691445	2691601	reverse	BSU26240	yqaO	-1121	0.3439	79	yqaQ	BSU26220	0.852
20	3158851	3159007	reverse	BSU30890	ytxO	-328	0.363	3376	ytdA	BSU30850	0.852
21	1958206	1958362	forward	BSU18190	yngC	-9863	0.3949	44353	iseA	BSU18380	0.852
22	557716	557872	forward	BSU05100	yddT	-188	0.3503	79	ydzN	BSU05109	0.851
23	3907629	3907785	reverse	BSU38100	ywcH	-2594	0.3567	393	ywcI	BSU38080	0.851
24	1926523	1926679	forward	BSU17950	yneJ	-1482	0.3949	79	citB	BSU18000	0.851
25	1017271	1017427	forward	BSU09400	spoVR	-139	0.363	1649	lytE	BSU09420	0.85
26	2769617	2769773	reverse	BSU27160	cypB	-4194	0.3185	1021	yrhP	BSU27100	0.849
27	2739991	2740147	reverse	BSU26830	yrpE	-1287	0.3694	3533	aadK	BSU26790	0.849
**28**	644384	644540	forward	BSU05940	gcp	-7	0.3694	2120	moaC	BSU05960	0.848
29	2203622	2203778	forward	BSU20580	yoqM	-7279	0.363	79	yopS	BSU20780	0.847
30	3014345	3014501	reverse	BSU29460	moaB	-90	0.3694	79	argG	BSU29450	0.847
31	749147	749303	forward	BSU06780	yeeC	-3069	0.363	550	yeeG	BSU06820	0.846
32	665425	665581	forward	BSU06130	ydjC	-677	0.3439	1963	gutB	BSU06150	0.846
33	2106272	2106428	reverse	BSU19360	odhB	-1154	0.3949	79	yocR	BSU19340	0.846
34	226409	226565	forward	BSU02050	ybdO	-82	0.3885	79	ybxG	BSU02060	0.844
35	2106333	2106489	forward	BSU19330	sodF	-1353	0.3885	79	yocS	BSU19350	0.844
36	308175	308331	forward	BSU02840	ycdG	48	0.3503	79	adcA	BSU02850	0.843
37	2678925	2679081	forward	BSU26050	yqdB	-427	0.363	12639	yqaP	BSU26230	0.843
38	3875571	3875727	reverse	BSU37760	rocC	-130	0.3121	79	ywfA	BSU37750	0.843
39	2433680	2433836	reverse	BSU23340	ypuB	-384	0.3885	236	ypzJ	BSU23328	0.843
40	1533806	1533962	forward	BSU14610	pdhD	-445	0.3503	236	ykzW	BSU14629	0.841
41	368137	368293	forward	BSU03360	yciC	-802	0.3312	1021	yckC	BSU03390	0.841
42	447000	447156	forward	BSU03930	gdh	-792	0.3121	2120	ycnL	BSU03970	0.84
43	2065804	2065960	forward	BSU18960	yozM	-348	0.3758	8557	yobN	BSU19020	0.839
44	45296	45452	reverse	BSU01550	gerD	-113140	0.3822	236	abrB	BSU00370	0.837
45	2048779	2048935	reverse	BSU18810	yobA	-1092	0.363	550	yoaZ	BSU18790	0.836
46	3153718	3153874	reverse	BSU30850	ytdA	-938	0.3439	79	menF	BSU30830	0.836
47	3388260	3388416	reverse	BSU33040	fumC	-685	0.3312	393	yuzO	BSU33029	0.834
48	205252	205408	forward	BSU01820	adaB	-283	0.3248	79	ndhF	BSU01830	0.834
49	469269	469425	forward	BSU04160	mtlR	24	0.3121	79	ydaB	BSU04170	0.834
50	1868460	1868616	forward	BSU17360	ymzA	-7	0.3885	79	nrdI	BSU17370	0.834
51	3746069	3746225	forward	BSU36380	rapD	-577	0.3822	2905	ywoH	BSU36440	0.833
52	3467327	3467483	reverse	BSU33800	opuCD	-140	0.3376	79	sdpR	BSU33790	0.832
53	1903262	1903418	reverse	BSU17690	yncM	-170	0.3822	1963	cotU	BSU17670	0.831
54	1017114	1017270	forward	BSU09400	spoVR	18	0.3376	1806	lytE	BSU09420	0.831
55	2709577	2709733	reverse	BSU26490	yrkJ	-346	0.3503	236	yrkK	BSU26480	0.829
**56**	955738	955894	forward	BSU08780	ygaJ	-74	0.3822	79	thiC	BSU08790	0.828
57	1283149	1283305	forward	BSU12100	yjeA	-539	0.3758	236	yjfC	BSU12130	0.827
**58**	3988764	3988920	reverse	BSU38860	galE	-1105	0.293	79	yxkD	BSU38840	0.825
**59**	200120	200276	forward	BSU01770	glmM	-198	0.2611	79	glmS	BSU01780	0.825
60	2186812	2186968	reverse	BSU20420	yorD	-94	0.2611	79	yorE	BSU20410	0.825
61	2926840	2926996	reverse	BSU28630	pheT	-89	0.3185	1021	yshA	BSU28610	0.823
62	2054401	2054557	reverse	BSU18840	xynA	-119	0.3822	550	pps	BSU18830	0.822
63	610963	611119	reverse	BSU05660	ydgI	-1149	0.3121	2277	dinB	BSU05630	0.822
64	3457144	3457300	reverse	BSU33700	opuBD	-2583	0.3185	707	yvzC	BSU33650	0.821
65	736435	736591	reverse	BSU06740	yefB	-2481	0.3376	3690	yerO	BSU06700	0.82
66	2061478	2061634	reverse	BSU18930	yobH	-1953	0.3439	864	yozJ	BSU18900	0.82
67	3268477	3268633	forward	BSU31810	yuzE	-4174	0.3949	8400	yukF	BSU31920	0.82
68	3107044	3107200	forward	BSU30340	ytvA	30	0.2675	1492	yttA	BSU30360	0.82
69	2262616	2262772	reverse	BSU21440	bdbB	-2530	0.3885	15151	youB	BSU21329	0.819
70	4118717	4118873	reverse	BSU40110	bglA	-2370	0.3758	5574	glxK	BSU40040	0.818
71	4204755	4204911	reverse	BSU40960	parB	-722	0.3949	393	yyaD	BSU40940	0.818
72	252357	252513	forward	BSU02320	ybfP	36	0.3822	79	ybfQ	BSU02330	0.818
73	3648264	3648420	reverse	BSU35530	tagO	-311	0.363	1806	degS	BSU35500	0.818
74	850053	850209	forward	BSU07750	yflA	-3789	0.3694	236	treP	BSU07800	0.817
75	255279	255435	forward	BSU02330	ybfQ	-1718	0.2994	2434	ybgA	BSU02370	0.816
76	1541729	1541885	forward	BSU14680	ykzC	-2958	0.3376	79	ylaA	BSU14710	0.816
77	909862	910018	forward	BSU08330	yfiN	-658	0.3503	79	estB	BSU08350	0.816
78	4109617	4109773	reverse	BSU40030	yxaB	-1253	0.3885	79	yxaD	BSU40010	0.813
79	3252983	3253139	forward	BSU31660	mrpG	-538	0.3949	4632	yuzC	BSU31730	0.811
80	4066294	4066450	reverse	BSU39600	yxeC	-234	0.3567	864	yxeF	BSU39570	0.81
81	1923077	1923233	forward	BSU17910	yneF	-231	0.3248	79	ccdA	BSU17930	0.809
82	1540787	1540943	forward	BSU14680	ykzC	-2016	0.3503	1021	ylaA	BSU14710	0.809
83	3665472	3665628	forward	BSU35650	lytR	-1192	0.3376	79	gtaB	BSU35670	0.808
84	1679031	1679187	reverse	BSU17060	ymzD	-101508	0.3503	7458	ylqB	BSU15960	0.808
85	2698717	2698873	reverse	BSU26360	yqaD	-714	0.363	79	yqaF	BSU26340	0.808
86	3604725	3604881	reverse	BSU35100	yvlD	-1958	0.363	236	yvmC	BSU35070	0.808
87	3354671	3354827	forward	BSU32650	yurS	-105	0.3057	17820	yuzL	BSU32849	0.807
88	3052234	3052390	forward	BSU29710	acuC	-9600	0.3503	2434	ytoQ	BSU29850	0.806
**89**	188867	189023	forward	BSU01590	ybaS	-9203	0.3185	5260	trnSL-Glu2	BSU_tRNA_75	0.806
90	245389	245545	reverse	BSU02340	gltP	-8050	0.363	1806	ybfI	BSU02220	0.805
91	1445373	1445529	reverse	BSU13810	ykvS	-2210	0.3439	2748	ykvN	BSU13760	0.804
92	2249114	2249270	reverse	BSU21440	bdbB	-16032	0.3439	1649	youB	BSU21329	0.803
93	3918262	3918418	reverse	BSU38190	galT	-752	0.3057	79	qoxA	BSU38170	0.801
94	933760	933916	reverse	BSU08620	yfhP	-618	0.3439	5574	sspK	BSU08550	0.8
95	201248	201404	reverse	BSU01800	alkA	-1220	0.293	7301	ybbK	BSU01720	0.8
96	3684268	3684424	reverse	BSU35780	lytD	-479	0.3439	3376	tagD	BSU35740	0.8
97	2739834	2739990	reverse	BSU26830	yrpE	-1444	0.3439	3376	aadK	BSU26790	0.799
98	2252097	2252253	reverse	BSU21440	bdbB	-13049	0.3949	4632	youB	BSU21329	0.798
99	1601271	1601427	reverse	BSU15640	yloA	-34781	0.3503	24100	ylbP	BSU15100	0.797
100	2111609	2111765	reverse	BSU19380	yojO	-149	0.3439	79	sucA	BSU19370	0.796

Top 50 hits of the forward and reverse strands of the *B. subtilis* intergenic regions using no-overlap 157 nt window and under the LMFEGCRND model. Uracil composition constrained to that of the range of known riboswitches in *B. subtilis* (between 0.2484 and 0.40127). The ranking of each hit is denoted in column R. Distance from upstream and downstream operons are the distance from the center of the hit to the stop and start codons of upstream and downstream operons, respectively. Probability denotes the multinomial regression likelihood of being a riboswitch under the LMFEGCRND model.

Interesting hits are shown in bold.

**Table 24:** **Top entropy hits in** ***B. subtilis*** **filtered for GC-comp. and uracil-comp.**

***B. subtilis***	**Start**	**End**	**Strand**	**Upstream operon**	**Upstream gene**	**Dist. to upstream**	**MFE**	**MFE p. Val.**	**GC**	**RND**	**RND p. Val.**	**Uracil**	**Dist. to downstream**	**Downstream gene**	**Downstream operon**	**Probability**
157 nt	191850	192006	forward	BSU01590	ybaS	-12186	-54.16	0.01	0.4904	94.7470016	0.0359	0.3057	2277	trnSL-Glu2	BSU_tRNA_75	0.8561704159
157 nt	749147	749303	forward	BSU06780	yeeC	-3069	-49.19	-	0.4458	94.8936005	0.0310	0.3630	550	yeeG	BSU06820	0.8463344574
157 nt	665425	665581	forward	BSU06130	ydjC	-677	-51.50	-	0.4968	95.6813965	0.0169	0.3439	1963	gutB	BSU06150	0.8462108970
157 nt	1017114	1017270	forward	BSU09400	spoVR	18	-53.10	-	0.4968	94.5084991	0.0412	0.3376	1806	lytE	BSU09420	0.8305525184
157 nt	823013	823169	forward	BSU07480	yfmG	-604	-48.60	-	0.5032	94.2139969	0.0507	0.2866	4161	yfmA	BSU07540	0.7458834648
157 nt	3421066	3421222	reverse	BSU33340	sspJ	-320	-49.40	-	0.4713	97.1984024	0.0049	0.3312	79	lysP	BSU33330	0.8851321340
157 nt	3158851	3159007	reverse	BSU30890	ytxO	-328	-48.50	-	0.4395	95.1657028	0.0250	0.3630	3376	ytdA	BSU30850	0.8522043228
157 nt	736435	736591	reverse	BSU06740	yefB	-2481	-50.51	-	0.4904	95.0205994	0.0279	0.3376	3690	yerO	BSU06700	0.8204180002
157 nt	201248	201404	reverse	BSU01800	alkA	-1220	-49.46	-	0.4968	95.0683975	0.0269	0.2930	7301	ybbK	BSU01720	0.8003951907
157 nt	4129689	4129845	reverse	BSU40200	yydD	-810	-48.40	-	0.4904	94.8125000	0.0332	0.3567	2120	yydF	BSU40180	0.7834032774
150 nt	4134601	4134750	forward	BSU40190	fbp	-4483	-50.91	-	0.4733	91.2214966	-	0.3800	677	yycS	BSU40240	0.8779885173
150 nt	3359819	3359968	forward	BSU32650	yurS	-5258	-46.80	-	0.4600	92.3918991	-	0.3600	12677	yuzL	BSU32849	0.8770275712
150 nt	749175	749324	forward	BSU06780	yeeC	-3102	-46.50	-	0.4600	92.0363998	-	0.3867	527	yeeG	BSU06820	0.8652582169
150 nt	1958237	1958386	forward	BSU18190	yngC	-9899	-48.30	-	0.4733	90.9682007	-	0.3533	44327	iseA	BSU18380	0.8436317444
150 nt	1540761	1540910	forward	BSU14680	ykzC	-1995	-46.42	-	0.4333	90.3455963	-	0.3400	1052	ylaA	BSU14710	0.8428211212
150 nt	3199841	3199990	forward	BSU31170	yulF	-1875	-47.20	-	0.4467	89.9530029	-	0.3733	12677	tgl	BSU31270	0.8267914653
150 nt	3421066	3421215	reverse	BSU33340	sspJ	-325	-49.40	-	0.4800	93.4540024	-	0.3333	74	lysP	BSU33330	0.9072541595
150 nt	933665	933814	reverse	BSU08620	yfhP	-718	-49.54	-	0.4600	89.9813995	-	0.3733	5474	sspK	BSU08550	0.8426564932
200 nt	3359769	3359968	forward	BSU32650	yurS	-5183	-66.40	-	0.4600	123.4530029	-	0.3450	12702	yuzL	BSU32849	0.9236087203
200 nt	339225	339424	forward	BSU03130	nadE	-20	-63.60	-	0.4700	121.1809998	-	0.3250	702	aroK	BSU03150	0.8414211273
200 nt	1678852	1679051	reverse	BSU17060	ymzD	-101667	-62.81	-	0.4750	122.0299988	-	0.3300	7299	ylqB	BSU15960	0.8517054319
200 nt	3717398	3717597	reverse	BSU36100	ywrD	-1637	-51.30	-	0.3650	130.8540039	-	0.3950	399	cotH	BSU36060	0.9702541828
200 nt	198226	198425	reverse	BSU01800	alkA	-4222	-30.81	-	0.3750	130.7449951	-	0.5150	4299	ybbK	BSU01720	0.8267450333
157 nt	235800	235956	reverse	BSU02180	ybfE	-2285	-54.99	-	0.3312	66.4815979	0^1^	0.3439	550	glpT	BSU02140	0.0401644297
200 nt	3236257	3236456	forward	BSU31500	yuxK	61	-82.70	-	0.4200	93.3933029	0^2^	0.2650	802	yufL	BSU31520	0.0853443071

Significant hits of the forward and reverse strands of the *B. subtilis* intergenic regions having significantly high RND entropy (p-Val. <0.0500), significantly low (p.Val. <0.050), GC and uracil compositions within the range of those for known riboswitches Threshold values and their corresponding p-values have been calculated separately for each genome-wide test. No overlap used for 157 nt scan (28340 segments). 175 nt overlap used for 150 nt scan (60204 segments). 100 nt overlap used for 200 nt scan (44847 segments). Distance from upstream and downstream operons are the distance from the center of the hit to the stop and start codons of upstream and downstream operons, respectively. Probability denotes the multinomial regression likelihood of being a riboswitch under the LMFEGCRND model. Negative values indicate distance to upstream operon. Columns Upsream/Downstream Operon show gene ID within the operon.

^1^Table 24: The entropy of this sequence is the lowest within the test. The significance of this value is also shown in Figure 5 as the lowest blue point on the graph.

^2^Table 24: The entropy of this sequence is the lowest within the test.

**Table 25:** **Top entropy hits in** ***B. subtilis*** **forward strand**

***B. subtilis***	**Start**	**End**	**Strand**	**Upstream operon**	**Upstream gene**	**Dist. to upstream**	**MFE**	**MFE p. Val.**	**GC**	**RND**	**RND p. Val.**	**Uracil**	**Dist. to downstream**	**Downstream gene**	**Downstream operon**	**Probability**
200 nt	3714838	3715037	forward	BSU36030	ywrK	-794	-49.70	-	0.3300	126.0619965	-	0.3850	2302	cotG	BSU36070	0.9275143743
200 nt	3359769	3359968	forward	BSU32650	yurS	-5183	-66.40	-	0.4600	123.4530029	-	0.3450	12702	yuzL	BSU32849	0.9236087203
200 nt	243592	243791	forward	BSU02170	ybfB	-5364	-57.00	-	0.4450	126.5479965	-	0.3700	202	purT	BSU02230	0.9204539061
200 nt	2093202	2093401	forward	BSU19200	desR	-799	-55.20	-	0.4350	126.5859985	-	0.4400	4802	yoyB	BSU19259	0.9146069884
200 nt	749075	749274	forward	BSU06780	yeeC	-2977	-58.19	-	0.4450	125.6159973	-	0.3900	602	yeeG	BSU06820	0.9128865004
200 nt	1467005	1467204	forward	BSU13960	ykwC	-307	-60.40	-	0.4100	123.2139969	-	0.4150	702	pbpH	BSU13980	0.9070840478
200 nt	2281367	2281566	forward	BSU21620	yokE	95	-44.60	-	0.2600	124.4599991	-	0.4300	202	yokD	BSU21630	0.9058990479
200 nt	850067	850266	forward	BSU07750	yflA	-3783	-57.20	-	0.4000	124.1029968	-	0.3800	202	treP	BSU07800	0.9058393836
200 nt	1466905	1467104	forward	BSU13960	ykwC	-207	-56.93	-	0.3900	123.7220001	-	0.4200	802	pbpH	BSU13980	0.9029595852
200 nt	3759694	3759893	forward	BSU36530	bcrC	-467	-62.40	-	0.3850	121.3089981	-	0.4500	902	ywnH	BSU36560	0.9023656249
200 nt	3268355	3268554	forward	BSU31810	yuzE	-4032	-44.66	-	0.3750	127.7630005	-	0.4450	8502	yukF	BSU31920	0.8946693540
200 nt	2073039	2073238	forward	BSU18960	yozM	-7563	-52.00	-	0.3250	123.0869980	-	0.4450	1302	yobN	BSU19020	0.8944090009
200 nt	748975	749174	forward	BSU06780	yeeC	-2877	-58.80	-	0.4750	125.1039963	-	0.3750	702	yeeG	BSU06820	0.8876969814
200 nt	432172	432371	forward	BSU03780	phrC	-1988	-51.70	-	0.3050	122.0960007	-	0.3450	102	yclN	BSU03800	0.8850299716
200 nt	4039583	4039782	forward	BSU39100	yxiO	-23516	-53.00	-	0.4350	125.9430008	-	0.4350	1802	hutP	BSU39340	0.8843178153
200 nt	531587	531786	forward	BSU_tRNA_51	trnS-Leu2	-2066	-46.60	-	0.2650	122.5979996	-	0.3850	102	sacV	BSU04830	0.8803396225
200 nt	665366	665565	forward	BSU06130	ydjC	-598	-64.50	-	0.4900	122.9229965	-	0.3300	2002	gutB	BSU06150	0.8790112138
200 nt	3714938	3715137	forward	BSU36030	ywrK	-894	-41.70	-	0.3350	126.8040009	-	0.3550	2202	cotG	BSU36070	0.8768669963
200 nt	3685812	3686011	forward	BSU35770	tagC	-947	-51.80	-	0.3900	124.4950027	-	0.4150	2902	gerBA	BSU35800	0.8747953176
200 nt	2093302	2093501	forward	BSU19200	desR	-899	-61.40	-	0.4400	122.2649994	-	0.3700	4702	yoyB	BSU19259	0.8738172650
200 nt	1012447	1012646	forward	BSU09360	yhdC	-598	-51.20	-	0.3650	123.8079987	-	0.4100	3102	spoVR	BSU09400	0.8727893829
200 nt	955595	955794	forward	BSU08780	ygaJ	89	-59.79	-	0.4050	121.6190033	-	0.3600	202	thiC	BSU08790	0.8710888028
200 nt	3685912	3686111	forward	BSU35770	tagC	-1047	-53.60	-	0.3950	123.7610016	-	0.3950	2802	gerBA	BSU35800	0.8705116510
200 nt	4134651	4134850	forward	BSU40190	fbp	-4508	-58.20	-	0.4900	125.0049973	-	0.3900	602	yycS	BSU40240	0.8676616549
200 nt	45433	45632	forward	BSU00360	yabC	-535	-42.70	-	0.3000	124.7969971	-	0.4200	102	metS	BSU00380	0.8666248322
200 nt	2531951	2532150	forward	BSU24210	yqiG	-14294	-62.90	-	0.5050	123.5699997	-	0.3450	9002	yqhQ	BSU24490	0.8666005135
200 nt	2531951	2532150	forward	BSU24210	yqiG	-14294	-62.90	-	0.5050	123.5699997	-	0.3450	9002	yqhQ	BSU24490	0.8666005135
200 nt	1540786	1540985	forward	BSU14680	ykzC	-1995	-57.52	-	0.4350	123.3629990	-	0.3750	1002	ylaA	BSU14710	0.8663020134
200 nt	1406312	1406511	forward	BSU13390	ykoT	-1721	-71.55	-	0.5450	121.3809967	-	0.3250	3502	ykoX	BSU13430	0.8654530644
200 nt	4029283	4029482	forward	BSU39100	yxiO	-13216	-50.90	-	0.3850	124.3079987	-	0.4000	12102	hutP	BSU39340	0.8653051257
200 nt	1526531	1526730	forward	BSU14550	ykrA	-273	-63.20	-	0.4800	122.5350037	-	0.3350	602	ykyA	BSU14570	0.8648978472
200 nt	2220040	2220239	forward	BSU20929	yoyI	-6863	-38.72	-	0.3800	129.0460052	-	0.3700	2202	yonP	BSU21030	0.8647925854
200 nt	192105	192304	forward	BSU01590	ybaS	-12421	-51.20	-	0.4350	125.8399963	-	0.4100	2002	trnSL-Glu2	BSU_tRNA_75	0.8642573953
200 nt	1780406	1780605	forward	BSU17050	mutL	-87	-52.60	-	0.3550	122.5059967	-	0.3650	1402	pksA	BSU17080	0.8627771139
200 nt	4037783	4037982	forward	BSU39100	yxiO	-21716	-57.74	-	0.4400	123.2249985	-	0.3950	3602	hutP	BSU39340	0.8606380820
200 nt	1264357	1264556	forward	BSU_tRNA_83	trnSL-Val2	-1397	-33.05	-	0.2700	127.4010010	-	0.4200	602	yjcN	BSU11920	0.8592507839
200 nt	847767	847966	forward	BSU07750	yflA	-1483	-59.62	-	0.4500	122.6080017	-	0.3550	2502	treP	BSU07800	0.8554052114
200 nt	226266	226465	forward	BSU02050	ybdO	81	-37.20	-	0.2300	124.2030029	-	0.3550	202	ybxG	BSU02060	0.8548350334
200 nt	3052246	3052445	forward	BSU29710	acuC	-9592	-61.90	-	0.4900	123.0039978	-	0.3400	2402	ytoQ	BSU29850	0.8543979526
200 nt	530887	531086	forward	BSU_tRNA_51	trnS-Leu2	-1366	-42.20	-	0.3250	125.3190002	-	0.4000	802	sacV	BSU04830	0.8526363969
200 nt	2617117	2617316	forward	BSU25220	antE	-13743	-59.90	-	0.4500	122.3389969	-	0.4200	3502	yqeW	BSU25420	0.8511158824
200 nt	2221540	2221739	forward	BSU20929	yoyI	-8363	-46.30	-	0.2850	122.2210007	-	0.3450	702	yonP	BSU21030	0.8502966762
200 nt	2054178	2054377	forward	BSU18820	yobB	-3127	-48.70	-	0.3450	123.2819977	-	0.4000	2002	yobD	BSU18850	0.8502687216
200 nt	3042946	3043145	forward	BSU29710	acuC	-292	-49.94	-	0.4350	125.8190002	-	0.4150	11702	ytoQ	BSU29850	0.8499680161
200 nt	2780909	2781108	forward	BSU27150	yrhK	-7164	-47.80	-	0.3250	122.8980026	-	0.3750	202	yrhE	BSU27220	0.8482846022
200 nt	2723337	2723536	forward	BSU26630	yrdQ	-594	-44.00	-	0.2950	123.3580017	-	0.4300	2402	gltR	BSU26670	0.8465546370
200 nt	683762	683961	forward	BSU06260	ydjN	-2790	-59.40	-	0.4800	123.3310013	-	0.3600	3102	yeaB	BSU06320	0.8446838856
200 nt	3052346	3052545	forward	BSU29710	acuC	-9692	-56.62	-	0.4400	123.0849991	-	0.3800	2302	ytoQ	BSU29850	0.8442399502
200 nt	2405829	2406028	forward	BSU22869	ypzI	-12171	-60.30	-	0.4650	122.4049988	-	0.4150	3802	fer	BSU23040	0.8431282043
200 nt	579341	579540	forward	BSU05329	ydzO	-10	-47.40	-	0.3850	124.9130020	-	0.3050	102	aseR	BSU05330	0.8431255221
200 nt	748875	749074	forward	BSU06780	yeeC	-2777	-60.60	-	0.4600	122.0989990	-	0.3950	802	yeeG	BSU06820	0.8426845670
200 nt	339225	339424	forward	BSU03130	nadE	-20	-63.60	-	0.4700	121.1809998	-	0.3250	702	aroK	BSU03150	0.8414211273
200 nt	1251377	1251576	forward	BSU11730	cotO	-1930	-35.49	-	0.3200	127.4260025	-	0.4800	702	yjcA	BSU11790	0.8401102424
200 nt	3640353	3640552	forward	BSU35210	yvkA	-19956	-52.60	-	0.3600	121.8539963	-	0.4250	6302	yvyE	BSU35510	0.8399478197
200 nt	3686112	3686311	forward	BSU35770	tagC	-1247	-51.99	-	0.3750	122.5879974	-	0.4300	2602	gerBA	BSU35800	0.8393257856
200 nt	3686112	3686311	forward	BSU35770	tagC	-1247	-51.99	-	0.3750	122.5879974	-	0.4300	2602	gerBA	BSU35800	0.8393257856
200 nt	1494405	1494604	forward	BSU14240	rok	56	-52.70	-	0.4100	123.4729996	-	0.4150	1002	mobA	BSU14260	0.8389207721
200 nt	373532	373731	forward	BSU03410	bglC	-1741	-56.90	-	0.4500	123.1060028	-	0.3800	2402	hxlR	BSU03470	0.8382304311
200 nt	3686212	3686411	forward	BSU35770	tagC	-1347	-45.70	-	0.4000	125.9280014	-	0.4250	2502	gerBA	BSU35800	0.8377878666
200 nt	374532	374731	forward	BSU03410	bglC	-2741	-56.99	-	0.3750	120.5049973	-	0.3400	1402	hxlR	BSU03470	0.8375294805
200 nt	1540186	1540385	forward	BSU14680	ykzC	-1395	-61.11	-	0.4650	121.7969971	-	0.3500	1602	ylaA	BSU14710	0.8347978592
200 nt	360837	361036	forward	BSU03270	ycgT	-6870	-59.70	-	0.5000	123.5510025	-	0.3300	2002	nasA	BSU03330	0.8347288966
200 nt	213641	213840	forward	BSU01900	ybcM	-73	-36.30	-	0.2750	125.3079987	-	0.3950	202	skfA	BSU01910	0.8321032524
200 nt	739678	739877	forward	BSU06730	yefA	-597	-51.16	-	0.3900	123.1309967	-	0.4150	102	yefC	BSU06750	0.8301935792
200 nt	1495005	1495204	forward	BSU14240	rok	-544	-50.26	-	0.4150	124.2959976	-	0.3800	402	mobA	BSU14260	0.8287579417
200 nt	1541686	1541885	forward	BSU14680	ykzC	-2895	-43.10	-	0.3250	124.1309967	-	0.3650	102	ylaA	BSU14710	0.8283772469
200 nt	1268629	1268828	forward	BSU11970	yjcS	62	-40.70	-	0.2700	123.2060013	-	0.4250	102	yjdA	BSU11980	0.8273611665
200 nt	652232	652431	forward	BSU06030	groEL	-265	-37.70	-	0.2950	125.2659988	-	0.4500	1102	ydiM	BSU06040	0.8273396492
200 nt	2108093	2108292	forward	BSU19350	yocS	-539	-59.80	-	0.4700	122.2429962	-	0.3700	11202	yojI	BSU19440	0.8269666433
200 nt	728532	728731	forward	BSU06640	yerI	-2436	-46.30	-	0.3100	122.2779999	-	0.3600	102	gatC	BSU06670	0.8268005848
200 nt	1540686	1540885	forward	BSU14680	ykzC	-1895	-60.72	-	0.4350	120.6729965	-	0.3350	1102	ylaA	BSU14710	0.8265900016
200 nt	3746052	3746251	forward	BSU36380	rapD	-540	-57.10	-	0.4350	122.1200027	-	0.3850	2902	ywoH	BSU36440	0.8260388970
200 nt	1495105	1495304	forward	BSU14240	rok	-644	-55.30	-	0.3950	121.4899979	-	0.4000	302	mobA	BSU14260	0.8259468675
200 nt	1923034	1923233	forward	BSU17910	yneF	-168	-40.60	-	0.2500	122.5039978	-	0.3500	102	ccdA	BSU17930	0.8253148198
200 nt	746475	746674	forward	BSU06780	yeeC	-377	-31.51	-	0.2650	126.6760025	-	0.5000	3202	yeeG	BSU06820	0.8248795867
200 nt	2625315	2625514	forward	BSU25420	yqeW	-3576	-54.30	-	0.4850	124.8850021	-	0.3550	10402	rpsT	BSU25550	0.8240758777
200 nt	4007404	4007603	forward	BSU39020	yxjA	-360	-48.97	-	0.4150	124.6660004	-	0.3950	2902	citH	BSU39060	0.8239642382
200 nt	2376722	2376921	forward	BSU22510	ypjC	-15121	-44.40	-	0.3450	124.1429977	-	0.4000	16602	ypzI	BSU22869	0.8239628077
200 nt	3640453	3640652	forward	BSU35210	yvkA	-20056	-45.52	-	0.3450	123.6029968	-	0.4400	6202	yvyE	BSU35510	0.8211722970
200 nt	530787	530986	forward	BSU_tRNA_51	trnS-Leu2	-1266	-47.10	-	0.3350	122.6100006	-	0.3850	902	sacV	BSU04830	0.8206871748
200 nt	4139260	4139459	forward	BSU40240	yycS	-3397	-60.10	-	0.5050	123.1039963	-	0.3450	902	rapG	BSU40300	0.8204663396
200 nt	184305	184504	forward	BSU01590	ybaS	-4621	-44.20	-	0.4000	125.9649963	-	0.4400	9802	trnSL-Glu2	BSU_tRNA_75	0.8200225234
200 nt	792182	792381	forward	BSU07230	yetM	-656	-69.00	-	0.5400	120.5859985	-	0.3000	402	yetO	BSU07250	0.8170907497
200 nt	4007304	4007503	forward	BSU39020	yxjA	-260	-49.30	-	0.4200	124.4300003	-	0.3950	3002	citH	BSU39060	0.8151187301
200 nt	3726352	3726551	forward	BSU36160	ywqM	-1918	-63.90	-	0.5250	122.0380020	-	0.3200	7402	ywqB	BSU36270	0.8136813045
200 nt	3201391	3201590	forward	BSU31170	yulF	-3400	-50.17	-	0.4150	123.8629990	-	0.3950	11102	tgl	BSU31270	0.8136008978
200 nt	3714738	3714937	forward	BSU36030	ywrK	-694	-38.82	-	0.3050	124.7269974	-	0.4300	2402	cotG	BSU36070	0.8135811090
200 nt	2160707	2160906	forward	BSU20000	yosU	-1924	-34.70	-	0.3050	126.3850021	-	0.4300	9002	yosA	BSU20190	0.8132891059
200 nt	192805	193004	forward	BSU01590	ybaS	-13121	-48.80	-	0.4150	124.4029999	-	0.3650	1302	trnSL-Glu2	BSU_tRNA_75	0.8131257892
200 nt	2217640	2217839	forward	BSU20929	yoyI	-4463	-44.10	-	0.2800	121.7109985	-	0.4100	4602	yonP	BSU21030	0.8124790788
200 nt	182405	182604	forward	BSU01590	ybaS	-2721	-53.80	-	0.4150	122.3399963	-	0.3550	11702	trnSL-Glu2	BSU_tRNA_75	0.8117757440
200 nt	2276877	2277076	forward	BSU21520	yolC	-4287	-38.90	-	0.3250	125.3180008	-	0.4500	3002	yokF	BSU21610	0.8117634654
200 nt	1997137	1997336	forward	BSU18190	yngC	-48774	-50.97	-	0.4000	122.9609985	-	0.3250	5402	iseA	BSU18380	0.8112495542
200 nt	2276977	2277176	forward	BSU21520	yolC	-4387	-39.30	-	0.3200	124.9540024	-	0.4200	2902	yokF	BSU21610	0.8106592894
200 nt	749175	749374	forward	BSU06780	yeeC	-3077	-58.49	-	0.4600	121.9140015	-	0.3700	502	yeeG	BSU06820	0.8099753261
200 nt	3726652	3726851	forward	BSU36160	ywqM	-2218	-52.11	-	0.4550	124.3000031	-	0.3950	7102	ywqB	BSU36270	0.8091073036
200 nt	1251277	1251476	forward	BSU11730	cotO	-1830	-37.50	-	0.3650	127.1539993	-	0.5150	802	yjcA	BSU11790	0.8088676929
200 nt	1405212	1405411	forward	BSU13390	ykoT	-621	-56.26	-	0.4050	120.9140015	-	0.3500	4602	ykoX	BSU13430	0.8086636066
200 nt	3268455	3268654	forward	BSU31810	yuzE	-4132	-52.90	-	0.3900	121.7509995	-	0.4200	8402	yukF	BSU31920	0.8081850410
200 nt	1467105	1467304	forward	BSU13960	ykwC	-407	-41.17	-	0.3700	125.7789993	-	0.4650	602	pbpH	BSU13980	0.8068280816
200 nt	1406412	1406611	forward	BSU13390	ykoT	-1821	-67.24	-	0.5600	121.6240005	-	0.3150	3402	ykoX	BSU13430	0.8052443266
200 nt	2897488	2897687	forward	BSU28180	ysxD	-17529	-28.00	-	0.2150	125.7649994	-	0.4100	202	ysnD	BSU28320	0.8036175966
200 nt	1474672	1474871	forward	BSU14010	cheV	-57	-50.60	-	0.3800	122.1959991	-	0.3650	3302	ykuF	BSU14060	0.8031342626
200 nt	746375	746574	forward	BSU06780	yeeC	-277	-31.50	-	0.2800	126.4580002	-	0.5000	3302	yeeG	BSU06820	0.8004485369

Significant hits of the forward and reverse strands (only showing forward strand here) of the *B. subtilis* intergenic regions having significantly high RND entropy (p-Val. <0.0500) and LMFEGCRND probability higher than 0.8. 100 nt overlap used for 200 nt scan (44847 segments). Distance from upstream and downstream operons are the distance from the center of the hit to the stop and start codons of upstream and downstream operons, respectively. Probability denotes the likelihood of being a riboswitch under the LMFEGCRND model. Negative values indicate distance to upstream operon. Columns Upsream/Downstream Operon show gene ID within the operon.

**Table 26:** **Top entropy hits in** ***B. subtilis*** **reverse strand**

***B. subtilis***	**Start**	**End**	**Strand**	**Upstream operon**	**Upstream gene**	**Dist. to upstream**	**MFE**	**MFE p. Val.**	**GC**	**RND**	**RND p. Val.**	**Uracil**	**Dist. to downstream**	**Downstream gene**	**Downstream operon**	**Probability**
200 nt	3717398	3717597	reverse	BSU36100	ywrD	-1637	-51.30	-	0.3650	130.8540039	-	0.3950	399	cotH	BSU36060	0.9702541828
200 nt	3717498	3717697	reverse	BSU36100	ywrD	-1537	-50.60	-	0.3500	129.2720032	-	0.4000	499	cotH	BSU36060	0.9603169560
200 nt	4066209	4066408	reverse	BSU39600	yxeC	-299	-67.50	-	0.4900	125.4860001	-	0.3650	799	yxeF	BSU39570	0.9434255362
200 nt	786306	786505	reverse	BSU07220	yetL	-3247	-72.30	-	0.5600	125.9049988	-	0.3050	499	yetH	BSU07160	0.9432973266
200 nt	2249144	2249343	reverse	BSU21440	bdbB	-15982	-49.00	-	0.3700	127.3170013	-	0.3850	1699	youB	BSU21329	0.9216341376
200 nt	2596201	2596400	reverse	BSU25170	yqfO	-1202	-42.96	-	0.3650	129.5290070	-	0.4950	799	cshB	BSU25140	0.9206426144
200 nt	3717298	3717497	reverse	BSU36100	ywrD	-1737	-45.43	-	0.3750	128.8240051	-	0.4200	299	cotH	BSU36060	0.9199228883
200 nt	3671576	3671775	reverse	BSU35700	tagH	-1889	-33.00	-	0.2350	128.6049957	-	0.4550	299	ggaA	BSU35690	0.9113640189
200 nt	3717598	3717797	reverse	BSU36100	ywrD	-1437	-50.50	-	0.3850	126.3860016	-	0.4350	599	cotH	BSU36060	0.9073441625
200 nt	3373949	3374148	reverse	BSU32890	yusQ	-2569	-67.60	-	0.4800	122.0930023	-	0.3600	399	fadM	BSU32850	0.8957566023
200 nt	3666584	3666783	reverse	BSU35680	ggaB	-526	-44.06	-	0.3300	126.5329971	-	0.4650	1299	mnaA	BSU35660	0.8957416415
200 nt	3941142	3941341	reverse	BSU38430	gspA	-3319	-43.33	-	0.3950	128.7100067	-	0.4800	1599	ywbA	BSU38390	0.8889677525
200 nt	2879134	2879333	reverse	BSU28190	engB	-649	-38.80	-	0.2800	126.4779968	-	0.4100	99	hemA	BSU28170	0.8852627277
200 nt	3907615	3907814	reverse	BSU38100	ywcH	-2588	-46.12	-	0.2950	123.9560013	-	0.3600	399	ywcI	BSU38080	0.8837128878
200 nt	1248822	1249021	reverse	BSU11740	cotZ	-521	-54.90	-	0.5300	128.1889954	-	0.4000	8799	yjbP	BSU11630	0.8796436787
200 nt	2004047	2004246	reverse	BSU18400	yoeD	-116	-40.10	-	0.3150	126.8629990	-	0.3900	199	yoeC	BSU18390	0.8789740205
200 nt	3671676	3671875	reverse	BSU35700	tagH	-1789	-35.80	-	0.2600	126.5380020	-	0.3900	399	ggaA	BSU35690	0.8741892576
200 nt	2257744	2257943	reverse	BSU21440	bdbB	-7382	-38.35	-	0.3350	128.0359955	-	0.4600	10299	youB	BSU21329	0.8739098310
200 nt	2294238	2294437	reverse	BSU21800	ypkP	-1645	-57.60	-	0.3900	122.0520020	-	0.3950	299	ilvA	BSU21770	0.8724481463
200 nt	791156	791355	reverse	BSU07240	yetN	-207	-70.84	-	0.5850	123.2480011	-	0.2900	1099	yetL	BSU07220	0.8711011410
200 nt	494742	494941	reverse	BSU04430	ydbD	-899	-52.90	-	0.4250	125.0230026	-	0.4350	1399	ydaT	BSU04380	0.8695754409
200 nt	1355092	1355291	reverse	BSU12900	htrA	-2745	-71.91	-	0.5400	121.2139969	-	0.3050	2499	ykbA	BSU12860	0.8692007065
200 nt	737924	738123	reverse	BSU06740	yefB	-972	-70.30	-	0.5400	121.8629990	-	0.3150	5199	yerO	BSU06700	0.8691427112
200 nt	937998	938197	reverse	BSU08700	ygaE	-3071	-54.20	-	0.4350	124.7170029	-	0.4200	1099	yfhS	BSU08640	0.8665797114
200 nt	3421066	3421265	reverse	BSU33340	sspJ	-300	-64.50	-	0.4850	122.1780014	-	0.3150	99	lysP	BSU33330	0.8648849726
200 nt	2739937	2740136	reverse	BSU26830	yrpE	-1321	-49.69	-	0.4200	125.9660034	-	0.3800	3499	aadK	BSU26790	0.8648592830
200 nt	1097850	1098049	reverse	BSU10230	yhfH	-171	-36.90	-	0.2550	125.4639969	-	0.4450	99	gltT	BSU10220	0.8626419306
200 nt	3851617	3851816	reverse	BSU37520	ywhD	-470	-68.42	-	0.5500	122.7009964	-	0.3000	2099	speE	BSU37500	0.8624630570
200 nt	2724828	2725027	reverse	BSU26660	yrdN	-187	-37.44	-	0.2200	124.0479965	-	0.3800	99	czcD	BSU26650	0.8623370528
200 nt	1204503	1204702	reverse	BSU11270	yjzD	-129	-59.84	-	0.5050	124.6380005	-	0.3600	13299	yitU	BSU11140	0.8622197509
200 nt	2692345	2692544	reverse	BSU26240	yqaO	-201	-47.40	-	0.3350	123.8980026	-	0.4100	999	yqaQ	BSU26220	0.8618891239
200 nt	3108926	3109125	reverse	BSU30370	bceB	-372	-32.52	-	0.3250	129.4949951	-	0.5250	599	yttB	BSU30350	0.8596788645
200 nt	1493525	1493724	reverse	BSU14250	yknT	-779	-39.60	-	0.2150	122.8750000	-	0.4750	1599	ykuT	BSU14210	0.8589127660
200 nt	2111609	2111808	reverse	BSU19380	yojO	-129	-37.20	-	0.2700	125.5370026	-	0.4050	99	sucA	BSU19370	0.8542534709
200 nt	1678852	1679051	reverse	BSU17060	ymzD	-101667	-62.81	-	0.4750	122.0299988	-	0.3300	7299	ylqB	BSU15960	0.8517054319
200 nt	4109617	4109816	reverse	BSU40030	yxaB	-1233	-52.14	-	0.3400	121.7229996	-	0.3900	99	yxaD	BSU40010	0.8503260016
200 nt	3373849	3374048	reverse	BSU32890	yusQ	-2669	-56.90	-	0.5000	125.1539993	-	0.3500	299	fadM	BSU32850	0.8485122919
200 nt	1886780	1886979	reverse	BSU17590	xylR	-3633	-42.30	-	0.3150	124.7409973	-	0.3500	13299	cwlC	BSU17410	0.8470579386
200 nt	3153718	3153917	reverse	BSU30850	ytdA	-918	-51.80	-	0.3600	122.3779984	-	0.3500	99	menF	BSU30830	0.8457853198
200 nt	984466	984665	reverse	BSU09120	yhcK	-1169	-40.11	-	0.2800	124.3939972	-	0.3750	99	cspB	BSU09100	0.8457109332
200 nt	3464551	3464750	reverse	BSU33780	sdpI	-1784	-40.60	-	0.3100	125.1740036	-	0.4450	1399	opuBA	BSU33730	0.8446103930
200 nt	3684271	3684470	reverse	BSU35780	lytD	-456	-43.90	-	0.3450	125.0149994	-	0.3500	3399	tagD	BSU35740	0.8443253636
200 nt	2770775	2770974	reverse	BSU27160	cypB	-3016	-41.20	-	0.3550	126.4410019	-	0.4050	2199	yrhP	BSU27100	0.8441781402
200 nt	1666288	1666487	reverse	BSU15960	ylqB	-4779	-56.60	-	0.4500	123.3949966	-	0.3650	10599	rpmB	BSU15820	0.8431691527
200 nt	2343862	2344061	reverse	BSU22330	ypoC	-303	-43.50	-	0.3800	126.2779999	-	0.4850	3199	yppC	BSU22300	0.8418609500
200 nt	3158854	3159053	reverse	BSU30890	ytxO	-305	-56.71	-	0.4200	122.1419983	-	0.3650	3399	ytdA	BSU30850	0.8375009298
200 nt	4129648	4129847	reverse	BSU40200	yydD	-831	-66.70	-	0.5050	120.9280014	-	0.3350	2099	yydF	BSU40180	0.8359215260
200 nt	4134175	4134374	reverse	BSU40230	yydA	-162	-30.70	-	0.2450	126.6350021	-	0.3400	99	yydB	BSU40220	0.8344783783
200 nt	245362	245561	reverse	BSU02340	gltP	-8057	-57.10	-	0.4500	122.8600006	-	0.3500	1799	ybfI	BSU02220	0.8332447410
200 nt	3996389	3996588	reverse	BSU38970	yxjF	-4051	-40.50	-	0.3750	126.9710007	-	0.4250	4899	yxkA	BSU38870	0.8313001394
200 nt	2739837	2740036	reverse	BSU26830	yrpE	-1421	-64.40	-	0.4950	121.3069992	-	0.3300	3399	aadK	BSU26790	0.8294041157
200 nt	1903178	1903377	reverse	BSU17690	yncM	-234	-35.20	-	0.2700	125.4830017	-	0.4250	1899	cotU	BSU17670	0.8289884329
200 nt	933665	933864	reverse	BSU08620	yfhP	-693	-62.04	-	0.4700	121.3809967	-	0.3750	5499	sspK	BSU08550	0.8283531070
200 nt	2054430	2054629	reverse	BSU18840	xynA	-70	-52.10	-	0.3850	122.5159988	-	0.3450	599	pps	BSU18830	0.8281581998
200 nt	737824	738023	reverse	BSU06740	yefB	-1072	-69.00	-	0.5350	120.7480011	-	0.3100	5099	yerO	BSU06700	0.8277365565
200 nt	198226	198425	reverse	BSU01800	alkA	-4222	-30.81	-	0.3750	130.7449951	-	0.5150	4299	ybbK	BSU01720	0.8267450333
200 nt	3604668	3604867	reverse	BSU35100	yvlD	-1995	-39.30	-	0.2750	123.9219971	-	0.3550	199	yvmC	BSU35070	0.8267388940
200 nt	3098465	3098664	reverse	BSU30310	ytwF	-3637	-48.30	-	0.3850	123.9609985	-	0.4100	1999	ytaP	BSU30250	0.8252500296
200 nt	419514	419713	reverse	BSU03690	yczF	-150	-54.00	-	0.4400	123.4860001	-	0.3300	1899	dtpT	BSU03670	0.8242135644
200 nt	2221888	2222087	reverse	BSU21080	yonI	-6769	-36.60	-	0.3550	127.6279984	-	0.3150	599	yonR	BSU21020	0.8236665726
200 nt	2434023	2434222	reverse	BSU23340	ypuB	-21	-56.65	-	0.4750	123.5520020	-	0.3050	599	ypzJ	BSU23328	0.8226841688
200 nt	3241980	3242179	reverse	BSU31590	yufS	-4073	-57.60	-	0.4600	122.6460037	-	0.3500	5099	yufK	BSU31510	0.8222519755
200 nt	1700752	1700951	reverse	BSU17060	ymzD	-79767	-52.70	-	0.4300	123.5100021	-	0.3150	29199	ylqB	BSU15960	0.8189544678
200 nt	2709820	2710019	reverse	BSU26490	yrkJ	-83	-48.40	-	0.3600	122.8440018	-	0.3550	499	yrkK	BSU26480	0.8178487420
200 nt	153939	154138	reverse	BSU01550	gerD	-4477	-50.70	-	0.4100	123.6019974	-	0.3700	108899	abrB	BSU00370	0.8176639676
200 nt	3239080	3239279	reverse	BSU31590	yufS	-6973	-58.30	-	0.4150	120.6800003	-	0.3750	2199	yufK	BSU31510	0.8171101809
200 nt	3467327	3467526	reverse	BSU33800	opuCD	-120	-32.10	-	0.2350	125.1719971	-	0.3550	99	sdpR	BSU33790	0.8168275952
200 nt	543114	543313	reverse	BSU05000	yddK	-2953	-43.99	-	0.3600	124.5599976	-	0.4700	11699	immR	BSU04820	0.8156080246
200 nt	3108826	3109025	reverse	BSU30370	bceB	-472	-37.52	-	0.3400	126.4919968	-	0.4900	499	yttB	BSU30350	0.8153505921
200 nt	3334388	3334587	reverse	BSU32470	pucE	-1264	-54.50	-	0.4850	124.4670029	-	0.3050	5899	pucH	BSU32410	0.8130649924
200 nt	3684371	3684570	reverse	BSU35780	lytD	-356	-42.00	-	0.3250	124.1009979	-	0.3650	3499	tagD	BSU35740	0.8130072355
200 nt	881307	881506	reverse	BSU08120	yfjF	-4438	-52.36	-	0.4850	125.3059998	-	0.3900	9099	yfjQ	BSU08000	0.8121696115
200 nt	2926798	2926997	reverse	BSU28630	pheT	-111	-60.46	-	0.4950	122.2929993	-	0.3200	999	yshA	BSU28610	0.8096395731
200 nt	4066309	4066508	reverse	BSU39600	yxeC	-199	-55.60	-	0.4600	123.0559998	-	0.3600	899	yxeF	BSU39570	0.8090547919
200 nt	3688648	3688847	reverse	BSU35830	ywtG	-3786	-49.93	-	0.3300	120.9260025	-	0.3550	199	yvyI	BSU35790	0.8084035516
200 nt	1668788	1668987	reverse	BSU15960	ylqB	-2279	-51.60	-	0.4050	122.7809982	-	0.4150	13099	rpmB	BSU15820	0.8080439568
200 nt	3334288	3334487	reverse	BSU32470	pucE	-1364	-62.06	-	0.4700	120.7320023	-	0.2950	5799	pucH	BSU32410	0.8073683381
200 nt	3723732	3723931	reverse	BSU36170	ywqL	-589	-55.50	-	0.4550	122.8669968	-	0.3300	499	ywqN	BSU36150	0.8070057034
200 nt	899787	899986	reverse	BSU08340	padR	-9312	-54.60	-	0.3900	121.0039978	-	0.3550	10199	yfjA	BSU08170	0.8061554432
200 nt	2813434	2813633	reverse	BSU27540	yrvM	-110	-48.20	-	0.3850	123.3710022	-	0.4200	1399	cymR	BSU27520	0.8043378592
200 nt	3458216	3458415	reverse	BSU33700	opuBD	-1491	-44.09	-	0.3500	123.8420029	-	0.4200	1799	yvzC	BSU33650	0.8041363358
200 nt	3918262	3918461	reverse	BSU38190	galT	-732	-37.50	-	0.2900	124.4739990	-	0.3100	99	qoxA	BSU38170	0.8040998578
200 nt	2576788	2576987	reverse	BSU24950	pstBB	-323	-53.30	-	0.3700	120.7910004	-	0.4100	699	yqgL	BSU24920	0.8040402532
200 nt	2316211	2316410	reverse	BSU22040	ypbQ	-136	-50.40	-	0.3350	120.7730026	-	0.3850	199	ypbR	BSU22030	0.8038558364
200 nt	2249344	2249543	reverse	BSU21440	bdbB	-15782	-43.38	-	0.3500	124.1179962	-	0.4650	1899	youB	BSU21329	0.8037469983
200 nt	2333112	2333311	reverse	BSU22210	yprB	-113	-43.21	-	0.3300	123.4840012	-	0.4200	199	cotD	BSU22200	0.8028732538
200 nt	2116670	2116869	reverse	BSU19420	yojK	-282	-43.45	-	0.3300	123.3679962	-	0.3150	99	cwlS	BSU19410	0.8022140265
200 nt	3941242	3941441	reverse	BSU38430	gspA	-3219	-42.84	-	0.4300	126.9950027	-	0.4600	1699	ywbA	BSU38390	0.8019362092
200 nt	1248722	1248921	reverse	BSU11740	cotZ	-621	-64.39	-	0.5300	121.6650009	-	0.3550	8699	yjbP	BSU11630	0.8019282818
200 nt	3014345	3014544	reverse	BSU29460	moaB	-70	-39.90	-	0.2550	122.2269974	-	0.3300	99	argG	BSU29450	0.8009542227
200 nt	2542225	2542424	reverse	BSU24510	yqhO	-115	-57.14	-	0.4800	122.8730011	-	0.4100	1499	yqhR	BSU24480	0.8008475304
200 nt	2096086	2096285	reverse	BSU19230	yocJ	-165	-39.50	-	0.2800	123.2190018	-	0.4150	399	yocI	BSU19220	0.8003621101
200 nt	4107928	4108127	reverse	BSU40010	yxaD	-1158	-51.01	-	0.4150	123.1330032	-	0.3800	99	yxaF	BSU39990	0.8002312183
200 nt	2659771	2659970	reverse	BSU25880	yqxJ	-3681	-49.60	-	0.3800	122.5149994	-	0.3600	1099	yqcI	BSU25820	0.8001416922

Significant hits of the forward and reverse strands (only showing reverse strand here) of the *B. subtilis* intergenic regions having significantly high RND entropy (p-Val. <0.0500) and LMFEGCRND probability higher than 0.8. 100 nt overlap used for 200 nt scan (44847 segments). Distance from upstream and downstream operons are the distance from the center of the hit to the stop and start codons of upstream and downstream operons, respectively. Probability denotes the multinomial regression likelihood of being a riboswitch under the LMFEGCRND model. Negative values indicate distance to upstream operon. Columns Upsream/Downstream Operon show gene ID within the operon.

#### *Escherichia coli* and *Synechococcus elongatus*

Nine out of the 29 riboswitches in the training set are from the *E. coli* genome. As a test of the generality of the results on *B. subtilis*, we evaluated the performance of the three classifiers on various constant-length riboswitches, 100 nt, 150 nt, 157 nt, and 200 nt on *E. coli*. The performance of the LMFEGCRND classifier for the 100 nt-constant length was slightly higher than other tests (data not shown). Hence, the 100 nt constant-length window scan of 50 nt overlap was used to examine the intergenic regions of *E. coli*. The operon coordinates were taken from RegulonDB website [84]. Top 50 hits on each strands are available in Table 27. Top 50 hits having uracil compositions within the range of known riboswitches are organized in Table 28. The genomic distribution of the latter set is shown in Figure 6. Sequence segments having significant MFE and high structural entropy values are sorted in Tables 29 and 30 for significant and insignificant structural entropy values, respectively.

**Table 27:** **Top classification hits in** ***E. coli***

**R**	**Start**	**End**	**Strand**	**Upstream operon**	**Dist. to upstream**	**Uracil**	**Dist. to downstream**	**Downstream operon**	**Probability**
1	384006	384105	forward	insC-1,insCD-1,insD-1	-2154	0.52	402	tauA,tauB,tauC,tauD	0.942
2	237185	237284	forward	aspV	-129	0.47	102	yafT	0.934
3	2777119	2777218	forward	yfjX,yfjY,yfjZ,ypjF,ypjJ	-1266	0.38	7252	ygaQ_1,ygaQ_2	0.925
4	2304856	2304955	forward	eco	-2392	0.45	6202	micF	0.923
5	83968	84067	forward	setA,sgrS,sgrT	-5120	0.49	352	leuO	0.92
6	2902496	2902595	reverse	queE	-224	0.48	4249	ygcW	0.918
7	294815	294914	forward	yagJ	-3311	0.43	7352	yagU	0.914
8	4554566	4554665	forward	uxuR	-1145	0.48	402	iraD	0.913
9	405479	405578	forward	yaiI	16	0.45	102	aroL,aroM,yaiA	0.908
10	4570237	4570336	forward	yjiS	-250	0.38	152	yjiT	0.906
11	754000	754099	forward	nei,ybgI,ybgJ,ybgK,ybgL	-8002	0.4	352	sdhA^1^	0.905
12	2054653	2054752	reverse	asnW	-1349	0.44	3349	yeeL_1,yeeL_2	0.905
13	2202241	2202340	reverse	yehS	-7458	0.44	10099	mrp	0.903
14	330995	331094	forward	betT	-226	0.52	552	yahA	0.9
15	3183291	3183390	reverse	glgS	-6421	0.44	599	ribB,sroG	0.9
16	384056	384155	forward	insC-1,insCD-1,insD-1	-2204	0.43	352	tauA,tauB,tauC,tauD	0.898
17	4570187	4570286	forward	yjiS	-200	0.42	202	yjiT	0.898
18	557285	557384	forward	cysS	-2017	0.35	102	sfmA	0.894
19	3190062	3190161	reverse	sibD	-2632	0.37	149	glgS	0.893
20	1543575	1543674	forward	nhoA	-10633	0.45	1802	fdnG,fdnH,fdnI	0.89
21	2190295	2190394	reverse	yehE	-193	0.46	99	yehA,yehB,yehC,yehD	0.89
22	3181507	3181606	reverse	ribB,sroG	-279	0.43	1099	ygiD	0.89
23	3834703	3834802	reverse	nlpA	-2446	0.37	799	yicI,yicJ	0.889
24	1753166	1753265	reverse	ynhG	-2530	0.45	49	ydhZ	0.888
25	819916	820015	forward	ybhL	-56	0.41	52	ybhM	0.887
26	2901746	2901845	reverse	queE	-974	0.51	3499	ygcW	0.887
27	651208	651307	forward	ybdR	-8610	0.49	202	dpiA,dpiB	0.886
28	584973	585072	forward	appY	-1271	0.57	9802	cusA,cusB,cusC,cusF	0.886
29	2362398	2362497	reverse	ais	-593	0.48	149	yfaZ	0.886
30	1596214	1596313	forward	osmC	-41085	0.39	3252	lsrA,lsrB,lsrC,lsrD,lsrF,lsrG,tam	0.882
31	522335	522434	forward	ybbA,ybbP	-232	0.39	102	rhsD,ybbC,ybbD,ylbH	0.881
32	3490420	3490519	reverse	php,yhfS,yhfT,yhfU,yhfW,yhfX	-12488	0.39	149	ppiA	0.88
33	1986023	1986122	reverse	yecH	-1203	0.45	49	isrB	0.879
34	3217299	3217398	reverse	ygjH	-1589	0.42	249	aer	0.878
35	2714626	2714725	forward	eamB	-545	0.45	102	ung	0.877
**36**	3984255	3984354	forward	aslB	-1990	0.42	152	glmZ	0.877
37	2651611	2651710	reverse	sseB	-519	0.5	99	C0614	0.877
38	4516300	4516399	forward	insO-2,yjhV,yjhW	-8095	0.39	202	insA-7	0.876
39	3886253	3886352	reverse	purP	-6993	0.31	4549	dnaA,dnaN,recF	0.876
40	1577414	1577513	forward	osmC	-22285	0.41	22052	lsrA,lsrB,lsrC,lsrD,lsrF,lsrG,tam	0.875
41	776349	776448	reverse	zitB	-6707	0.39	11299	mngR	0.874
42	1543625	1543724	forward	nhoA	-10683	0.46	1752	fdnG,fdnH,fdnI	0.871
43	29201	29300	forward	dapB	43	0.49	402	carA,carB	0.871
44	1542975	1543074	forward	nhoA	-10033	0.39	2402	fdnG,fdnH,fdnI	0.871
45	3768179	3768278	reverse	yibH,yibI	-38	0.41	8249	yibF	0.871
46	3631114	3631213	forward	yhhI	-7528	0.4	1702	yhiM	0.87
47	2166486	2166585	forward	cyaR	-1213	0.41	202	yegS	0.87
48	4578972	4579071	forward	symR	-989	0.38	5952	mrr	0.869
49	522235	522334	forward	ybbA,ybbP	-132	0.39	202	rhsD,ybbC,ybbD,ylbH	0.869
50	2383795	2383894	reverse	yfbN	-1888	0.48	99	yfbK	0.869
51	4577258	4577357	forward	yjiV	-2331	0.36	552	symR	0.868
52	153855	153954	forward	yadD	-5936	0.37	8202	hrpB	0.868
53	3665704	3665803	reverse	yhjA	-61	0.42	149	gadA,gadW,gadX	0.868
54	3651672	3651771	reverse	hdeA,hdeB,yhiD	-1557	0.45	499	insH-11	0.868
55	4619642	4619741	forward	deoA,deoB,deoC,deoD	32	0.38	102	yjjJ	0.867
56	1645875	1645974	reverse	ynfP	-4828	0.45	49	dicC,ydfW,ydfX	0.867
57	4554516	4554615	forward	uxuR	-1095	0.42	452	iraD	0.866
58	4539860	4539959	forward	fimB	-229	0.41	152	fimE	0.866
59	1588711	1588810	reverse	hipA,hipB	-118	0.4	199	yneL	0.866
60	3181557	3181656	reverse	ribB,sroG	-229	0.33	1149	ygiD	0.865
61	269657	269756	reverse	insH-1	-3619	0.45	299	perR	0.864
62	3925028	3925127	forward	cbrB,cbrC	-28347	0.47	102	asnA	0.863
63	4538580	4538679	forward	yjhR	-4477	0.43	352	fimB	0.863
64	4077095	4077194	reverse	fdhE,fdoG,fdoH,fdoI	-1178	0.39	5549	yihS,yihT,yihU	0.863
65	1210379	1210478	reverse	iraM	-475	0.42	1549	stfE,tfaE	0.862
66	4213351	4213450	forward	metA	-70	0.37	102	aceA,aceB,aceK	0.861
67	578853	578952	forward	essD,rrrD,rzpD	-1013	0.49	202	ybcW	0.861
68	3755951	3756050	reverse	selA,selB	-40	0.33	149	yiaY	0.861
69	924768	924867	forward	clpA	44	0.36	7002	lrp	0.86
70	2520650	2520749	reverse	xapA,xapB	-52	0.42	199	xapR	0.86
71	582454	582553	forward	tfaX	-122	0.44	402	appY	0.859
72	1049984	1050083	forward	insA-4,insAB-4,insB-4	-182	0.38	652	cspG	0.859
73	3767704	3767803	forward	yibG	-994	0.36	2552	mtlA,mtlD,mtlR	0.859
74	2383745	2383844	reverse	yfbN	-1938	0.45	49	yfbK	0.859
75	1005025	1005124	forward	pyrD	25	0.46	102	zapC	0.858
76	157105	157204	forward	yadD	-9186	0.34	4952	hrpB	0.858
77	1669567	1669666	reverse	mdtI,mdtJ	-1228	0.46	1999	ynfL	0.858
78	3800263	3800362	forward	rfaD,waaC,waaF,waaL	-3984	0.35	6252	coaD,waaA	0.856
79	2784960	2785059	reverse	ygaU	-9350	0.41	1149	ileY	0.856
80	2468130	2468229	reverse	yfdK,yfdL,yfdM,yfdN,yfdO	-920	0.49	5149	mlaA	0.855
81	2991883	2991982	reverse	ygeK,ygeL	-550	0.39	3549	yqeK	0.855
82	2859337	2859436	reverse	nlpD,rpoS	-5195	0.42	99	ygbI	0.855
83	3490370	3490469	reverse	php,yhfS,yhfT,yhfU,yhfW,yhfX	-12538	0.32	99	ppiA	0.854
84	4220501	4220600	forward	aceA,aceB,aceK	-2097	0.37	1302	metH	0.853
85	715871	715970	reverse	potE,speF	-249	0.37	99	ybfG,ybfH	0.853
86	2461957	2462056	reverse	mlaA	-268	0.45	3049	yfcZ	0.852
87	2876502	2876601	reverse	cas1,cas2,casA,casB,casC,casD,casE	-40	0.34	2199	cysC,cysD,cysN	0.851
88	4084875	4084974	forward	fdhD	46	0.42	102	yiiG	0.85
89	4535630	4535729	forward	yjhR	-1527	0.31	3302	fimB	0.849
90	1635392	1635491	reverse	gnsB	-192	0.42	1049	nohA,tfaQ,ydfN	0.849
91	3497220	3497319	reverse	php,yhfS,yhfT,yhfU,yhfW,yhfX	-5688	0.33	6949	ppiA	0.849
92	3266938	3267037	reverse	garK,garL,garP,garR,rnpB	-1251	0.35	1899	tdcA,tdcB,tdcC,tdcD,tdcE,tdcF,tdcG	0.848
93	2267568	2267667	reverse	yejG	-8298	0.34	4299	yeiW	0.848
94	3580990	3581089	reverse	ggt	-2065	0.4	1999	ryhB	0.848
95	1811219	1811318	reverse	cedA	-177	0.5	99	ydjO	0.845
96	583210	583309	reverse	envY,ompT	-644	0.4	1299	ybcY	0.845
97	3576850	3576949	reverse	yhhW	-74	0.33	149	gntK,gntR,gntU	0.844
98	3266488	3266587	reverse	garK,garL,garP,garR,rnpB	-1701	0.43	1449	tdcA,tdcB,tdcC,tdcD,tdcE,tdcF,tdcG	0.843
99	2055603	2055702	reverse	asnW	-399	0.33	4299	yeeL_1,yeeL_2	0.843
100	2739273	2739372	reverse	rimM,rplS,rpsP,trmD	-2883	0.3	149	aroF,tyrA	0.842

Top 50 hits of the forward and reverse strands of the *E. coli* intergenic regions using 50 nt-overlap 100 nt window and under the LMFEGCRND model. The ranking of each hit is denoted in column R. Distance from upstream and downstream operons are the distance from the center of the hit to the stop and start codons of upstream and downstream operons, respectively. Probability denotes the multinomial regression likelihood of being a riboswitch under the LMFEGCRND model. Positions are according to gbU00096.2 version of *E. coli* and not gbU00096.3 version.

^1^Table 27: Complete list of genes in this operon is sdhA,sdhB,sdhC,sdhD,sucA,sucB,sucC,sucD.

**Table 28:** **Top classification hits in** ***E. coli*** **uracil-comp. constrained**

**R**	**Start**	**End**	**Strand**	**Upstream operon**	**Dist. to upstream**	**Uracil**	**Dist. to downstream**	**Downstream operon**	**Probability**
1	3886253	3886352	reverse	purP	-6993	0.31	4549	dnaA,dnaN,recF	0.876
2	3181557	3181656	reverse	ribB,sroG	-229	0.33	1149	ygiD	0.865
3	3755951	3756050	reverse	selA,selB	-40	0.33	149	yiaY	0.861
4	3490370	3490469	reverse	php,yhfS,yhfT,yhfU,yhfW,yhfX	-12538	0.32	99	ppiA	0.854
5	4535630	4535729	forward	yjhR	-1527	0.31	3302	fimB	0.849
6	3497220	3497319	reverse	php,yhfS,yhfT,yhfU,yhfW,yhfX	-5688	0.33	6949	ppiA	0.849
7	3576850	3576949	reverse	yhhW	-74	0.33	149	gntK,gntR,gntU	0.844
8	2055603	2055702	reverse	asnW	-399	0.33	4299	yeeL_1,yeeL_2	0.843
9	2739273	2739372	reverse	rimM,rplS,rpsP,trmD	-2883	0.3	149	aroF,tyrA	0.842
10	2698570	2698669	reverse	acpS,era,pdxJ,recO,rnc	-21	0.31	399	shoB	0.84
11	3945101	3945200	reverse	hdfR	-1	0.3	5799	hsrA,yieP	0.835
12	2739223	2739322	reverse	rimM,rplS,rpsP,trmD	-2933	0.31	99	aroF,tyrA	0.832
13	3453521	3453620	reverse	bfd,bfr	-10701	0.32	149	gspA,gspB	0.825
14	4274265	4274364	reverse	soxS	-769	0.32	1249	yjcB	0.822
15	1467282	1467381	forward	ydbA_1	-1259	0.32	52	insI-2	0.82
16	3116880	3116979	forward	pheV	-8368	0.33	2452	C0719	0.819
17	790896	790995	forward	aroG	-4939	0.26	3052	acrZ	0.817
18	2777069	2777168	forward	yfjX,yfjY,yfjZ,ypjF,ypjJ	-1216	0.33	7302	ygaQ_1,ygaQ_2	0.817
19	3189691	3189790	reverse	glgS	-21	0.31	6999	ribB,sroG	0.817
20	1694096	1694195	reverse	uidR	-341	0.33	49	uidA,uidB,uidC	0.815
21	2823699	2823798	reverse	norR	-5049	0.31	149	mltB	0.81
22	1805258	1805357	reverse	yniB	-1414	0.32	1199	ydiY	0.809
23	3382541	3382640	reverse	yhcO	-1289	0.33	299	mdh	0.809
24	1868697	1868796	reverse	yoaI	-3356	0.29	4249	mipA	0.808
25	2386449	2386548	reverse	nuoA^1^	-1572	0.31	49	yfbN	0.807
26	3617057	3617156	reverse	rbbA,yhhJ,yhiI	-6596	0.3	7349	yhhS	0.806
27	2815604	2815703	forward	micA	-2660	0.29	8202	gutM,gutQ,srlA,srlB,srlD,srlE,srlR	0.805
28	2943865	2943964	reverse	mltA	-189	0.31	49	tcdA	0.804
29	1983499	1983598	forward	uspC	-5245	0.31	1402	ftnB	0.803
30	150155	150254	forward	yadD	-2236	0.32	11902	hrpB	0.801
31	2553093	2553192	forward	amiA,hemF	-898	0.33	3652	intZ	0.8
32	2876452	2876551	reverse	cas1,cas2,casA,casB,casC,casD,casE	-90	0.31	2149	cysC,cysD,cysN	0.8
33	1217949	1218048	reverse	ymgD,ymgG	-3530	0.33	3299	bluF	0.8
34	3346238	3346337	reverse	elbB,mtgA	-816	0.33	8199	mlaB,mlaC,mlaD,mlaE,mlaF	0.798
35	1174989	1175088	reverse	ycfZ,ymfA	-4664	0.3	649	ycfT	0.798
36	4298873	4298972	forward	gltP	-5007	0.26	12452	rpiB	0.797
37	585173	585272	forward	appY	-1471	0.29	9602	cusA,cusB,cusC,cusF	0.796
38	1397665	1397764	forward	ynaJ	-1970	0.32	5052	abgR	0.795
39	4492546	4492645	forward	lptF,lptG	-6074	0.29	52	idnK	0.795
40	655892	655991	reverse	crcB	-837	0.32	749	dcuC	0.795
41	266028	266127	reverse	yafZ,ykfA	-331	0.28	299	yafW^2^	0.795
42	2033559	2033658	reverse	yedV,yedW	-1210	0.32	2099	yedJ,yedR	0.794
43	3963904	3964003	reverse	aslA	-18422	0.32	299	rhlB	0.794
44	1463517	1463616	reverse	insC-2,insCD-2,insD-2	-2379	0.33	11899	paaZ	0.794
45	2650116	2650215	forward	xseA	-16443	0.31	352	sseA	0.793
46	582654	582753	forward	tfaX	-322	0.3	202	appY	0.793
47	4076645	4076744	reverse	fdhE,fdoG,fdoH,fdoI	-1628	0.29	5099	yihS,yihT,yihU	0.793
48	2033409	2033508	reverse	yedV,yedW	-1360	0.32	1949	yedJ,yedR	0.793
49	1762598	1762697	forward	lpp	-6868	0.27	4452	ydiK	0.792
50	4501881	4501980	forward	yjgZ	-2220	0.32	152	yjhB,yjhC	0.792
51	1676251	1676350	forward	tqsA	-3231	0.33	152	ydgH	0.791
52	2166386	2166485	forward	cyaR	-1113	0.32	302	yegS	0.789
53	2429322	2429421	forward	folX,yfcH	-8650	0.28	6602	flk	0.789
54	4083889	4083988	forward	yiiF	-5848	0.29	102	fdhD	0.789
55	2491327	2491426	reverse	yfdX	-413	0.32	99	frc	0.789
56	1165025	1165124	reverse	comR	-2349	0.31	4299	fhuE	0.789
57	1933126	1933225	forward	purT	-2994	0.3	1502	yebK	0.787
58	1204407	1204506	reverse	stfE,tfaE	-3284	0.25	2299	ymfK	0.787
59	1493112	1493211	forward	trg	-929	0.32	152	ydcJ	0.786
60	1714050	1714149	forward	gstA	-995	0.31	3802	slyB	0.786
61	1397615	1397714	forward	ynaJ	-1920	0.32	5102	abgR	0.784
62	5034	5133	forward	thrA,thrB,thrC,thrL	35	0.27	152	yaaX	0.784
63	4547775	4547874	reverse	gntP	-152	0.25	10299	nanC,nanM	0.784
64	2257385	2257484	forward	yeiL	-3300	0.3	4452	setB	0.783
65	660791	660890	forward	tatE	-2369	0.29	13402	ybeL	0.781
66	3945051	3945150	reverse	hdfR	-51	0.29	5749	hsrA,yieP	0.781
67	2238382	2238481	forward	preA,preT	-3811	0.33	3502	yeiG	0.779
68	925014	925113	reverse	serW	-44	0.31	3249	cspD	0.779
69	13587	13686	reverse	hokC,mokC	-3115	0.3	1849	yaaI	0.779
70	2734984	2735083	reverse	aroF,tyrA	-1937	0.33	999	rluD,yfiH	0.778
71	2880686	2880785	forward	iap	-4997	0.32	9502	queD	0.777
72	1078128	1078227	forward	rutR	-3976	0.29	352	putP	0.776
73	187962	188061	forward	cdaR	-4293	0.32	1702	rpsB,tff,tsf	0.776
74	497037	497136	reverse	aes	-1152	0.25	7049	priC,ybaM	0.776
75	3181662	3181761	forward	zupT	-268	0.3	1152	yqiC	0.775
76	593123	593222	forward	appY	-9421	0.29	1652	cusA,cusB,cusC,cusF	0.775
77	1250189	1250288	forward	ycgY	-5317	0.33	52	dhaR	0.775
**78**	3597882	3597981	reverse	rpoH	-21	0.32	249	livJ	0.775
79	117883	117982	forward	guaC	-3347	0.33	802	ampD,ampE	0.774
80	1073265	1073364	forward	ymdF	-5739	0.31	152	rutR	0.774
81	3416188	3416287	reverse	alaU,ileU,rrfD,rrfF,rrlD,rrsD,thrV	-5208	0.28	4749	envR	0.774
82	4238098	4238197	forward	yjbE,yjbF,yjbG,yjbH	-296	0.33	202	psiE	0.773
83	3313859	3313958	forward	psrO	-4390	0.32	2752	argG	0.773
84	238253	238352	reverse	yafU	-444	0.29	2299	rnhA	0.773
85	2627711	2627810	reverse	guaA,guaB	-1220	0.31	799	yfgF	0.773
86	4156263	4156362	forward	argB,argC,argH	32	0.3	202	oxyR	0.772
87	58274	58373	forward	djlA	-46	0.32	152	yabP,yabQ	0.772
88	3108528	3108627	reverse	yghD,yghE	-35	0.33	1399	speC	0.772
89	2750731	2750830	reverse	ratA,ratB	-1250	0.31	2049	grpE	0.772
90	454057	454156	forward	bolA	5	0.32	252	tig	0.771
91	573621	573720	forward	ybcQ	-10	0.26	2952	essD,rrrD,rzpD	0.771
92	905496	905595	forward	amiD,ybjQ	-481	0.31	10152	ybjD	0.771
93	2407114	2407213	reverse	yfbS	-379	0.32	2499	lrhA	0.771
94	1733426	1733525	reverse	ydhP	-670	0.31	1349	grxD	0.771
95	604509	604608	forward	pheP	-1902	0.33	2502	hokE	0.77
96	1431895	1431994	forward	lomR_2,stfR,tfaR	-836	0.31	3202	micC	0.77
97	1395289	1395388	forward	insH-4	-124	0.28	52	ynaJ	0.769
98	253317	253416	forward	dinB,yafN,yafO,yafP	-107	0.3	102	prfH,ykfJ	0.768
99	573571	573670	forward	ybcQ	40	0.28	3002	essD,rrrD,rzpD	0.768
100	3986826	3986925	forward	glmZ	-2151	0.32	2302	cyaA	0.768

Top 50 hits of the forward and reverse strands of the *E. coli* intergenic regions that have uracil composition within the range of known riboswitches in *E. coli* (between 0.23 and 0.34). 50 nt-overlap 100 nt window used. The ranking of each hit is denoted in column R. Distance from upstream and downstream operons are the distance from the center of the hit to the stop and start codons of upstream and downstream operons, respectively. Probability denotes the multinomial regression likelihood of being a riboswitch under the LMFEGCRND model. Positions are according to gbU00096.2 version of *E. coli* and not gbU00096.3 version.

^1^Table 28: Complete list of genes in this operon is nuoA,nuoB,nuoC,nuoE,nuoF,nuoG,nuoH,nuoI,nuoJ,nuoK,nuoL,nuoM,nuoN. ^2^Table 28: Complete list of genes in this operon is yafW,yafX,yafY,ykfB,ykfF,ykfG,ykfH,ykfI.

Interesting hits are shown in bold.

**Table 29:** **Top entropy hits of** ***E. coli*** **filtered for GC- and uracil-comp**

***E. coli***	**Start**	**End**	**Strand**	**Upstream operon**	**Dist. to upstream**	**MFE**	**MFE p. Val.**	**GC**	**RND**	**RND p. Val.**	**Uracil**	**Dist. to downstream**	**Downstream operon**	**Probability**
100 nt	4083889	4083988	forward	yiiF	-5848	-38.4	0.0267	0.53	58.6367989	0.0365	0.29	102	fdhD	0.789
100 nt	187962	188061	forward	cdaR	-4293	-36.4	0.0466	0.53	59.0985985	0.0229	0.32	1702	rpsB,tff,tsf	0.776
100 nt	952485	952584	forward	ycaK	-2955	-36.8	0.0419	0.52	58.3203011	0.0494	0.27	3452	ycaP	0.765
100 nt	4115038	4115137	forward	uspD,yiiS	-3245	-37	0.0396	0.53	58.3563995	0.0477	0.33	1452	zapB	0.756
***E. coli***	**Start**	**End**	**Strand**	**Upstream operon**	**Dist. to upstream**	**MFE**	**MFE p. Val.**	**GC**	**RND**	**RND p. Val.**	**Uracil**	**Dist. to downstream**	**Downstream operon**	**Probability**
150 nt	2686923	2687072	forward	hmp	-1802	-56.00	-	0.5333	90.7522964	0.0077	0.32000	6827	mltF	0.8671584129
150 nt	2887386	2887535	forward	iap	-11672	-56.40	-	0.5333	89.1240005	-	0.0294	2777	queD	0.8254097700
150 nt	3467187	3467336	forward	gspO^1^	-2871	-56.10	-	0.5200	88.5419006	0.0450	0.29333	8402	slyX	0.8172816634
150 nt	3576825	3576974	reverse	yhhW	-74	-55.60	-	0.4800	88.6371994	0.0419	0.30666	149	gntK,gntR,gntU	0.8547886610
150 nt	2195866	2196015	reverse	yehS	-13808	-58.00	-	0.5333	88.6897964	0.0405	0.27333	3749	mrp	0.8320623040

Significant hits of the forward and reverse strands of the *E. coli* intergenic regions having significantly high RND entropy (p-Val. <0.0500), significantly low (p.Val. <0.050), GC and uracil compositions within the range of those for known riboswitches Threshold values and their corresponding p-values have been calculated separately for each genome-wide test. 50 nt overlap used for 100 nt scan (100090 segments). 175 nt overlap used for 150 nt scan (66414 segments). Distance from Upstream and Downstream operons are the distance from the center of the hit to the stop and start codons of upstream and downstream operons, respectively. Probability denotes the multinomial regression likelihood of being a riboswitch under the LMFEGCRND model. Positions are according to gbU00096.2 version of *E. coli* and not gbU00096.3 version. Negative values indicate distance to upstream operon. Columns Upsream/Downstream Operon show gene ID within the operon.

^1^Table 29: Complete list of genes in this operon is gspC,gspD,gspE,gspF,gspG,gspH,gspI,gspJ,gspK,gspL,gspM,gspO.

**Table 30:** **Top entropy hits in** ***E. coli***

***E. coli***	**Start**	**End**	**Strand**	**Upstream operon**	**Dist. to upstream**	**MFE**	**MFE p. Val.**	**GC**	**RND**	**RND p. Val.**	**Uracil**	**Dist. to downstream**	**Downstream operon**	**Probability**
100 nt	4083889	4083988	forward	yiiF	-5848	-38.4	0.0267	0.53	58.6367989	0.0365	0.29	102	fdhD	0.789
100 nt	187962	188061	forward	cdaR	-4293	-36.4	0.0466	0.53	59.0985985	0.0229	0.32	1702	rpsB,tff,tsf	0.776
100 nt	952485	952584	forward	ycaK	-2955	-36.8	0.0419	0.52	58.3203011	0.0494	0.27	3452	ycaP	0.765
100 nt	4115038	4115137	forward	uspD,yiiS	-3245	-37	0.0396	0.53	58.3563995	0.0477	0.33	1452	zapB	0.756
***E. coli***	**Start**	**End**	**Strand**	**Upstream operon**	**Dist. to upstream**	**MFE**	**MFE p. Val.**	**GC**	**RND**	**RND p. Val.**	**Uracil**	**Dist. to downstream**	**Downstream operon**	**Probability**
150 nt	2686923	2687072	forward	hmp	-1797	-56.00	-	0.5333	90.7522964	0.0077	0.32000	6822	mltF	0.8671584129
150 nt	452721	452870	forward	yajQ	-8244	-60.90	-	0.5200	88.2920990	-	0.23333	897	bolA	0.8664909005
150 nt	1100699	1100848	forward	ycdZ	-610	-58.70	-	0.4933	87.8781967	-	0.30666	2397	csgA,csgB,csgC	0.8559710383
150 nt	2887386	2887535	forward	iap	-11667	-56.40	-	0.5333	89.1240005	0.0294	0.31333	2772	queD	0.8254097700
150 nt	3467187	3467336	forward	gspO^1^	-2866	-56.10	-	0.5200	88.5419006	0.0450	0.29333	8397	slyX	0.8172816634
150 nt	2553118	2553267	forward	amiA,hemF	-893	-57.40	-	0.5133	87.6467972	-	0.28666	3597	intZ	0.8125300407
150 nt	2660264	2660413	forward	ryfA	-8005	-56.90	-	0.5000	87.1239014	-	0.32000	1122	suhB	0.8031908870
150 nt	1766798	1766947	forward	lpp	-11038	-57.00	-	0.4800	85.6548004	-	0.25333	222	ydiK	0.7757616639
150 nt	1718374	1718523	forward	slyB	72	-58.70	-	0.4867	85.1240005	-	0.32666	597	ydhI,ydhJ,ydhK	0.7731205821
150 nt	4356712	4356861	forward	yjdK,yjdO	-5529	-58.80	-	0.5200	86.0333023	-	0.26000	9897	fxsA	0.7661048174
150 nt	149580	149729	forward	yadD	-1631	-57.80	-	0.4600	84.3807983	-	0.30666	12447	hrpB	0.7651519775
150 nt	4604476	4604625	forward	yjjZ	-334	-57.20	-	0.4867	85.4507980	-	0.27333	1272	holD,rimI,yjjG	0.7621335387
150 nt	3120069	3120218	forward	C0719	-389	-56.40	-	0.5267	87.1032028	-	0.27333	6147	glcC	0.7610746622
150 nt	1982024	1982173	forward	uspC	-3740	-56.20	-	0.5333	87.3750000	-	0.26666	2847	ftnB	0.7596676350
150 nt	3921878	3922027	forward	cbrB,cbrC	-25167	-56.00	-	0.4933	85.5883026	-	0.30666	3222	asnA	0.7384917736
150 nt	790921	791070	forward	aroG	-4934	-57.00	-	0.5333	86.4469986	-	0.28000	2997	acrZ	0.7345629930
150 nt	4482291	4482440	forward	yjgN	-3263	-58.30	-	0.5200	85.4577026	-	0.28666	1872	lptF,lptG	0.7340587974
150 nt	518357	518506	forward	ybbL,ybbM	-1692	-57.10	-	0.5000	85.1729965	-	0.26000	522	ybbA,ybbP	0.7300664783
150 nt	1167546	1167695	forward	ycfJ	-106	-57.70	-	0.5333	85.9982986	-	0.27333	672	bhsA	0.7274026275
150 nt	1642496	1642645	forward	cspF	-2326	-56.00	-	0.5333	86.4957962	-	0.25333	1347	ydfV	0.7190257311
150 nt	3916103	3916252	forward	cbrB,cbrC	-19392	-60.90	-	0.5200	84.0416031	-	0.30000	8997	asnA	0.7181233764
150 nt	3258127	3258276	forward	yhaK,yhaL	-4819	-55.70	-	0.5267	86.1580963	-	0.26000	7197	tdcR	0.7085512877
150 nt	3721360	3721509	forward	insK	-1209	-57.70	-	0.5133	84.8648987	-	0.26000	2472	wecH	0.7070741653
150 nt	2438211	2438360	forward	flk	-1165	-58.50	-	0.5267	84.9561996	-	0.24000	1497	mnmC	0.7053987384
150 nt	1268246	1268395	forward	kdsA,ychA,ychQ	75	-56.90	-	0.4933	84.3839035	-	0.29333	222	rdlA	0.7012539506
150 nt	219458	219607	forward	arfB,nlpE,yaeQ	-3400	-59.90	-	0.5267	84.2009964	-	0.27333	3297	gmhB	0.6966923475
150 nt	3514668	3514817	forward	frlA,frlB,frlC,frlD,frlR	-11784	-56.50	-	0.5333	85.6490021	-	0.27333	6147	mrcA	0.6895118356
150 nt	3313884	3314033	forward	psrO	-4385	-55.60	-	0.5067	84.9284973	-	0.28666	2697	argG	0.6809250712
150 nt	649808	649957	forward	ybdR	-7180	-58.10	-	0.5333	84.6990967	-	0.25333	1572	dpiA,dpiB	0.6750817299
150 nt	2243989	2244138	forward	yeiG	-1142	-57.10	-	0.5333	84.3582993	-	0.26000	3672	yeiH	0.6381362677
150 nt	4253685	4253834	forward	ubiA,ubiC	-1695	-55.80	-	0.5267	84.4711990	-	0.29333	897	dgkA	0.6285824776
150 nt	2866503	2866652	forward	ygbN	-1937	-56.80	-	0.5333	84.2586975	-	0.30000	8022	iap	0.6268372536
150 nt	3576825	3576974	reverse	yhhW	-69	-55.60	-	0.4800	88.6371994	0.0419	0.30666	154	gntK,gntR,gntU	0.8547886610
150 nt	260750	260899	reverse	yafW,yafX,yafY,ykfB,ykfF,ykfG,ykfH,ykfI	-1723	-62.00	-	0.5267	86.8554001	-	0.32000	1504	phoE	0.8323625326
150 nt	2195866	2196015	reverse	yehS	-13803	-58.00	-	0.5333	88.6897964	0.0405	0.27333	3754	mrp	0.8320623040
150 nt	4176725	4176874	reverse	sroH	-11546	-58.80	-	0.5333	88.1481018	-	0.28000	3754	coaA	0.8252514601
150 nt	133211	133360	reverse	speD,speE,yacC	-1498	-56.40	-	0.5133	88.0559998	-	0.28000	2029	yacH	0.8126859665
150 nt	44332	44481	reverse	apaG,apaH,lptD,pdxA,rsmA,surA	-5969	-64.00	-	0.5333	85.3160019	-	0.26000	2479	caiA,caiB,caiC,caiD,caiE,caiT	0.8009238243
150 nt	2248916	2249065	reverse	nupX	-1922	-55.70	-	0.5333	88.3962021	-	0.28000	1354	yeiE	0.7910096645
150 nt	2774331	2774480	reverse	ypjA	-1758	-55.60	-	0.5200	87.4978027	-	0.26666	3229	ypjM_1,ypjM_2	0.7727379203
150 nt	2184269	2184418	reverse	yehA,yehB,yehC,yehD	-1054	-56.20	-	0.5333	87.6942978	-	0.30666	529	rcnR	0.7722578049
150 nt	1655707	1655856	reverse	mlc,ynfK	-8762	-58.20	-	0.5267	86.6440964	-	0.24000	304	ynfC	0.7716723680
150 nt	2168389	2168538	reverse	gatR_2	-951	-56.70	-	0.5133	86.7958984	-	0.31333	304	gatR_1	0.7715415359
150 nt	3481959	3482108	reverse	yhfA	-1398	-57.90	-	0.5333	86.7799988	-	0.25333	2854	kefB,kefG,yheV	0.7633723617
150 nt	314642	314791	reverse	ykgA	-953	-58.20	-	0.5067	85.1943970	-	0.32000	2254	ykgR	0.7403761148
150 nt	2783559	2783708	reverse	ileY	-146	-56.20	-	0.4667	84.4744034	-	0.26666	604	ypjC	0.7329583764
150 nt	4050403	4050552	reverse	glnA,glnG,glnL	-1410	-56.70	-	0.5267	86.2409973	-	0.27333	1729	yihA	0.7301918864
150 nt	2465055	2465204	reverse	yfdK,yfdL,yfdM,yfdN,yfdO	-3965	-58.50	-	0.5333	85.6035004	-	0.25333	2104	mlaA	0.7242872119
150 nt	2809717	2809866	reverse	luxS	-2444	-56.20	-	0.5333	86.4486008	-	0.29333	11779	ygaC	0.7205215693
150 nt	3341463	3341612	reverse	elbB,mtgA	-5561	-57.50	-	0.5000	84.6742020	-	0.28000	3454	mlaB,mlaC,mlaD,mlaE,mlaF	0.7152099609
150 nt	1950122	1950271	reverse	torY,torZ	-2401	-56.44	-	0.5200	85.7341003	-	0.24666	1654	aspS	0.7131192684
150 nt	3059434	3059583	reverse	ygfI	-4786	-56.20	-	0.4933	84.9087982	-	0.27333	1879	yqfE	0.7122740149
150 nt	2530006	2530155	reverse	pdxK	-4323	-58.20	-	0.5000	84.2724991	-	0.25333	829	zipA	0.7098694444
150 nt	757742	757891	reverse	mngR	-6555	-57.90	-	0.4867	83.9291000	-	0.31333	4129	gltA	0.7092900276
150 nt	4452551	4452700	reverse	fbp	-4	-56.90	-	0.5200	85.3691025	-	0.29333	4954	ppa	0.7049999237
150 nt	3584997	3585146	reverse	ugpA,ugpB,ugpC,ugpE,ugpQ	-317	-59.30	-	0.5133	84.1650009	-	0.32000	229	ggt	0.7046704292
150 nt	3459796	3459945	reverse	bfd,bfr	-4396	-59.60	-	0.5200	84.1896973	-	0.23333	6454	gspA,gspB	0.7009978294
150 nt	3395856	3396005	reverse	mreB,mreC,mreD	-474	-56.20	-	0.5000	84.8627014	-	0.25333	1654	yhdP	0.6998521686
150 nt	2321601	2321750	reverse	yfaA,yfaP,yfaQ,yfaS_1,yfaS_2,yfaT	-3709	-61.10	-	0.5333	83.9000015	-	0.24000	3829	rcsC	0.6947578788
150 nt	4169919	4170068	reverse	coaA	-2101	-56.20	-	0.4933	84.4697037	-	0.26000	8704	trmA	0.6920779943
150 nt	4029970	4030119	reverse	mobA,mobB	-8880	-57.80	-	0.5133	84.4968033	-	0.30000	1054	fadA,fadB	0.6919249892
150 nt	2366493	2366642	reverse	pmrD	-4722	-56.00	-	0.5200	85.1843033	-	0.28000	2929	ais	0.6792802215
150 nt	1850076	1850225	reverse	ydjE	-490	-57.40	-	0.5267	84.6417999	-	0.27333	3454	selD,topB,ydjA	0.6695327163
150 nt	3246739	3246888	reverse	yhaJ	-4522	-56.00	-	0.4867	83.8035965	-	0.26000	4054	uxaA,uxaC	0.6671289802
150 nt	3883703	3883852	reverse	purP	-9513	-56.60	-	0.5200	84.5989990	-	0.28000	2029	dnaA,dnaN,recF	0.6626442075
150 nt	4395241	4395390	reverse	yjfN	-18720	-56.20	-	0.5267	84.9857025	-	0.29333	3229	queG	0.6626062393
150 nt	2664571	2664720	reverse	hcaT	-79	-56.20	-	0.5267	84.7899017	-	0.24666	3304	trmJ	0.6529098153
150 nt	2975533	2975682	reverse	lysA	-47	-57.20	-	0.5333	84.5955963	-	0.24666	1204	omrB	0.6521243453
150 nt	796718	796867	reverse	ybhA	-39	-58.40	-	0.5333	83.8794022	-	0.26666	2929	modE,modF	0.6405209899
150 nt	3288088	3288237	reverse	rsmI	-2330	-55.90	-	0.5067	83.9057007	-	0.28000	11479	agaR	0.6363855004
150 nt	3211791	3211940	reverse	mug	-1119	-56.10	-	0.5200	84.1829987	-	0.25333	3304	tsaD	0.6315983534
150 nt	2161503	2161652	reverse	ogrK	-3744	-55.70	-	0.5333	84.3114014	-	0.29333	10429	yegK,yegL	0.6064385772
150 nt	3284413	3284562	reverse	rsmI	-6005	-55.60	-	0.5333	84.2777023	-	0.25333	7804	agaR	0.6025381684

Significant hits of the forward and reverse strands of the *E. coli* intergenic regions having high RND entropy (p-Val.<0.500), significantly low (p.Val. <0.050), GC and uracil compositions within the range of those for known riboswitches Threshold values and their corresponding p-values have been calculated separately for each genome-wide test. 50 nt overlap used for 100 nt scan (100090 segments). 175 nt overlap used for 150 nt scan (66414 segments). Distance from upstream and downstream operons are the distance from the center of the hit to the stop and start codons of upstream and downstream operons, respectively. Probability denotes the multinomial regression likelihood of being a riboswitch under the LMFEGCRND model. Positions are according to gbU00096.2 version of *E. coli* and not gbU00096.3 version. Negative values indicate distance to upstream operon. Columns Upsream/Downstream Operon show gene ID within the operon.

^1^Table 30: Complete list of genes in this operon is gspC,gspD,gspE,gspF,gspG,gspH,gspI,gspJ,gspK,gspL,gspM,gspO.

*Synechococcus elongatus* is another gram-negative bacterium which belongs to cyanobacteria. This organism is able to survive in freshwater environments with low nutrients. Intergenic regions of the sequenced strain *Synechococcus elongatus* PCC 6301 were scanned for riboswitch identification using the LMFEGCRND classifier with sliding window of 150 nt and 75 nt overlaps. Scanning procedure was similar to other organisms. The resulting top 100 hits (top 50 hits of the forward and reverse strands) are sorted in Table 31 according to their classification probabilities of being riboswitches under the LMFEGCRND classifier.

**Table 31:** **Top classification hits in** ***S. elongatus***

**R**	**Start**	**End**	**Strand**	**Upstream operon**	**Dist. to upstream**	**Uracil**	**Dist. to downstream**	**Downstream operon**	**Probability**
1	2239899	2240048	forward	psbV	-2288	0.34666	1277	coaD	0.9169
2	1788275	1788424	reverse	syc1657_c	-1875	0.19333	5099	syc1649_c	0.9156
3	1673354	1673503	forward	accB	-811	0.22666	3377	desC	0.9078
4	734659	734808	forward	syc0660_d	-635	0.24666	8177	syc0665_d	0.9004
5	1108388	1108537	reverse	lacF	-1124	0.41333	1124	devB	0.8967
6	838999	839148	forward	gst1	-2009	0.20666	2552	hisG	0.8906
7	734734	734883	forward	syc0660_d	-710	0.28	8102	syc0665_d	0.886
8	1674179	1674328	forward	accB	-1636	0.28	2552	desC	0.8857
9	936254	936403	forward	syc0839_d	-190	0.26	77	syc0840_d	0.8855
10	1706450	1706599	forward	syc1578_d	-2580	0.28	5252	gpsA	0.8834
11	2686971	2687120	reverse	syc2516_c	-462	0.24666	674	ycf49	0.8809
12	464795	464944	forward	syc0415_d	-1330	0.46	377	syc0416_d	0.8784
13	546798	546947	reverse	syc0485_c	-190	0.26666	74	syc0484_c	0.8778
14	1527281	1527430	reverse	holB	-916	0.34	6674	syc1405_c	0.8749
15	2251075	2251224	reverse	masA	-985	0.25333	674	syc2098_c	0.8741
16	590779	590928	reverse	syc0539_c	-11831	0.2	2624	syc0528_c	0.8733
17	1870479	1870628	forward	syc1725_d	-722	0.21333	5102	syc1732_d	0.873
18	2098418	2098567	forward	sui1	-7782	0.27333	2252	dapB	0.8715
19	793922	794071	reverse	asnS	-1542	0.46666	224	syc0707_c	0.8703
20	1218955	1219104	reverse	trpF	-430	0.34	1799	sycRNA024_c	0.8703
21	1751997	1752146	reverse	syc1625_c	-3772	0.32666	3899	syc1619_c	0.8691
22	1526981	1527130	reverse	holB	-1216	0.32	6374	syc1405_c	0.8686
23	1120163	1120312	reverse	syc1011_c	-4710	0.22	3749	syc1002_c	0.8684
24	69102	69251	reverse	syc0062_c	-2424	0.18666	974	syc0058_c	0.8668
25	132050	132199	reverse	argC	-633	0.21333	7724	syc0114_c	0.8668
26	1348202	1348351	reverse	syc1238_c	-2618	0.22	1049	syc1235_c	0.8666
27	1674104	1674253	forward	accB	-1561	0.26666	2627	desC	0.8663
28	479806	479955	forward	syc0423_d	-3318	0.34666	302	syc0428_d	0.8656
29	2009813	2009962	forward	syc1856_d	36	0.29333	1352	syc1859_d	0.8647
30	1674554	1674703	forward	accB	-2011	0.27333	2177	desC	0.8646
31	1372776	1372925	reverse	syc1262_c	-154	0.28666	824	gyrA	0.863
32	702997	703146	forward	syc0629_d	-3402	0.27333	3452	acnB	0.8622
33	1673054	1673203	forward	accB	-511	0.29333	3677	desC	0.8622
34	971765	971914	reverse	priA	-1871	0.3	1199	psbDI	0.861
35	2574891	2575040	forward	syc2417_d	-956	0.21333	6227	syc2423_d	0.8585
36	2033882	2034031	forward	prfA	38	0.26	527	alr	0.8579
37	2633236	2633385	forward	syc2468_d	-1767	0.22666	902	rps4	0.8555
38	2031022	2031171	reverse	mrcA	-2764	0.22	14249	syc1864_c	0.8549
39	1706375	1706524	forward	syc1578_d	-2505	0.29333	5327	gpsA	0.8543
40	69327	69476	reverse	syc0062_c	-2199	0.21333	1199	syc0058_c	0.8532
41	2383320	2383469	forward	syc2228_d	-2586	0.26	1052	natB	0.8531
42	2162031	2162180	forward	syc2015_d	-852	0.33333	5402	pilB	0.8527
43	1848963	1849112	forward	syc1711_d	-916	0.23333	6527	syc1716_d	0.8521
44	437513	437662	forward	syc0392_d	-5939	0.35333	677	syc0396_d	0.8509
45	1108838	1108987	reverse	lacF	-674	0.30666	1574	devB	0.8508
46	1763242	1763391	forward	syc1627_d	-4972	0.26	5027	aroE	0.8504
47	298041	298190	reverse	syc0257_c	-92	0.40666	224	syc0256_c	0.8502
48	1357802	1357951	reverse	uppS	-635	0.22	749	gidB	0.8501
49	1674704	1674853	forward	accB	-2161	0.34666	2027	desC	0.8495
50	2225318	2225467	reverse	syc2077_c	-5809	0.26666	1724	rfaG	0.8493
51	1967731	1967880	reverse	amt1	-197	0.26	974	syc1819_c	0.8483
52	1108913	1109062	reverse	lacF	-599	0.34666	1649	devB	0.848
53	2664277	2664426	forward	syc2500_d	-688	0.24666	1502	pcrA	0.8477
54	838924	839073	forward	gst1	-1934	0.18	2627	hisG	0.8469
55	1996234	1996383	reverse	sycRNA048_c	-2339	0.26	4799	syc1843_c	0.8453
56	1674479	1674628	forward	accB	-1936	0.29333	2252	desC	0.8435
57	1320549	1320698	reverse	ssb	-269	0.26	1349	syc1209_c	0.8421
58	542490	542639	forward	syc0480_d	-2611	0.28	19127	syc0503_d	0.8419
59	1089898	1090047	reverse	nhaS4	-886	0.2	974	syc0972_c	0.8417
60	2383395	2383544	forward	syc2228_d	-2661	0.27333	977	natB	0.8416
61	2201969	2202118	reverse	syc2052_c	-3184	0.32666	3074	gcvH	0.8408
62	2475474	2475623	forward	syc2315_d	-359	0.28666	4427	syc2322_d	0.8399
63	1910646	1910795	forward	psaL	-4022	0.21333	752	syc1766_d	0.8391
64	2244572	2244721	forward	syc2089_d	-2066	0.26	902	syc2094_d	0.8378
65	2202119	2202268	reverse	syc2052_c	-3034	0.33333	3224	gcvH	0.8378
66	979835	979984	reverse	hemL	-205	0.31333	149	syc0880_c	0.8371
67	2201519	2201668	reverse	syc2052_c	-3634	0.32	2624	gcvH	0.8366
68	2436792	2436941	forward	htpG	-4101	0.35333	3227	syc2285_d	0.8358
69	2379191	2379340	forward	syc2225_d	-348	0.22666	977	syc2228_d	0.8347
70	751797	751946	forward	syc0670_d	-2744	0.20666	3527	syc0679_d	0.8335
71	2488156	2488305	forward	syc2327_d	-3334	0.24666	2402	syc2333_d	0.8333
72	1099747	1099896	forward	syc0978_d	-5042	0.31333	1727	psaC	0.8327
73	316592	316741	forward	moaA	-195	0.38	302	syc0272_d	0.8319
74	979910	980059	reverse	hemL	-130	0.29333	224	syc0880_c	0.8319
75	636787	636936	forward	prk	-916	0.22	1802	syc0569_d	0.8318
76	1709000	1709149	forward	syc1578_d	-5130	0.26666	2702	gpsA	0.8317
77	888391	888540	forward	syc0774_d	-22228	0.19333	14402	syc0808_d	0.8304
78	542940	543089	forward	syc0480_d	-3061	0.29333	18677	syc0503_d	0.8303
79	1736496	1736645	reverse	gyrB	-7265	0.24666	1724	aroF	0.83
80	542265	542414	forward	syc0480_d	-2386	0.28	19352	syc0503_d	0.8299
81	482179	482328	reverse	syc0432_c	-3508	0.2	2249	rpl33	0.8299
82	882016	882165	forward	syc0774_d	-15853	0.2	20777	syc0808_d	0.8297
83	2239824	2239973	forward	psbV	-2213	0.32666	1352	coaD	0.8293
84	55214	55363	forward	syc0043_d	-1501	0.24666	4802	syc0049_d	0.8292
85	707052	707201	reverse	syc0639_c	-3061	0.22	824	syc0636_c	0.8292
86	992221	992370	reverse	syc0895_c	-5003	0.34	2099	eno	0.8289
87	234289	234438	reverse	syc0202_c	-357	0.38666	149	syc0201_c	0.828
88	1674629	1674778	forward	accB	-2086	0.29333	2102	desC	0.8278
89	1788200	1788349	reverse	syc1657_c	-1950	0.16	5024	syc1649_c	0.8273
90	1084661	1084810	reverse	nfrC	-959	0.21333	449	syc0967_c	0.8273
91	131975	132124	reverse	argC	-708	0.22	7649	syc0114_c	0.8271
92	632779	632928	reverse	petH	-327	0.32666	3599	syc0562_c	0.8269
93	2612915	2613064	reverse	syc2455_c	-5717	0.28666	2624	syc2447_c	0.8266
94	539354	539503	reverse	pdhC	-603	0.22666	3074	syc0476_c	0.8263
95	2360335	2360484	reverse	syc2221_c	-10564	0.19333	899	recJ	0.8245
96	503343	503492	reverse	syc0452_c	-2439	0.26	1274	syc0448_c	0.8242
97	621230	621379	reverse	syc0558_c	-3271	0.20666	299	syc0552_c	0.8195
98	2077186	2077335	reverse	syc1942_c	-5748	0.18666	1349	apcF	0.8187
99	971165	971314	reverse	priA	-2471	0.28	599	psbDI	0.8163
100	1555584	1555733	reverse	syc1449_c	-12609	0.35333	8549	pepP	0.815

Top 50 hits of the forward and reverse strands of the *S. elongatus* intergenic regions using 75 nt-overlap 150 nt window and under the LMFEGCRND model. The ranking of each hit is denoted in column R. Distance from upstream and downstream operons are the distance from the center of the hit to the stop and start codons of upstream and downstream operons, respectively. Probability denotes the multinomial regression likelihood of being a riboswitch under the LMFEGCRND model.

### Genome-wide scan results: tables

Classification performance on the *B. subtilis* riboswitches is shown in Table 16. Classification performance on *B. subtilis* riboswitches using different choices of length is shown in Table 17. Performance values for the choice of 157 nt as the riboswitch length are shown in Tables 18 and 19 for maximum overlap and relative positioning, respectively. Ranking of probabilities associated with different riboswitches are shown in Tables 20 and 21. Genome-wide scan results for *B. subtilis* are shown in Tables 22, 23, 24, 25 and 26 (please refer to Table captions for further explanation). Results for *E. coli* are shown in Tables 27, 28, 29 and 30. Results for *S. elongatus* are shown in Table 31.
